# Relationship Between Social Isolation, Loneliness, and Systemic Inflammation: A Scoping Review of Biological Signatures and Clinical Outcomes

**DOI:** 10.3390/diseases14100388

**Published:** 2026-10-21

**Authors:** Aimilia Bechliouli, Dimitrios Stylianakis, Marios Spanakis, Christos Lionis, Emmanouil K. Symvoulakis

**Affiliations:** 1 School of Medicine, University of Crete, 71003 Heraklion, Greece; med4p1030452@med.uoc.gr; 2 School of Medicine, University Hospital and University of Crete, 70013 Heraklion, Greece; 3 Department of Social Medicine, School of Medicine, University of Crete, 70013 Heraklion, Greece; mspanakis@uoc.gr (M.S.); lionis@uoc.gr (C.L.); esymvoulakis@uoc.gr (E.K.S.); 4 Community Pharmacists Association of Heraklion, 71201 Heraklion, Greece

**Keywords:** social isolation, loneliness, inflammation, social connectedness, biomarkers, clinical outcomes

## Abstract

**Highlights:**

- CRP and IL-6 were the most investigated biomarkers, with isolation more consistently linked to CRP/fibrinogen, and loneliness more consistently linked to IL-6.
- Social isolation was associated with increased risk of sarcopenia, venous thromboembolism, pancreatic cancer, and cardiovascular disease, with inflammatory or metabolic markers mediating a modest (≈5–19%) fraction of these associations.
- Loneliness- and isolation-associated plasma proteomic signatures (*n* = 42,062) implicated inflammatory, antiviral, and complement pathways; more than half of the associated proteins prospectively predicted cardiometabolic disease and mortality.
- Social connectedness and positive relationship quality were associated with lower IL-6 and better functional outcomes, independent of global network size.
- Heterogeneity in psychosocial measurement and study design was high, underscoring the need for harmonized assessment tools and prospective designs.

**Abstract:**

Background: Social isolation and loneliness are established social determinants of health, associated with 25–30% higher mortality risk, yet their relationship with systemic inflammation and broader biological and clinical outcomes has not been comprehensively synthesized across life stages. Methods: PubMed/MEDLINE, Scopus, and ScienceDirect were searched (2015–2025), supplemented by citation searching and a targeted supplementary search, for human studies linking social isolation, loneliness, or social connectedness to inflammatory biomarkers or biological/clinical outcomes. PRISMA-ScR guidance was followed; findings were synthesized narratively across five thematic axes as an evidence map. Results: Of 3351 records identified, 73 studies were included. Isolation was most often associated with CRP and fibrinogen, and loneliness with IL-6. Isolation predicted sarcopenia, venous thromboembolism, pancreatic cancer, and cardiovascular disease, with inflammatory or metabolic markers mediating a modest (≈5–19%) share. Proteomic (*n* = 42,062) and epigenetic analyses linked loneliness to inflammatory, antiviral, and complement-pathway proteins and to accelerated epigenetic ageing; Mendelian randomization supported causal involvement of several proteins. Social connectedness and relationship quality tracked with lower IL-6 and better functional indices. Evidence was strongest in midlife and older cohorts. Conclusions: Social disconnection shows biologically plausible but heterogeneous associations with inflammatory and molecular signatures; harmonized measurement and prospective, hypothesis-free omics designs are needed to clarify causality and inform targeted screening.

## 1. Introduction

Social isolation and loneliness are now recognized as major social determinants of health, with meta-analytic evidence linking social disconnection to approximately 25–30% higher mortality risk [1,2]. Recent syntheses in older adults confirm that loneliness and small social networks predict all-cause mortality, and major public-health bodies, including the U.S. Surgeon General, now frame this as an “epidemic” warranting attention comparable to tobacco or obesity [3,4,5].

Converging biological research suggests that these risks reflect sustained stress and inflammatory dysregulation. Chronic loneliness is proposed to act as a multisystem stressor, driving allostatic load, HPA-axis dysregulation, and immune–metabolic changes [6,7]. A systematic review found loneliness associated with elevated IL-6 in fully adjusted models, while isolation links to CRP and fibrinogen, though results remain mixed [8]. Daily-life studies show short-term loneliness fluctuations track with peripheral inflammatory changes [9].

Newer work reveals deeper life-course biological embedding: multi-cohort analyses link childhood and midlife isolation to suPAR, a stable marker of chronic inflammation more predictive than CRP or IL-6 [10]. Loneliness also relates to DNA methylation age acceleration, with GrimAge and DunedinPACE partly mediating links to chronic disease [11,12]. Overall, social disconnection shows measurable correlates spanning stress response, inflammation, and molecular ageing, though effect sizes are modest, and causality is uncertain.

Despite the expanding literature, most studies focus on single endpoints or specific age groups, rarely integrating objective isolation, subjective loneliness, and broader connectedness within one framework. Measurement heterogeneity, cross-sectional designs, and incomplete confounder adjustment have produced inconsistent findings, and clinical guidelines rarely address when or how to assess social disconnection biologically. This review therefore provides a structured, PRISMA-compliant synthesis of how social isolation, loneliness, and social connectedness relate to inflammatory biomarkers and broader biological/clinical outcomes across the life course, organized into five thematic axes distinguishing structural isolation, subjective loneliness, and social connectedness.

## 2. Methods

### 2.1. Study Design and Reporting

This scoping review followed the Preferred Reporting Items for Systematic Reviews and Meta-Analyses Extension for Scoping Reviews (PRISMA-ScR) statement [13]. A PRISMA-ScR checklist is provided as Supplementary Table S5. No protocol was registered for this review; the implications of this are addressed in the Limitations section. Eligibility criteria, screening, and extraction followed a systematic structure, though the breadth of eligible outcomes—spanning inflammatory biomarkers, functional measures, and clinical endpoints—introduces elements beyond a single outcome domain; this review is therefore framed as an evidence map rather than as a basis for weighted, pooled conclusions.

### 2.2. Eligibility Criteria and Research Question

Eligibility followed a Population, Exposure, Comparator, and Outcome (PECO) framework adapted for psychosocial epidemiology. Eligible populations comprised human participants of any age in community or clinical settings, with no geographic restriction. The exposure was social isolation, loneliness, or social connectedness; however, it was operationalized (objective network structure, subjective perceived isolation, or composite engagement indices). Comparators were participants with lower exposure severity or standard social integration. Outcomes included any inflammatory biomarker (CRP, IL-6, TNF, and fibrinogen), related biological indices (proteomic, genomic, and neuroendocrine), or clinical/functional sequelae plausibly linked to inflammatory mediation. Studies required English-language publication between 1 January 2015 and 31 December 2025, with full text retrievable and explicit reference to the exposure and outcome constructs. Exclusions comprised non-English publications, animal/in vivo studies, unpublished or conference-only material, publications outside the date range, case reports/series with very small samples, and records lacking reference to the target constructs.

### 2.3. Information Sources and Search Strategy

We searched PubMed/MEDLINE, Scopus, and ScienceDirect for records indexed from 1 January 2015 to 31 December 2025, restricted to English-language, human studies. Search strategies combined two concept blocks joined by the Boolean operator AND: an exposure block (social isolation, loneliness, and related constructs) and an outcome block (inflammation and associated biomarkers); within each block, synonymous terms were combined using OR. In PubMed/MEDLINE, the exposure block combined the MeSH terms “Social Isolation” and “Loneliness” with title/abstract terms, including social engagement, social participation, social connectedness, and social integration; the outcome block combined the MeSH terms “Inflammation,” “Biomarkers,” and “Inflammation Mediators,” with terms including biomarker*, inflammatory marker*, cytokine*, CRP, interleukin*, IL-6, TNF, and fibrinogen, alongside the MeSH term “C-Reactive Protein.” The query was further restricted using the “humans” MeSH filter, an English-language limit, and a 1 January 2015–31 December 2025 date filter. Equivalent Boolean logic was implemented in Scopus and ScienceDirect, using their own native syntax, with matching year and language filters. Search terms were adapted to each database’s indexing conventions while preserving the conceptual structure, ensuring reproducible, internally consistent retrieval across sources. Complete, verbatim search strings for all three databases are reported in Supplementary Table S1. To assess the sensitivity of the primary search, three additional approaches were applied post hoc: citation searching of the reference lists of all included studies, and a targeted supplementary search (executed in Europe PMC and PsycINFO (via Ovid), October 2026) combining the exposure block with molecular-outcome terms (epigenetic, DNA methylation, proteomic, metabolomic, and suPAR) that were not explicit in the primary outcome block; the verbatim strings are reported in Supplementary Table S1.

### 2.4. Study Selection

**Figure 1:**
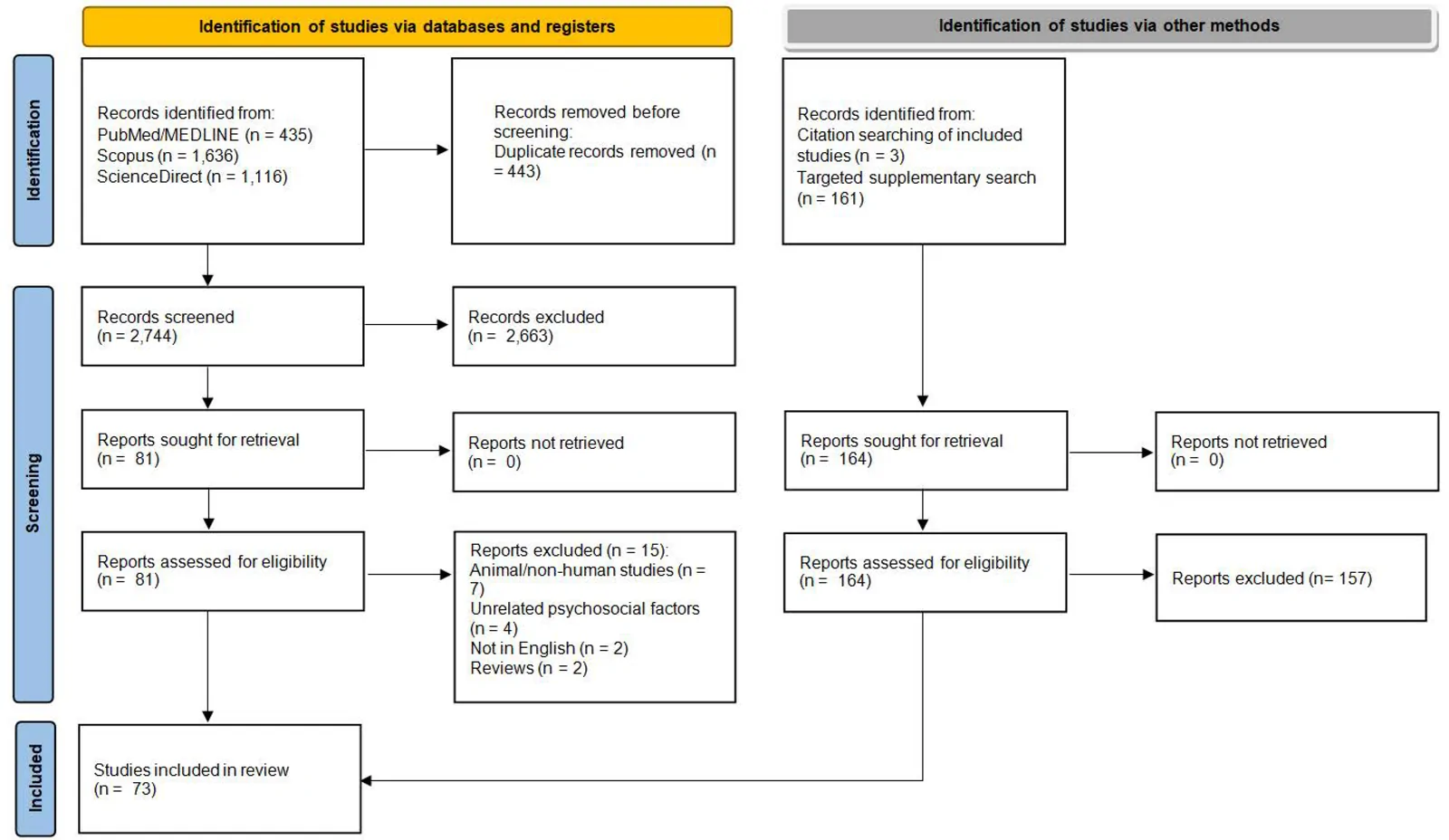
PRISMA 2020 flow diagram showing identification of studies via databases (left arm: PubMed/MEDLINE, Scopus, and ScienceDirect) and via other methods (right arm: citation searching of included studies and a targeted supplementary search executed in October 2026).

Records retrieved from all three databases were merged and deduplicated prior to screening. Titles and abstracts were independently assessed by two reviewers (AB and DS) against the eligibility criteria defined in Section 2.2, with conflicts resolved through discussion and, when unresolved, adjudication by a third author (EKS). Full-text articles meeting initial criteria underwent independent dual review to confirm eligibility, with reasons for exclusion recorded at this stage. This two-stage process is summarized in the PRISMA flow diagram (Figure 1) and detailed with corresponding figures in Section 3.1.

### 2.5. Data Extraction and Thematic Grouping

Two reviewers (AB and DS) independently extracted publication year, country, study design, sample characteristics, the psychosocial construct and its measurement instrument, biomarkers assessed, clinical/functional outcomes, and principal findings—including mediation or moderation analyses—into a structured spreadsheet, resolving discrepancies by discussion. Given substantial heterogeneity across the 73 included studies, findings were organized into five prespecified thematic axes: (1) social isolation and inflammatory biomarkers (structural/objective isolation and markers such as CRP, IL-6, TNF, and fibrinogen); (2) loneliness and inflammatory biomarkers (subjective loneliness and inflammatory or HPA-axis profiles); (3) social isolation, clinical outcomes, and inflammatory mediation (inflammation as a mechanistic pathway to endpoints such as sarcopenia, cardiovascular disease, or diabetic complications); (4) molecular and omics signatures of social disconnection (proteomic, genomic/exome-wide, gene-expression, and epigenetic-ageing outcomes); and (5) physiological, functional, and clinical health outcomes in adults (mental health, cardiovascular risk, and immune function). This framework classified studies by the substantive exposure–outcome relationship addressed, accommodating cross-sectional, longitudinal, and interventional designs alike; studies spanning more than one axis were considered under each applicable theme.

### 2.6. Synthesis Approach

Given marked heterogeneity in exposure definitions, biomarker panels, and outcome measures, synthesis was conducted narratively within each axis rather than through meta-analytic pooling. Where studies examined comparable relationships, we summarized direction and consistency of associations descriptively; quantitative pooling was not undertaken, given this heterogeneity. This review is therefore presented as an evidence map: it charts the distribution, direction, and consistency of evidence across axes and highlights gaps, rather than estimating pooled effects.

## 3. Results

### 3.1. Study Selection Flow

The search across PubMed, Scopus, and ScienceDirect yielded 3187 records, which were reduced to 2744 unique citations after removal of 443 duplicates. The titles and abstracts of these 2744 records were screened for relevance to human populations, psychosocial exposures (social isolation, loneliness, and social connectedness), inflammatory or biological outcomes, and English-language publication; 2663 records were excluded for not meeting these criteria.

The remaining 81 articles underwent full-text assessment, during which 15 were excluded (animal/non-human studies, *n* = 7; unrelated psychosocial exposures, *n* = 4; non-English publications, *n* = 2; review articles without primary data, *n* = 2; these features were not reliably determinable from titles and abstracts alone), leaving 66 database-derived studies. Citation searching of included studies identified three additional studies, and the targeted supplementary search yielded 134 records in Europe PMC and 27 in PsycINFO, of which 114 were excluded at title/abstract screening, and 43 at full-text assessment, leaving four further eligible studies. In total, 73 studies were included in the qualitative synthesis. The full selection process is presented in the PRISMA flow diagram (Figure 1).

### 3.2. Study and Sample Overview

Across the 73 included studies [9,10,11,12,14,15,16,17,18,19,20,21,22,23,24,25,26,27,28,29,30,31,32,33,34,35,36,37,38,39,40,41,42,43,44,45,46,47,48,49,50,51,52,53,54,55,56,57,58,59,60,61,62,63,64,65,66,67,68,69,70,71,72,73,74,75,76,77,78,79,80,81,82], designs spanned cross-sectional observational analyses, longitudinal cohort studies, case–control investigations, randomized controlled trials, single-case experimental designs, and large-scale proteomic or exome-wide genomic analyses. Cross-sectional studies made up the largest subset, with longitudinal cohorts and omics-based studies concentrated in axes addressing clinical outcomes and broader biological signatures; detailed design counts, by axis and study, are reported in Table 1, the Supplementary Data, and Supplementary Tables S2–S4.

Studies were conducted in North America, Europe, and Asia, including works from the United States, the United Kingdom, Germany, China, Hong Kong, Turkey, and Japan. Populations encompassed community-dwelling older adults, general and midlife adults, adolescents and young adults, people with diabetes, people living with HIV aged ≥50 years, individuals at clinical high risk for psychosis, breast cancer survivors, and COVID-19 survivors in both adolescent and older-adult cohorts. Several large cohorts drew on nationally representative samples (e.g., NHANES, English Longitudinal Study of Ageing, National Health and Aging Trends Study, and UK Biobank), while others focused on vulnerable clinical groups such as hospitalized schizophrenia patients and ageing people with HIV.

### 3.3. Axis 1: Social Isolation and Inflammatory Biomarkers

**Figure 2:**
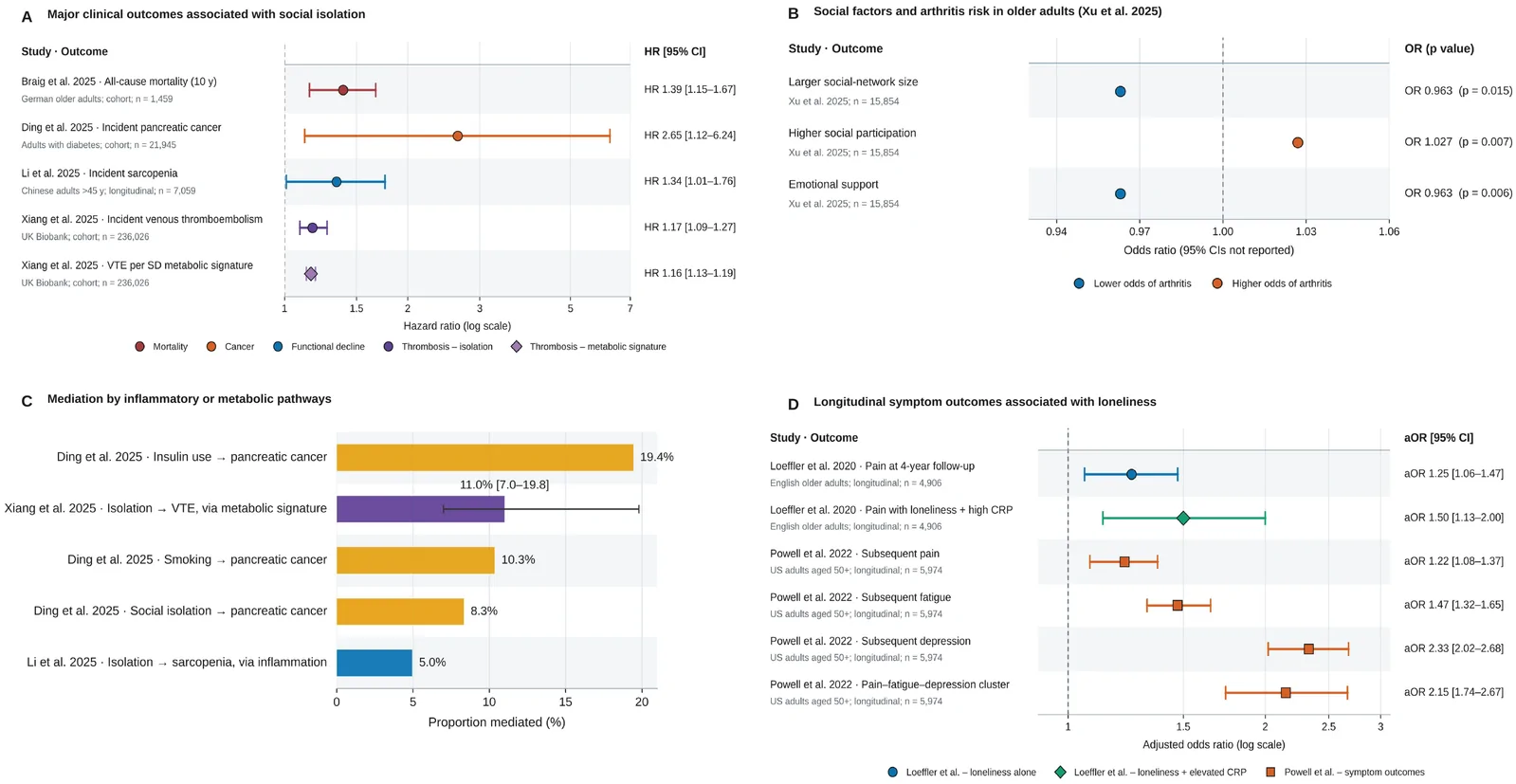
Associations between social isolation, loneliness, and biological/clinical outcomes. (**A**) Major clinical outcomes (mortality, cancer, sarcopenia, and VTE) associated with social isolation; markers show HR/OR with 95% CI, colored by outcome domain [35,56,58,71]. (**B**) Social factors and arthritis risk in 15,854 Chinese older adults [59]; CIs not reported, so only ORs and *p*-values are shown. (**C**) Proportion of exposure–outcome associations mediated by inflammatory/metabolic pathways across three studies; error bars shown where 95% CIs were reported [56,58,71]. (**D**) Longitudinal associations between loneliness and subsequent pain, fatigue, depression, and a symptom cluster, with a CRP-stratified subgroup (diamond) [73,74]. Estimates are study-specific and not pooled; dashed lines mark the null value (HR/OR/aOR = 1.0). CI, confidence interval; CRP, C-reactive protein; HR, hazard ratio; OR, odds ratio; VTE, venous thromboembolism.

**Figure 3:**
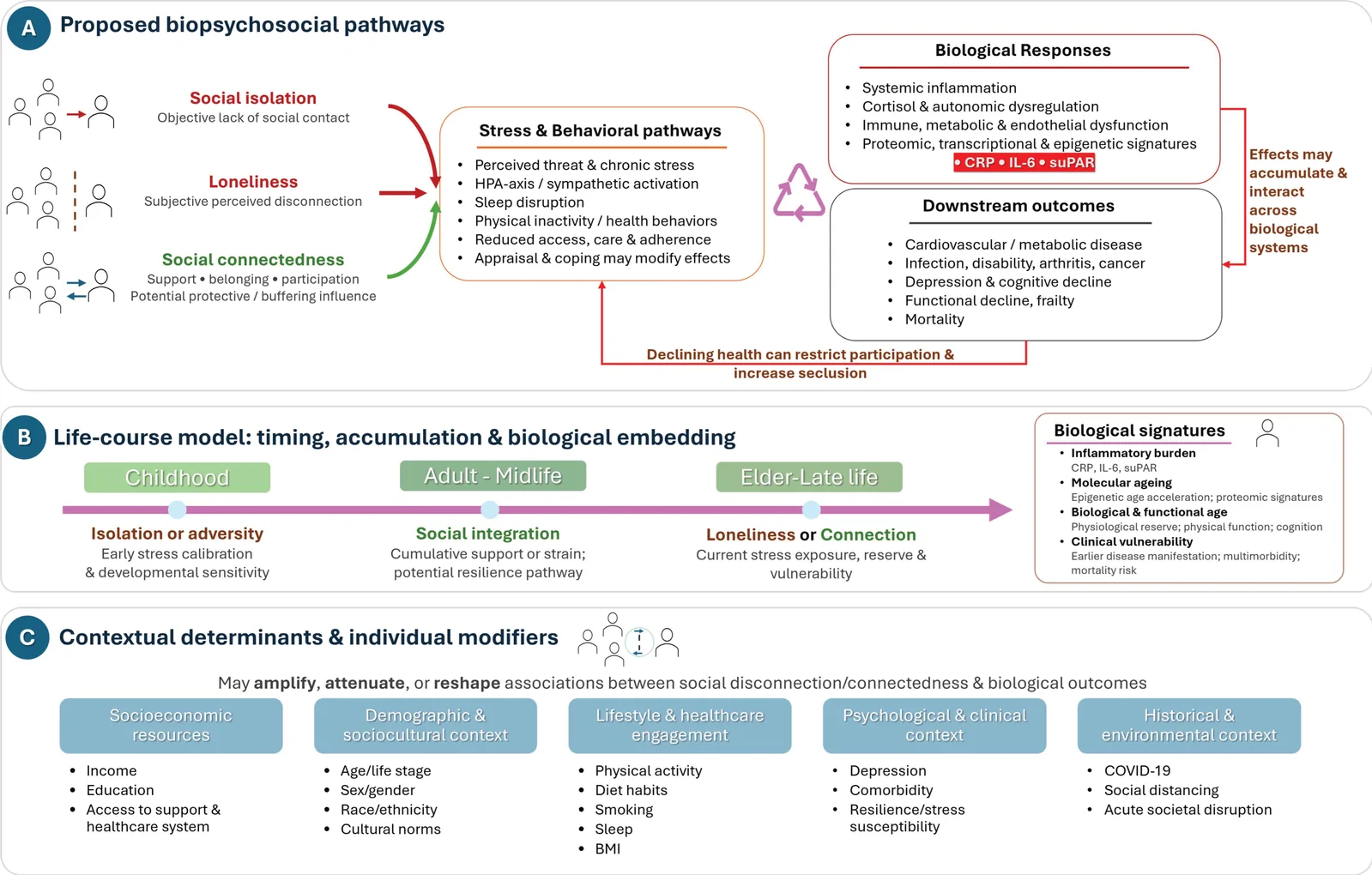
Author-proposed conceptual framework and life-course model. (**A**) Proposed pathways linking social isolation, loneliness, and social connectedness to stress-system activation, inflammatory biomarkers, and downstream clinical outcomes. (**B**) Life-course model showing how childhood isolation or adversity, midlife social integration, and late-life loneliness may shape biomarker profiles, including CRP, IL-6, suPAR, and epigenetic ageing, as well as cognition. (**C**) Contextual modifiers, including poverty, gender, cultural norms, and the COVID-19 pandemic, that may influence both social exposures and biological responses. CRP, C-reactive protein; IL-6, interleukin-6; suPAR, soluble urokinase plasminogen activator receptor.

Axis 1 included studies in which structural or objective isolation—living alone, low Social Network Index scores, reduced network diversity, infrequent social participation—was related to inflammatory and stress-related biomarkers such as C-reactive protein (CRP), interleukin-6 (IL-6), fibrinogen, and cortisol (Table 1, Supplementary Tables S2 and S4, and Figure 2 and Figure 3). In 3360 community-dwelling women aged 40–89 years from NHANES, social isolation, as defined by Social Network Index categories, was associated with higher mean fibrinogen but not with CRP; obesity, smoking, low physical activity, and higher education, rather than isolation itself, independently predicted cancer mortality, suggesting that fibrinogen may mark an inflammatory pathway, while mortality risk is largely driven by behavioral and socioeconomic factors [68].

In 314 adults from the MIDUS Refresher biomarker and diary substudies, living alone was associated with a flatter diurnal cortisol slope and higher CRP, while loneliness was independently associated with higher IL-6, indicating distinct endocrine and immune correlates of structural isolation and subjective disconnection [16]. In the National Health and Aging Trends Study, extreme social isolation in 4648 older adults was linked to higher IL-6 and CRP, providing convergent evidence that severe isolation corresponds to low-grade systemic inflammation, although specific adjusted effect estimates were modest [18].

Newer inflammatory markers extended these findings. In three longitudinal cohorts (TRIAGE, *n* = 6144; Dunedin, *n* = 881; and E-Risk, *n* = 1448), living alone was associated with higher soluble urokinase plasminogen activator receptor (suPAR)—a stable marker of chronic inflammation—but not with CRP or IL-6, and childhood social isolation predicted higher adult suPAR after covariate adjustment [10].

COVID-19 studies showed how acute social distancing modifies inflammatory profiles. In 60 hospitalized schizophrenia patients isolated due to COVID-19, social isolation was associated with higher CRP and more severe psychological distress compared with non-isolated controls [20]. In 624 Japanese adults, combined social isolation and loneliness during the pandemic were associated with elevated neutrophil-to-lymphocyte ratio and CRP, supporting a synergistic impact of objective and perceived disconnection on chronic inflammation [21]. Among older adults undergoing prolonged distancing, repeated salivary sampling in a small longitudinal cohort showed dynamic shifts in cytokine ratios, underscoring immune sensitivity to sustained isolation [19].

Clinical cohorts yielded nuanced findings. In 156 ageing individuals with and without HIV, inadequate social support was associated with higher plasma and cerebrospinal fluid inflammatory markers, suggesting that support deficits exacerbate neuroimmune activation [34]. In contrast, a study of 199 people living with HIV aged ≥50 years reported no independent association between loneliness or social isolation and inflammatory markers after covariate adjustment, indicating heterogeneity by population and disease context [22].

Overall, Axis 1 shows that objective isolation is frequently—though not uniformly—linked to dysregulated inflammatory and endocrine profiles (higher fibrinogen, IL-6, CRP, and suPAR, and flatter cortisol slopes), with several cohorts reporting null associations after covariate adjustment [22,68]. Translation into clinical outcomes such as cancer mortality is often attenuated when obesity, smoking, physical activity, and socioeconomic variables are included, highlighting the embeddedness of isolation within broader risk constellations [16,18,68].

### 3.4. Axis 2: Loneliness and Inflammatory Biomarkers

Axis 2 focused on loneliness—assessed via trait scales and day-to-day ratings—in relation to inflammatory markers, stress physiology, and functional ageing. In older adults experiencing loneliness during the COVID-19 pandemic in Hong Kong, six-month follow-up showed that increases in perceived social support and mindfulness were associated with reduced loneliness, steeper diurnal cortisol slopes, and lower CRP, with loneliness acting as a mediator between psychosocial change and physiological improvement [54].

Daily-process data from 1008 adults in the MIDUS cohort demonstrated that day-to-day loneliness, rather than trait social connection, predicted higher IL-6, reduced insulin-like growth factor-1, weaker grip strength, slower gait, and greater functional limitations after adjustment for age, comorbidities, body mass index, and depressive symptoms [51]. In 822 adults from the MIDUS Biomarker Project, loneliness contributed to IL-6 variance beyond age, sex, and adiposity; cross-sectional serial mediation models were consistent with a pattern in which higher loneliness co-varied with higher body mass index and IL-6 and slower gait speed; because the data are cross-sectional, directionality cannot be inferred [45].

Consistent with this, in 222 community-dwelling older adults, both trait loneliness and momentary (ecological momentary assessment) loneliness were associated with higher CRP (β = 0.16 and *p* = 0.02; and β = 0.15 and *p* = 0.03, respectively), whereas no associations were observed for a broader cytokine panel, suggesting that CRP may be particularly sensitive to day-to-day loneliness fluctuations [9].

Cross-sectional work in middle-aged adults showed additive and interacting effects. In 10,912 older adults, loneliness and obesity, together, were associated with higher hsCRP, suggesting that psychological and metabolic risks combine to amplify systemic inflammation [44]. Loneliness in middle-aged adults in MIDUS was positively related to CRP, IL-6, and fibrinogen, but these associations weakened when age, gender, and social support were considered, indicating partial confounding or moderation by demographic and support factors [37,49]. In the Multi-Ethnic Study of Atherosclerosis, loneliness showed small positive associations with inflammatory markers, with variation across baseline health and ethnicity [50].

Loneliness also interacted with stress and clinical comorbidity. In people with type 2 diabetes, loneliness was associated with altered inflammatory and neuroendocrine responses to acute stress, including reduced cortisol and MCP-1 reactivity, pointing to a blunted stress-response pattern that may still be maladaptive [47]. In Taiwanese middle-aged and older adults, loneliness and isolated living were linked to higher stress-related biomarkers and greater depressive symptoms and disability, with gender differences in biomarker profiles [48].

Mechanistic studies extended these associations to gene expression and epigenetic ageing. Loneliness measured five years ante mortem was associated with differential expression of disease-related genes in dorsolateral prefrontal cortex, implicating immune and neurodegenerative pathways [46]. In 1814 older adults, longitudinal loneliness trajectories were associated with poorer cognitive function, mediated by DNA methylation age-acceleration measures such as GrimAge Accel [11]. Experimental work in adolescents recovering from COVID-19 showed strong links between loneliness, internalizing symptoms, and inflammatory markers, though CRP did not independently predict depressive outcomes, suggesting psychosocial mechanisms may be prominent in younger groups [77].

Altogether, Axis 2 indicates that loneliness is frequently associated with higher CRP and IL-6, altered cortisol rhythms, accelerated epigenetic ageing, and functional decline, with some cohorts showing robust associations after adjustment for depression and multimorbidity, and others demonstrating attenuation when adiposity or affective symptoms are included (Table 2) [11,45,54].

**Table 2:** Associations between social variables and key biomarkers (Axes 1–2).

Exposure Category	Study (Author/Year)	Biomarker(s)	Statistical Metric	Effect Estimate(s) (with 95% CI/p, When Reported)	Direction/Consistency	Key Covariates Considered
Structural isolation (degree of isolation via SNI)	Koh-Bell et al., 2021 [68]	Fibrinogen	Group comparison; *p*-value	Mean fibrinogen ↑ with higher isolation; *p* = 0.003; exact group means NR	Consistent ↑ across isolation categories	Income, education, physical activity, obesity, smoking, age
Structural isolation	Koh-Bell et al., 2021 [68]	CRP	Group comparison; *p*-value	No association between CRP and isolation; *p* = 0.52	Null in this cohort	Same as above
Structural isolation (living alone)	Zilioli et al., 2021 [16]	CRP	Regression (β, NR); *p*-values NR	Living alone associated with higher CRP vs. not living alone; exact β NR	↑ CRP with living alone	Age, sex, loneliness, social contact frequency, health risk factors
Structural isolation (living alone)	Zilioli et al., 2021 [16]	Cortisol (diurnal slope)	Regression (β, NR)	Living alone associated with flatter diurnal cortisol slope (reduced decline over day); numeric β NR	Adverse endocrine pattern	Same as above
Loneliness (trait)	Zilioli et al., 2021 [16]	IL-6	Regression (β, NR)	Higher loneliness associated with higher IL-6, independent of isolation; numeric β NR	↑ IL-6 with loneliness	Living status, social contacts, age, sex, health risk factors
Loneliness (trait)	MacAulay et al., 2024 [45]	IL-6	Regression; serial mediation	Age, sex, BMI, waist–hip ratio, and loneliness, but not multimorbidity/depression, contributed to IL-6 variance; cross-sectional serial-mediation pattern involving BMI and IL-6 in relation to gait speed (directionality not testable); exact βs NR	Loneliness indirectly ↑ IL-6 and slows gait	Age, sex, BMI, waist–hip ratio, multimorbidity, depression history/symptoms, number of friends
Loneliness (trait)	Vasan et al., 2024 [76]	Pulse transit time (arterial stiffness)	Linear regression coefficient	β ≈ −0.70, *p* = 0.002 (higher loneliness → shorter pulse transit time, ↑ stiffness)	Statistically significant adverse association in a small (*n* = 66) cross-sectional sample	Depression, social anxiety, cardiovascular covariates
Depression (symptom severity)	Vasan et al., 2024 [76]	Endothelial function index	Linear regression coefficient	β ≈ 0.43, *p* = 0.04 (higher depression → increased endothelial dysfunction)	Adverse association; distinct from loneliness	Same as above
Social connectedness (positive interactions with close others)	Bajaj et al., 2016 [15]	IL-6	Regression (β, NR)	Higher relative frequency of positive interactions with spouse, friends, family, coworkers → lower IL-6 in older adults; exact βs NR	Consistent ↓ IL-6 with more positive close interactions	Age, sex, race, education, BMI, smoking, alcohol
Global social support and integration	Bajaj et al., 2016 [15]	IL-6, CRP	Regression	Global ISEL/SNI measures not consistently associated with IL-6 or CRP; numeric estimates NR	Mostly null	Same as above
Changes in loneliness; social support; mindfulness	Ho et al., 2026 [54]	Cortisol slope, CRP	Path analysis (standardized coefficients NR)	Greater improvements in social relationships and mindfulness → ↓ loneliness; ↓ loneliness → steeper cortisol slope and ↓ CRP; specific path coefficients NR	Beneficial endocrine and inflammatory changes with improved social relationships/mindfulness	Baseline psychosocial levels, repeat measures over time

Abbreviations: CRP, C-reactive protein; IL-6, interleukin-6; SNI, Social Network Index; BMI, body mass index; ISEL, Interpersonal Support Evaluation List.

### 3.5. Axis 3: Social Isolation, Clinical Outcomes, and Inflammatory Mediation

Axis 3 examined social isolation as a predictor of clinical outcomes—sarcopenia, arthritis, venous thromboembolism, pancreatic cancer, cardiovascular indicators, sleep quality, and mortality—and the extent to which inflammatory or metabolomic markers mediated these associations. In 7059 Chinese adults of middle and older age, social isolation, characterized by living alone and being unmarried, was associated with incident sarcopenia over four years, with systemic inflammation accounting for about 5% of the total effect, indicating a modest but significant mediating role of inflammatory pathways [58].

In 15,854 older Chinese adults, larger social networks and emotional support were associated with lower arthritis risk, whereas higher social participation was paradoxically linked to increased arthritis risk; this adverse association was mitigated when emotional support was strong, suggesting that the quality and supportiveness of participation are critical [59]. A longitudinal study of 4036 older adults showed that social isolation accelerated biological ageing via behavioral and psychological mechanisms, including physical inactivity, smoking, poor sleep, and depressive symptoms, which likely interact with inflammatory processes [60].

UK Biobank studies provided high-dimensional biological context. In 21,945 adults with diabetes, social isolation was associated with substantially increased pancreatic cancer risk (hazard ratio, 2.65; 95% CI, 1.12–6.24), with inflammatory markers explaining part of the association [71]. In 236,026 participants free of venous thromboembolism at baseline, social isolation was associated with incident venous thromboembolism (HR, 1.17; 95% CI, 1.09–1.27), and an isolation-associated plasma metabolomic signature predicted incident deep-vein thrombosis and pulmonary embolism (HR, 1.16 per SD; 95% CI, 1.13–1.19); mediation analysis indicated that this signature accounted for 11.0% (95% CI, 7.0–19.8) of the isolation–VTE association, supporting a metabolic pathway from social disconnection to thrombotic risk [56].

Other longitudinal work linked social engagement to early cardiovascular and stress outcomes. In young adults from the CARDIA study, sleep disturbance predicted higher inflammation over time, with social integration buffering this risk and social isolation amplifying it [65]. Increased social integration after relocation to a new city predicted lower systolic blood pressure in adults, illustrating cardiovascular benefits of rebuilding social networks [66]. A large cohort analysis showed that considerable isolation and loneliness were independent predictors of cardiovascular disease, partly mediated by low-grade inflammation [64].

Collectively, Axis 3 supports social isolation as a risk factor for functional decline, musculoskeletal disease, cardiometabolic events, and mortality, with inflammatory and metabolomic markers explaining a modest but non-trivial fraction of this excess risk (approximately 5–19% of the total effect when formally quantified) [56,58,71].

### 3.6. Axis 4: Molecular and Omics Signatures of Social Disconnection

Axis 4 integrated studies examining molecular and omics signatures—proteomic, genomic, gene-expression, and epigenetic—of social isolation, loneliness, and social connectedness. In 42,062 UK Biobank participants, plasma proteomic signatures of social isolation and loneliness involved inflammatory, antiviral, and complement pathways; more than half of the associated proteins were prospectively linked to cardiovascular disease, type 2 diabetes, stroke, and mortality over 14 years, and Mendelian randomization supported causal involvement of five proteins (GFRA1, ADM, FABP4, TNFRSF10A, and ASGR1), two of which (ADM and ASGR1) were further supported by colocalization [26]. An independent plasma proteome analysis in the English Longitudinal Study of Ageing (*n* = 3262) identified 11 proteins associated with social isolation after minimal adjustment, of which three (TNFRSF10A, TNFRSF11A, and HAOX1) remained significant after full adjustment; Mendelian randomization supported causal effects of lower in-person social contact on higher TNFRSF10A/TRAIL-R2, TNFRSF11A, and KIM1, and lower NEFL [80].

Exome-wide analyses in over 336,000 UK Biobank participants identified 13 risk genes for loneliness and six for isolation, with 47 gene–brain structure associations and phenome-wide links to blood biomarkers and cognitive measures, indicating that social disconnection is embedded in neuroanatomical and systemic biological variation [17]. Gene-expression studies showed that loneliness was related to up-regulation of inflammatory gene sets and down-regulation of antiviral profiles, reinforcing psychoneuroimmunological models [36,46].

Epigenetic-ageing studies formed a coherent sub-theme within Axis 4. In 1310 MIDUS participants, loneliness was weakly associated with epigenetic age acceleration across Horvath, DunedinPACE, and GrimAge estimators (β ≈ 0.08–0.11), and a loneliness × DunedinPACE interaction predicted chronic-condition burden (β = 0.09 and *p* = 0.009) [12]. In 4018 Health and Retirement Study participants, loneliness predicted GrimAge acceleration, which partly mediated the association between loneliness and multimorbidity (*p* = 0.003) [79]. In 694 older Black Americans, increases in loneliness and BDNF methylation predicted accelerated blood-based cortical ageing, robust to adjustment for DunedinPACE and Pace of Aging [81]; and in 685 late-middle-aged Black adults, loneliness combined with lower income predicted increased DunedinPACE over 11 years, robust to cell-type composition, smoking, and alcohol use [82].

### 3.7. Axis 5: Physiological, Functional, and Clinical Outcomes in Adults

Relationship quality emerged as a strong protective factor. In 1829 Black women, very happy romantic relationships and close maternal relationships were associated with lower odds of being in moderate to very high hsCRP cardiovascular risk categories, whereas overall social integration scores were weaker predictors, underscoring the importance of relationship quality over mere network size [33]. In two ecological momentary assessment cohorts, a higher proportion of positive daily interactions with close others (spouse, family, friends, and co-workers) was associated with lower IL-6 in older adults, while global support and integration measures showed mixed associations, again emphasizing interaction quality [15]. Cross-cultural work showed that positive affect and social connectedness jointly predicted healthier lipid profiles in Japan and the United States [27].

Taken together, Axis 5 indicates that, in adult populations, higher social integration and supportive, high-quality relationships are associated with more favorable inflammatory, lipid, and functional profiles, whereas loneliness is linked to less favorable cardiovascular and symptom profiles (Table 3). These physiological and clinical correlates are conceptually distinct from the molecular signatures of Axis 4, although both levels likely interact across the life course [15,27,33].

**Table 3:** Clinical and biological outcomes linked to social isolation/loneliness and potential inflammatory or biomarker mediation (Axes 3–5).

Outcome Domain	Study (Author/Year)	Social Exposure(s)	Biomarker(s)/Biological Marker(s)	Statistical Metric	Effect Estimate(s) (HR/β/p, where Available)	Mediation/Moderation Evidence	Study Type
Functional ageing (gait speed)	MacAulay et al., 2024 [45]	Loneliness; BMI	IL-6	Serial mediation model	Loneliness contributed to IL-6 variance; cross-sectional serial-mediation pattern across loneliness, BMI, IL-6, and walking time (directionality not testable); individual coefficients NR	Cross-sectional serial mediation via BMI and IL-6; indirect paths are statistical associations, not evidence of temporal ordering	Cross-sectional observational
Functional ageing (cortisol/CRP)	Ho et al., 2026 [54]	Loneliness; social network; perceived support; mindfulness	Diurnal cortisol slope; CRP	Path analysis	Small–moderate decreases in loneliness and pre-lunch cortisol; greater improvements in social relationships and mindfulness indirectly associated with greater reductions in CRP via decreased loneliness; exact coefficients NR	Loneliness acts as mediator between psychosocial improvements and cortisol/CRP changes	Prospective observational (baseline, 6-month follow-up)
Early cardiovascular disease markers	Vasan et al., 2024 [76]	Loneliness; depressive symptoms	Pulse transit time; endothelial function indices	Linear regression	Loneliness: β ≈ −0.70, *p* = 0.002 (↑ loneliness → ↑ arterial stiffness); depression: β ≈ 0.43, *p* = 0.04 (↑ depression → ↑ endothelial dysfunction)	Distinct mechanisms for loneliness vs. depression; no formal mediation by inflammation tested	Cross-sectional observational
Venous thromboembolism (VTE)	Xiang et al., 2025 [56]	Social isolation composite (friend/family visits, group activities, living alone)	Plasma metabolomic signature	Cox regression; mediation analysis	Social isolation associated with incident VTE: HR 1.17 (95% CI 1.09–1.27); metabolomic signature: HR 1.16 per SD (95% CI 1.13–1.19); mediation proportion 11.0% (95% CI 7.0–19.8)	Metabolic signature mediated 11.0% (95% CI 7.0–19.8) of the social isolation–VTE association	Prospective cohort (UK Biobank)
Cardiometabolic disease, stroke, mortality	Shen et al., 2025 [26]	Loneliness; social isolation	2920 plasma proteins; MR-identified proteins (GFRA1, ADM, FABP4, TNFRSF10A, ASGR1)	Proteome-wide association; Cox regression; MR and colocalization	>50% loneliness/isolation-linked proteins prospectively associated with CVD, T2D, stroke, mortality over ≈14 years; MR indicates causal pathways from loneliness to five proteins; ADM and ASGR1 colocalize with genetic signals; specific HRs for CVD/stroke/mortality NR	MR-identified proteins partly mediate relationships between loneliness and cardiometabolic outcomes; mechanistic support for causality	Prospective cohort with genetic causal inference
Cancer mortality	Koh-Bell et al., 2021 [68]	Social isolation (SNI categories)	Fibrinogen, CRP	Cox regression	Obesity HR = 1.56 (95% CI 1.11–2.18); higher education HR = 1.36 (95% CI 1.01–1.83); smoking HR = 4.42 (95% CI 2.84–6.88); high physical activity HR = 0.67 (95% CI 0.51–0.87) for cancer mortality; social isolation not significantly associated with cancer mortality (*p* = 0.54); elevated fibrinogen associated with higher cancer mortality but not significantly (*p* = 0.08)	Fibrinogen may reflect inflammatory pathway to cancer mortality, but social isolation effects largely explained by lifestyle and socioeconomic factors	Cross-sectional with mortality follow-up (NHANES women)
Multi-system morbidity/mortality and brain phenotypes	Shen et al., 2025 [26]; Wang et al., 2025 [17]	Loneliness; social isolation	Plasma proteins and metabolite profiles (Shen et al.); risk genes and protein-coding variants (Wang et al.)	MR, phenome-wide association; brain-wide association	Shen et al.: MR suggests causal links from loneliness to specific proteins that are associated with cardiometabolic events and mortality; Wang et al.: 13 risk genes for loneliness, 6 for isolation; 47 gene–brain structure associations; effect sizes for individual variants NR	Proteomic and genetic markers indicate biological pathways through which social disconnection influences morbidity and mortality	Prospective cohort (UK Biobank proteomics); exome-wide genetic study
Mortality and functional health in older adults	Braig et al., 2025 [35]	Social isolation (family, friends, overall); loneliness	hs-CRP; GDF-15; NT-proBNP; hs-cTnT; hs-cTnI; gait speed; handgrip strength	Linear regression; Cox regression	High SI overall associated with increased 10-year mortality, HR 1.39 (95% CI 1.15–1.67), Model 2; SI from friends linked to adverse hs-CRP, GDF-15, hs-cTnT at follow-up (Model 1); SI from family associated with NT-proBNP (Model 2); high SI and moderate-to-severe loneliness associated with lower gait speed; loneliness linked to hs-CRP at baseline but was a weaker predictor than SI overall	SI (particularly from friends) more consistently linked to adverse biomarker profiles than loneliness; functional parameters and biomarkers analyzed in parallel rather than as formal mediators of the SI–mortality association	Prospective population-based cohort study (ActiFE Ulm, Germany); community-dwelling adults 65+; N = 1459 baseline, N = 819 at 3-year follow-up; 10-year mortality follow-up
Stress, depression, sleep outcomes	Patapoff et al., 2024 [53]	Loneliness; chronic stress; self-compassion	BP, inflammation markers, sleep actigraphy	Repeated-measures ANOVA	Stress *p* < 0.001 (η^2^ = 0.15); depression *p* = 0.02 (η^2^ = 0.08); loneliness *p* = 0.003 (η^2^ = 0.18); self-compassion *p* = 0.01 (η^2^ = 0.13) from control to post-intervention; some improvements in BP, inflammatory markers and sleep quality in subsample; exact biomarker effect sizes NR	Suggests that psychosocial intervention can influence both psychosocial and physiological outcomes in older adults	Single-case experimental design intervention (multiple phase changes)

Abbreviations: BMI, body mass index; CRP, C-reactive protein; IL-6, interleukin-6; CVD, cardiovascular disease; T2D, type 2 diabetes; VTE, venous thromboembolism; MR, Mendelian randomization; BP, blood pressure; HR, hazard ratio; NHANES, National Health and Nutrition Examination Survey; η^2^, partial eta-squared.

### 3.8. Interventional and Experimental Studies

Interventional and experimental studies, though fewer in number than observational designs, provide proof of concept that modifying social experiences or targeting resilience can influence inflammatory and stress biology. In a single-case experimental design with 43 older adults, a remotely delivered resilience and self-compassion program produced substantial reductions in stress (partial η^2^ ≈ 0.15), depression (≈0.08), and loneliness (≈0.18), and increases in self-compassion (≈0.13); exploratory analyses indicated improvements in blood pressure, inflammatory markers, and sleep quality in a subsample, but biomarker effect sizes were not fully quantified [53].

In a randomized controlled trial of 59 community-dwelling older adults, horticultural therapy increased social connectedness, and mediation analyses showed that gains in social connection partially explained six-month reductions in IL-6, suggesting that structured programs fostering meaningful interaction can alter inflammatory profiles [29]. A double-blind, placebo-controlled endotoxin challenge in 155 healthy adults demonstrated that individuals with higher trait sensitivity to social disconnection—composite of loneliness, anxious attachment, fear of negative evaluation, and rejection sensitivity—mounted larger TNF-α and IL-6 responses and exhibited a conserved transcriptional response to adversity, characterized by up-regulated pro-inflammatory and down-regulated antiviral transcription factors [42].

Longer-term stress-related intervention data showed bidirectional links between inflammation and social behavior: in 120 mothers of children with cancer, stress-induced IL-6 elevations predicted subsequent reductions in social engagement over one year, pointing to feedback loops between inflammatory activation and social withdrawal [40]; this bidirectionality is considered further in the Discussion.

Overall, these interventional and experimental studies suggest that enhancing social connection, resilience, and self-compassion, and reducing sensitivity to social disconnection, can modulate inflammatory responses and stress physiology, but sample sizes are small, follow-up periods are short, and long-term clinical outcomes remain largely untested; current evidence should therefore be viewed as preliminary and hypothesis-generating rather than definitive [29,42,53].

## 4. Discussion

Social isolation and loneliness were consistently, though not uniformly, linked to adverse inflammatory and stress biomarker profiles, and to worse functional and clinical outcomes, while social connectedness emerged as broadly protective across multiple biological systems [16,26,33,68]. Objective isolation and time-varying loneliness tracked with higher CRP, IL 6, and fibrinogen; flatter cortisol rhythms; and greater risk of sarcopenia, venous thromboembolism, pancreatic cancer, cardiovascular disease, and mortality, whereas stronger social integration and positive relationship quality aligned with more favorable inflammatory and functional profiles [15,35,56,58,64]. Evidence is most robust in large cohorts of older and middle-aged adults; findings in adolescents, young adults, clinical high-risk psychiatric groups, and COVID 19 survivors are promising but still preliminary [54,72,76,77]. As an evidence map, this review charts where evidence is concentrated, consistent, or sparse rather than estimating pooled effects.

These patterns mirror results from major international cohorts—MIDUS, the English Longitudinal Study of Ageing, the Multi Ethnic Study of Atherosclerosis, and UK Biobank—where loneliness and isolation predicted higher inflammatory scores and poorer functional markers; however, associations often weakened after adjusting for obesity, depression, or support [23,37,44,50]. For instance, isolation lost its independent link to cancer mortality once lifestyle and socioeconomic factors were accounted for in NHANES women [68]. This suggests that social disconnection often marks a broader cluster of behavioral and affective risk rather than acting as an isolated cause. Measurement heterogeneity compounds the challenge: studies combined different loneliness scales, network indices, and ecological assessments with biological endpoints, ranging from single cytokines to full plasma proteomes, limiting direct comparability, and calling for harmonized, multidimensional instruments alongside standardized biomarker panels [15,17,26].

Mechanistically, the findings support a psychoneuroimmunological pathway: chronic disconnection sustains HPA axis and sympathetic activation; alters immune gene expression; and drives pro-inflammatory, proteomic, and ageing-related biomarker shifts (Figure 2) [36,46,54]. Within-study dissociations sharpen this model: structural isolation tracked more closely with CRP and fibrinogen—hepatic acute-phase reactants plausibly influenced through behavioral and metabolic routes—whereas loneliness tracked more closely with IL-6, consistent with threat-related HPA and sympathetic-immune transcriptional programs [16]. This dissociation is hypothesis-generating rather than confirmatory, but it offers a concrete, testable framework for future mechanistic studies. Daily diary data show that transient loneliness precedes IL 6 elevation and functional decline, while interventions boosting perceived support and mindfulness lower cortisol and CRP, reinforcing the biological responsiveness of short-term social experience [45,51,53]. Proteomic and genetic studies deepen this picture: UK Biobank plasma signatures link loneliness to inflammatory, antiviral, and complement pathways, and to cardiometabolic disease risk, with Mendelian randomization supporting causal involvement of a small number of proteins, while exome-wide analyses tie isolation-related genes to brain structure and cognition [17,26]. Life-course data add that childhood isolation predicts midlife retinal and inflammatory changes, and loneliness trajectories in older age correlate with epigenetic ageing (GrimAge acceleration) and cognitive decline, with COVID-19-era studies showing how acute isolation compounds pre-existing stress vulnerability [11,55,57,77]. Structural isolation, perceived support, and network diversity appear to act through partly distinct pathways—loneliness aligning more with vascular stiffness and symptom burden, and network diversity with lipid and inflammatory profiles [69,76].

Clinically, this evidence supports targeted screening for isolation and loneliness in high-risk groups—older adults, people with diabetes or cardiometabolic disease, and patients with severe mental illness or HIV—where social disconnection frequently accompanies poorer biomarker and functional trajectories; however, null findings in some clinical cohorts caution against assuming universality [22,34,61,71]. Brief validated screening tools, paired with referral pathways to social prescribing, could be embedded into geriatric and cardiometabolic care [33,38]. At the individual level, resilience and self-compassion programs have reduced loneliness and stress with exploratory physiological benefit; at the community level, fostering positive, high-quality interactions appears more biologically impactful than simply increasing contact frequency; and at the policy level, age-friendly design and planned social support during crises may lower population-level inflammatory burden [15,38,41,53]. Biomarkers such as CRP, IL 6, and suPAR can usefully track intervention effects, but given their sensitivity to diet, medication, and comorbidity, psychosocial and functional outcomes should remain the primary clinical focus to avoid overmedicalizing social experience [26,36].

This synthesis carries several important limitations. The review protocol was not registered in advance (e.g., no PROSPERO registration), thus raising the possibility of unreported protocol deviations and limiting transparency around pre-specified outcomes and analyses. Most included studies were cross-sectional or short-term, restricting causal and directional inference—and reverse causation remains plausible, as inflammatory activation can itself reduce social engagement [40,41]—while psychosocial exposures relied heavily on self-report with inconsistent use of objective or repeated measures [44,49]. Formal risk-of-bias and quality appraisal tools (e.g., ROBINS-I and Newcastle–Ottawa) were not systematically applied across all designs, and heterogeneity in biomarker assays, timing of sample collection, and laboratory protocols across cohorts was not harmonized, further limiting comparability. Publication and selective-outcome-reporting bias cannot be excluded, as null or small associations may be underrepresented, and the search was restricted to English-language records and three databases published from 2015 to 2025, potentially missing relevant older, non-English or grey literature. The primary database search did not explicitly include molecular-outcome terms (e.g., epigenetic, proteomic, and metabolomic); citation searching and a targeted supplementary search were therefore added post hoc, yielding seven additional included studies. Residual confounding by socioeconomic status, comorbidity, and health behaviors remains likely despite adjustment in some cohorts, and cultural, pandemic-related, and demographic differences across studies limit generalizability, with several samples skewed toward women or specific populations [21,33,68]. Sample sizes and statistical power varied widely, from single-case designs to biobank-scale cohorts, complicating direct synthesis, and formal meta-analytic pooling was not feasible given inconsistent effect metrics (odds ratios, betas, and hazard ratios) and exposure definitions. Intervention studies were largely proof-of-concept types, with small samples, short follow-up, and exploratory biomarker outcomes, so while they support feasibility and biological plausibility, larger and more rigorous trials with harmonized measures are needed to confirm clinically meaningful effects [29,42,53].

## 5. Conclusions

This review indicates that social isolation and loneliness are biologically meaningful exposures, associated with altered inflammatory, endocrine, and proteomic profiles, and with downstream functional and clinical risk, while social connectedness appears to confer a measure of protection across several physiological systems. These associations, however, are heterogeneous in magnitude, frequently attenuated by adjustment for lifestyle and mental-health factors, and derived predominantly from observational designs that cannot establish causality. The evidence should therefore be read as supportive of a plausible psychoneuroimmunological pathway linking social experience to biology, not as proof of a deterministic biological consequence of loneliness or isolation. The recurring within-study dissociation—structural isolation tracking with CRP and fibrinogen, and loneliness with IL-6—offers a concrete, testable hypothesis for future mechanistic work.

Future progress requires three concrete steps: prospective, adequately powered cohorts with repeated psychosocial and biomarker assessment to clarify temporality; harmonized measurement of loneliness, isolation, and inflammatory markers to enable pooled quantitative synthesis; and adequately powered, longer-duration intervention trials with pre-registered protocols and standardized biomarker panels to test whether reducing loneliness or isolation produces measurable, sustained biological and clinical benefit.

Clinically, the findings support cautious, targeted screening for social disconnection in high-risk groups rather than universal biomarker-based intervention. Positioning social isolation and loneliness as modifiable, assessable determinants of health—alongside, not above, established behavioral and clinical risk factors—offers a realistic and evidence-consistent direction for both research and practice going forward.

## Figures and Tables

**Table 1:** Overview of selected included studies by thematic axis, with design and effect estimates. Studies were selected to represent each thematic axis and the range of designs; the complete study-level extraction for all 73 included studies is provided in the Supplementary Data.

Axis	Study (Author/Year)	Country	Design	Population/Setting	Age Range/Mean	Sample Size	Key Psychosocial Exposure(s)	Primary Biomarker(s)	Primary Outcome(s)	Effect Estimate(s), where Reported	Main Adjustment Variables
1. Social isolation and inflammatory biomarkers	Koh-Bell et al., 2021 [68]	USA	Cross-sectional with mortality follow-up (NHANES)	Community-dwelling women	40–89 years; mean 54	3360	Social Network Index (most/very/somewhat/not isolated)	Fibrinogen, CRP	Cancer mortality	Fibrinogen ↑ with isolation (*p* = 0.003); CRP–isolation null (*p* = 0.52); obesity HR = 1.56 (95% CI 1.11–2.18); smoking HR = 4.42 (95% CI 2.84–6.88); high physical activity HR = 0.67 (95% CI 0.51–0.87) for cancer mortality; isolation not an independent predictor (*p* = 0.54)	Age, income, education, physical activity, obesity, smoking
1. Social isolation and inflammatory biomarkers	Zilioli et al., 2021 [16]	USA	Cross-sectional cohort (MIDUS Refresher)	Adults in biomarker and diary substudies	25–75 years; mean 47.7	314	Living status (alone vs. with others); frequency of social contacts; loneliness	CRP, IL-6, salivary cortisol	Endocrine and immune function (cortisol slope, inflammation)	Living alone → flatter diurnal cortisol slope and ↑ CRP; loneliness → ↑ IL-6 above and beyond isolation	Age, sex, loneliness/social isolation measures, health risk factors
2. Loneliness and inflammatory biomarkers/function	MacAulay et al., 2024 [45]	USA	Cross-sectional	National adult sample	26–78 years; mean 52.5	822	Loneliness; BMI; number of friends	IL-6	Gait speed (walking time)	Serial mediation: loneliness → ↑ BMI → ↑ IL-6 → slower gait; age, sex, BMI, waist–hip ratio and loneliness contributed to IL-6 variance; exact β/indirect effects NR	Age, sex, BMI, waist–hip ratio, multimorbidity, depression history/symptoms, number of friends
2. Loneliness and CVD markers	Vasan et al., 2024 [76]	Australia	Cross-sectional	Young adults	18–35 years	66	Loneliness; depression; social anxiety	Pulse transit time; endothelial function indices	Early cardiovascular disease markers	Loneliness associated with shorter pulse transit time (β = −0.70, *p* = 0.002); depression associated with endothelial dysfunction (β = 0.43, *p* = 0.04)	Depression, social anxiety, standard cardiovascular covariates
2. Loneliness, social connectedness and stress/inflammation	Ho et al., 2026 [54]	Hong Kong, China	Prospective observational (baseline and 6-month follow-up)	Older adults experiencing loneliness during COVID-19	Mean 64.1 years	141	Loneliness; social network; perceived social support; mindfulness	Salivary cortisol (six samples over two days); CRP	Psychosocial and physiological well-being	Improvements in social relationships and mindfulness associated with steeper cortisol slope and ↓ CRP via reduced loneliness; numeric path coefficients NR	Baseline levels of psychosocial variables, age, sex (implicit), repeated measures over time
3. Isolation and metabolomic signature/VTE	Xiang et al., 2025 [56]	China/UK	Prospective cohort (UK Biobank)	Adults free of VTE at baseline	NR; middle-aged adults	236,026	Composite social isolation (friend/family visits, group activities, living alone)	Plasma metabolomic signature	Incident VTE (DVT or pulmonary embolism)	Social isolation associated with incident VTE (HR 1.17, 95% CI 1.09–1.27); isolation-associated metabolomic signature associated with VTE (HR 1.16 per SD, 95% CI 1.13–1.19); the signature mediated 11.0% (95% CI 7.0–19.8) of the isolation–VTE association	Age, sex, metabolomic covariates and VTE risk factors
3–4. Isolation/loneliness and proteomic signatures/cardiometabolic outcomes	Shen et al., 2025 [26]	UK	Prospective cohort with proteomics and MR (UK Biobank)	Adults from 22 centers	40–69 years; mean 56.4	42,062	Social isolation and loneliness constructs	2920 plasma proteins; MR-identified proteins (GFRA1, ADM, FABP4, TNFRSF10A, ASGR1)	CVD, T2D, stroke, mortality	>50% proteins prospectively linked to CVD, T2D, stroke, mortality over 14-year follow-up; MR suggests causal links from loneliness to five proteins; two (ADM, ASGR1) supported by colocalization; proteins partly mediate loneliness–cardiometabolic outcomes; HRs for outcomes NR	Age, sex, traditional cardiometabolic risk factors; genetic instruments in MR
4–5. Social connectedness and inflammation	Bajaj et al., 2016 [15]	USA	Observational EMA cohorts	Healthy community-dwelling adults (PHHP and AHAB-II)	PHHP: 50–70 years; AHAB-II: 30–54 years	306 + 419	Global social support (ISEL); social integration (SNI); proportion of day in positive/negative interactions	IL-6, CRP	Systemic inflammation	Positive interactions with close others (spouse, friends, family, coworkers) associated with lower IL-6 in older adults in fully adjusted models; global support/integration not consistently related; numeric βs NR	Age, sex, race, education, BMI, smoking, alcohol
4–5. Genetic architecture of loneliness/isolation	Wang et al., 2025 [17]	UK	Exome-wide association study	UK Biobank, white-British ancestry	Median age 58.0 years; 46.2% women	336,115 (loneliness); 346,115 (isolation)	Loneliness; social isolation (questionnaire-based)	Protein-coding variants; brain structure phenotypes; blood biomarkers	Psychiatric/physical risk phenotypes, brain volumes, biomarkers	13 risk genes for loneliness and 6 for isolation identified; 47 gene–brain structure associations; phenome-wide associations mainly with blood biomarkers and cognitive measures; effect sizes for specific variants NR	Genetic covariates; multiple testing correction; QC filters
5. Interventions targeting loneliness/stress	Patapoff et al., 2024 [53]	USA	Single-case experimental design (multiple phase change)	Older adults in remote resilience/self-compassion program	Mean 76.9 years	43	Loneliness; stress; self-compassion	Blood pressure; inflammatory markers; sleep actigraphy	Stress, depression, loneliness, physiological correlates	From control to post-intervention (weeks 0–12): stress *p* < 0.001 (η^2^ = 0.15), depression *p* = 0.02 (η^2^ = 0.08), loneliness *p* = 0.003 (η^2^ = 0.18), self-compassion *p* = 0.01 (η^2^ = 0.13); some improvements in BP, inflammation and sleep quality in subsample; exact biomarker effect sizes NR	Time (phase), age, sex; repeated measures ANOVA over 12–18 weeks
1. Social isolation and inflammatory biomarkers	Matthews et al., 2024 [10]	Denmark/UK/USA	Longitudinal cohorts (TRIAGE, Dunedin, E-Risk)	Community and birth-cohort samples	Adults 18–45+ years (varies by cohort)	6144 + 881 + 1448	Living alone; childhood social isolation; loneliness	suPAR, CRP, IL-6	Chronic inflammation (suPAR)	Living alone → higher suPAR, but not CRP or IL-6; childhood isolation → higher adult suPAR after adjustment; loneliness at ages 38/45 → suPAR at 45	Age, sex, socioeconomic and health-behavior covariates
4. Proteomic signatures	Gong et al., 2025 [80]	UK	Prospective cohort with proteomics and MR (ELSA)	Community-dwelling adults aged 50+	Mean 63.4 years; 55% women	3262	Social isolation; loneliness	Plasma proteome (276 proteins)	Protein signatures; MR causal inference	11 proteins associated with isolation after minimal adjustment; 3 (TNFRSF10A, TNFRSF11A, HAOX1) after full adjustment; MR: lower in-person contact causally ↑ TNFRSF10A/TRAIL-R2, TNFRSF11A, KIM1 and ↓ NEFL	Demographic, lifestyle and health covariates; genetic instruments in MR
4. Epigenetic ageing	Freilich et al., 2024 [79]	USA	Longitudinal cohort (HRS)	Older adults	50+ years	4018	Loneliness	GrimAge acceleration (DNA methylation)	Multimorbidity	Loneliness → GrimAge acceleration; epigenetic ageing partly mediated the loneliness–multimorbidity association (*p* = 0.003)	Age, sex, race/ethnicity, health behaviors

Abbreviations: CRP, C-reactive protein; IL-6, interleukin-6; CVD, cardiovascular disease; T2D, type 2 diabetes; EMA, ecological momentary assessment; NHANES, National Health and Nutrition Examination Survey; MIDUS, Midlife in the United States study; VTE, venous thromboembolism; DVT, deep vein thrombosis; MR, Mendelian randomization; BP, blood pressure; ISEL, Interpersonal Support Evaluation List; SNI, Social Network Index; QC, quality control.

## Data Availability

All data used in this work is available online through the included articles. Requests for further information should be directed to the corresponding author (Dimitrios Stylianakis, Email: dimitrios.stylianakis@gmail.com).

## Supplementary Materials

The following supporting information can be downloaded at https://www.mdpi.com/article/10.3390/diseases14100388/s1, Supplementary Data: Study characteristics, data extraction, and notes. Supplementary Tables: Supplementary Table S1. Database-specific search strings. Supplementary Table S2. Detailed study-level data extraction matrix for key included studies. Supplementary Table S3. Study characteristics by population subgroup. Supplementary Table S4. Biomarker panel and analytic methods across studies (including proteomics and genomics). Supplementary Table S5. Preferred Reporting Items for Systematic reviews and Meta-Analyses extension for Scoping Reviews (PRISMA-ScR) Checklist.

## Abbreviations

BMI, body mass index; CRP, C-reactive protein; CVD, cardiovascular disease; DNAm, DNA methylation; EMA, ecological momentary assessment; GrimAge Accel, GrimAge acceleration (epigenetic ageing measure); HPA, hypothalamic–pituitary–adrenal (axis); HR, hazard ratio; hsCRP, high-sensitivity C-reactive protein; IFN, interferon; IGF-1, insulin-like growth factor 1; IL-2, interleukin-2; IL-6, interleukin-6; IL-8, interleukin-8; ISEL, Interpersonal Support Evaluation List; MDD, major depressive disorder; MIDUS, Midlife in the United States (study); MR, Mendelian randomization; NHANES, National Health and Nutrition Examination Survey; NLR, neutrophil-to-lymphocyte ratio; OR, odds ratio; PRISMA, Preferred Reporting Items for Systematic Reviews and Meta-Analyses; ScR, Scoping Review; PROSPERO, International Prospective Register of Systematic Reviews; SNI, Social Network Index; suPAR, soluble urokinase plasminogen activator receptor; T2D, type 2 diabetes; TNF-α, tumor necrosis factor-alpha; TSLP, thymic stromal lymphopoietin; VTE, venous thromboembolism; WBC, white blood cell (count).

## References

[1] Holt-Lunstad J., Smith T.B., Baker M., Harris T., Stephenson D. (2015). Loneliness and social isolation as risk factors for mortality: A meta-analytic review. Perspect. Psychol. Sci..

[2] Valtorta N.K., Kanaan M., Gilbody S., Ronzi S., Hanratty B. (2016). Loneliness and social isolation as risk factors for coronary heart disease and stroke: Systematic review and meta-analysis of longitudinal observational studies. Heart.

[3] Schutter N., Holwerda T.J., Comijs H.C., Stek M.L., Peen J., Dekker J.J.M. (2022). Loneliness, social network size and mortality in older adults: A meta-analysis. Eur. J. Ageing.

[4] Harris E. (2023). Surgeon General Offers Strategy to Tackle Epidemic of Loneliness. JAMA.

[5] Office of the Surgeon General (OSG) (2023). Our Epidemic of Loneliness and Isolation: The U.S. Surgeon General’s Advisory on the Healing Effects of Social Connection and Community.

[6] Warren A. (2025). Loneliness as a driver of allostatic load: Mechanisms linking social disconnection to physiological dysregulation and health disparities. Stress.

[7] Pourriyahi H., Yazdanpanah N., Saghazadeh A., Rezaei N. (2021). Loneliness: An Immunometabolic Syndrome. Int. J. Environ. Res. Public Health.

[8] Smith K.J., Gavey S., Riddell N.E., Kontari P., Victor C. (2020). The association between loneliness, social isolation and inflammation: A systematic review and meta-analysis. Neurosci. Biobehav. Rev..

[9] Van Bogart K., Engeland C.G., Sliwinski M.J., Harrington K.D., Knight E.L., Zhaoyang R., Scott S.B., Graham-Engeland J.E. (2022). The Association Between Loneliness and Inflammation: Findings From an Older Adult Sample. Front. Behav. Neurosci..

[10] Matthews T., Rasmussen L.J.H., Ambler A., Danese A., Eugen-Olsen J., Fancourt D., Fisher H.L., Iversen K.K., Schultz M., Sugden K. (2024). Social isolation, loneliness, and inflammation: A multi-cohort investigation in early and mid-adulthood. Brain Behav. Immun..

[11] Lynch M., Em Arpawong T., Beam C.R. (2023). Associations Between Longitudinal Loneliness, DNA Methylation Age Acceleration, and Cognitive Functioning. J. Gerontol. B Psychol. Sci. Soc. Sci..

[12] Freilich C.D., Markon K.E., Cole S.W., Krueger R.F. (2024). Loneliness, epigenetic age acceleration, and chronic health conditions. Psychol. Aging.

[13] Tricco A.C., Lillie E., Zarin W., O’Brien K.K., Colquhoun H., Levac D., Moher D., Peters M.D.J., Horsley T., Weeks L. (2018). PRISMA Extension for Scoping Reviews (PRISMA-ScR): Checklist and Explanation. Ann. Intern. Med..

[14] Hayakawa K., Kato T.A., Watabe M., Teo A.R., Horikawa H., Kuwano N., Shimokawa N., Sato-Kasai M., Kubo H., Ohgidani M. (2018). Blood biomarkers of Hikikomori, a severe social withdrawal syndrome. Sci. Rep..

[15] Bajaj A., John-Henderson N.A., Cundiff J.M., Marsland A.L., Manuck S.B., Kamarck T.W. (2016). Daily social interactions, close relationships, and systemic inflammation in two samples: Healthy middle-aged and older adults. Brain Behav. Immun..

[16] Zilioli S., Jiang Y. (2021). Endocrine and immunomodulatory effects of social isolation and loneliness across adulthood. Psychoneuroendocrinology.

[17] Wang Y.X., Fei C.J., Shen C., Ou Y.-N., Liu W.-S., Yang L., Wu B.-S., Deng Y.-T., Feng J.-F., Cheng W. (2025). Exome sequencing identifies protein-coding variants associated with loneliness and social isolation. J. Affect. Disord..

[18] Cudjoe T.K.M., Selvakumar S., Chung S.E., Latkin C.A., Roth D.L., Thorpe R.J., Boyd C.M. (2022). Getting under the skin: Social isolation and biological markers in the National Health and Aging Trends Study. J. Am. Geriatr. Soc..

[19] Nery G.B., de Araujo C.A.R., da Silva G.B., Bittar H., Bordallo V.P., Amaral J.B., Hardt M., Marti L., Birbrair A., Jimenez M. (2024). Impact of social distancing from the COVID-19 pandemic on the immuno-inflammatory response of older adults. BMC Geriatr..

[20] Ma J., Hua T., Zeng K., Zhong B., Wang G., Liu X. (2020). Influence of social isolation caused by coronavirus disease 2019 (COVID-19) on the psychological characteristics of hospitalized schizophrenia patients: A case-control study. Transl. Psychiatry.

[21] Koyama Y., Nawa N., Yamaoka Y., Nishimura H., Sonoda S., Kuramochi J., Miyazaki Y., Fujiwara T. (2021). Interplay between social isolation and loneliness and chronic systemic inflammation during the COVID-19 pandemic in Japan: Results from U-CORONA study. Brain Behav. Immun..

[22] Blanco J.R., Albendin-Iglesias H., Negredo-Puigmal E., Barrios-Blandino A.M., Tomás-Jimenez C., Sanjoaquin-Conde I., Saumoy M., Pérez-Esquerdo V., Gonzalez-Cuello I., López-Lirola A.M. (2025). Lack of association between loneliness, social isolation and inflammation in people living with HIV aged ≥50 years: Results from the sub-study “No One Alone-Gesida Study”. PLoS ONE.

[23] Vingeliene S., Hiyoshi A., Lentjes M., Fall K., Montgomery S. (2019). Longitudinal analysis of loneliness and inflammation at older ages: English longitudinal study of ageing. Psychoneuroendocrinology.

[24] Hasselmo K., Mehl M.R., Tackman A.M., Carey A.L., Wertheimer A.M., Stowe R.P., Sbarra D.A. (2018). Objectively Measured Social Integration Is Associated With an Immune Risk Phenotype Following Marital Separation. Ann. Behav. Med..

[25] Jiang T., Yakin S., Crocker J., Way B.M. (2022). Perceived social support-giving moderates the association between social relationships and interleukin-6 levels in blood. Brain Behav. Immun..

[26] Shen C., Zhang R., Yu J., Sahakian B.J., Cheng W., Feng J. (2025). Plasma proteomic signatures of social isolation and loneliness associated with morbidity and mortality. Nat. Hum. Behav..

[27] Yoo J., Miyamoto Y., Ryff C.D. (2016). Positive affect, social connectedness, and healthy biomarkers in Japan and the U.S. Emotion.

[28] Jandackova V.K., Koenig J., Jarczok M.N., Fischer J.E., Thayer J.F. (2017). Potential biological pathways linking Type-D personality and poor health: A cross-sectional investigation. PLoS ONE.

[29] Ng T.K.S., Gan D.R.Y., Mahendran R., Kua E.H., Ho R.C. (2021). Social connectedness as a mediator for horticultural therapy’s biological effect on community-dwelling older adults: Secondary analyses of a randomized controlled trial. Soc. Sci. Med..

[30] Kim D.A., Benjamin E.J., Fowler J.H., Christakis N.A. (2016). Social connectedness is associated with fibrinogen level in a human social network. Proc. Biol. Sci..

[31] Walker E., Ploubidis G., Fancourt D. (2019). Social engagement and loneliness are differentially associated with neuro-immune markers in older age: Time-varying associations from the English Longitudinal Study of Ageing. Brain Behav. Immun..

[32] Ahmadian A.J., Lin J.E., Neylan T.C., Woolley J.D., O’Donovan A., Cohen B.E. (2020). Social integration and inflammation in individuals with and without posttraumatic stress disorder. Brain Behav. Immun..

[33] Ford J., Anderson C., Gillespie S., Giurgescu C., Nolan T., Nowak A., Williams K.P. (2019). Social Integration and Quality of Social Relationships as Protective Factors for Inflammation in a Nationally Representative Sample of Black Women. J. Urban Health.

[34] Ellis R.J., Iudicello J., Sun-Suslow N., Grelotti D., Cherner M., Morgan E., Letendre S.L., Heaton R.K. (2021). Social Isolation Is Linked to Inflammation in Aging People With HIV and Uninfected Individuals. J. Acquir. Immune Defic. Syndr..

[35] Braig S., Denkinger M.D., Dallmeier D., Klenk J., Rothenbacher D. (2025). Social isolation, loneliness and the relationship with serum biomarkers, functional parameters and mortality in older adults. Aging Clin. Exp. Res..

[36] Brown K.M., Diez-Roux A.V., Smith J.A., Needham B.L., Mukherjee B., Ware E.B., Liu Y., Cole S.W., Seeman T.E., Kardia S.L. (2020). Social regulation of inflammation related gene expression in the multi-ethnic study of atherosclerosis. Psychoneuroendocrinology.

[37] Elliot A.J., Heffner K.L., Mooney C.J., Moynihan J.A., Chapman B.P. (2018). Social Relationships and Inflammatory Markers in the MIDUS Cohort: The Role of Age and Gender Differences. J. Aging Health.

[38] Yang Y.C., Boen C., Gerken K., Li T., Schorpp K., Harris K.M. (2016). Social relationships and physiological determinants of longevity across the human life span. Proc. Natl. Acad. Sci. USA.

[39] Boen C.E., Barrow D.A., Bensen J.T., Farnan L., Gerstel A., Hendrix L.H., Yang Y.C. (2018). Social Relationships, Inflammation, and Cancer Survival. Cancer Epidemiol. Biomark. Prev..

[40] Lindsay E.K., Inagaki T.K., Walsh C.P., Messay B., Ewing L.J., Marsland A.L. (2022). Stress-Related Inflammation and Social Withdrawal in Mothers of a Child With Cancer: A 1-Year Follow-Up Study. Psychosom. Med..

[41] Gao Q., Bone J.K., Finn S., Fancourt D. (2024). The reciprocal associations between social deficits, social engagement, and inflammation: Longitudinal evidence comparing venous blood samples and dried blood spots and mapping the modifying role of phenotypic and genotypic depression. Brain Behav. Immun..

[42] Moieni M., Irwin M.R., Jevtic I., Breen E.C., Cho H.J., Arevalo J.M., Ma J., Cole S.W., Eisenberger N.I. (2015). Trait sensitivity to social disconnection enhances pro-inflammatory responses to a randomized controlled trial of endotoxin. Psychoneuroendocrinology.

[43] Henneghan A., Wright M.L., Bourne G., Sales A.C. (2021). A Cross-Sectional Exploration of Cytokine-Symptom Networks in Breast Cancer Survivors Using Network Analysis. Can. J. Nurs. Res..

[44] Pavela G., Kim Y.I., Salvy S.J. (2018). Additive effects of obesity and loneliness on C-reactive protein. PLoS ONE.

[45] MacAulay R.K., Timblin H.R., Tallman M.D. (2024). How Loneliness Gets Under the Skin: Inflammation Mediates the Relationship Between Loneliness and Gait Speed. Psychosom. Med..

[46] Canli T., Yu L., Yu X., Zhao H., Fleischman D., Wilson R.S., De Jager P.L., Bennett D.A. (2018). Loneliness 5 years ante-mortem is associated with disease-related differential gene expression in postmortem dorsolateral prefrontal cortex. Transl. Psychiatry.

[47] Hackett R.A., Poole L., Hunt E., Panagi L., Steptoe A. (2019). Loneliness and biological responses to acute stress in people with Type 2 diabetes. Psychophysiology.

[48] Tsai T.Y., Chiu C.J., Wang T.Y., Tseng H.H., Chen K.C., Chen P.S., Yang Y.K. (2022). Loneliness and isolated living status in middle-aged and older adults in Taiwan: Exploration on stress-related biomarkers, depressive symptoms, and disability. BMC Psychiatry.

[49] Nersesian P.V., Han H.R., Yenokyan G., Blumenthal R.S., Nolan M.T., Hladek M.D., Szanton S.L. (2018). Loneliness in middle age and biomarkers of systemic inflammation: Findings from Midlife in the United States. Soc. Sci. Med..

[50] Mezuk B., Choi M., DeSantis A.S., Rapp S.R., Diez Roux A.V., Seeman T. (2016). Loneliness, Depression, and Inflammation: Evidence from the Multi-Ethnic Study of Atherosclerosis. PLoS ONE.

[51] Wilson S.J., Koffer R.E. (2025). Lonely days: Linking day-to-day loneliness to biological and functional aging. Health Psychol..

[52] Wilson S.J., Marini C.M. (2024). Older Adults’ Social Profiles and Links to Functional and Biological Aging in the United States and Mexico. Psychosom. Med..

[53] Patapoff M.A., Jester D.J., Daly R.E., Mausbach B.T., Depp C.A., Glorioso D.K. (2024). Remotely-administered resilience and self-compassion intervention targeting loneliness and stress in older adults: A single-case experimental design. Aging Ment. Health.

[54] Ho R.T.H., Lo T.L.T., Fong T.C.T., Jiang D., Kwok J.Y.Y., Yeung D.Y.-L., Choi N.G., Warner L.M., Chou K.L. (2026). The mediating role of loneliness between psychosocial and physiological well-being in older adults. Psychoneuroendocrinology.

[55] Taylor K.S., Steptoe A., Iob E. (2022). The relationship of adverse childhood experiences, hair cortisol, C-reactive protein, and polygenic susceptibility with older adults’ psychological distress during the COVID-19 pandemic. Mol. Psychiatry.

[56] Xiang H., Gan X., Zhang Y., Zhang Y., Ye Z., Yang S., Huang Y., Wu Y., Zhang Y., Qin X. (2025). Association of Social Isolation and Plasma Metabolites With the Risk of Venous Thromboembolism. Arterioscler. Thromb. Vasc. Biol..

[57] Barrett-Young A., Ambler A., Cheyne K., Guiney H., Kokaua J., Tham Y.C., Williams M.J.A., Wilson G.A.M., Wong T.Y., Poulton R. (2023). Childhood Social Isolation as a Predictor of Retinal Neuronal Thickness in Middle Age: A Lifecourse Birth Cohort Study. Psychosom. Med..

[58] Li S., He L., Fang Y. (2025). From social isolation to sarcopenia: Heterogeneous social isolation patterns and inflammatory mediation. J. Gerontol. Ser. B.

[59] Xu L., Fu J., Zhai B., Li J. (2025). How social factors relate to arthritis risk in Chinese older adults: Population-based evidence. Front. Public Health.

[60] Kwon K.Y., Kim J. (2026). How social isolation accelerates biological aging: Exploring underlying behavioral and psychological mechanisms. Soc. Sci. Med..

[61] Kim B., Hu J. (2025). Longitudinal Associations of Social Isolation, Systemic Inflammation, and Cognitive Function Among Older Adults With Diabetes: Cross-Lagged Examination. Sci. Diabetes Self Manag. Care.

[62] Kahhale I., Puccetti N.A., Heller A.S., Hanson J.L. (2025). Probing connections between social connectedness, mortality risk, and brain age: A preregistered study. J. Pers. Soc. Psychol..

[63] Yu B., Steptoe A., Niu K., Ku P.W., Chen L.J. (2018). Prospective associations of social isolation and loneliness with poor sleep quality in older adults. Qual. Life Res..

[64] Ding L., Dai R., Qian J., Zhang H., Miao J., Wang J., Tan X., Li Y. (2024). Psycho-social dimensions of cardiovascular risk: Exploring the impact of social isolation and loneliness in middle-aged and older adults. BMC Public Health.

[65] Cho H.J., Seeman T.E., Kiefe C.I., Lauderdale D.S., Irwin M.R. (2015). Sleep disturbance and longitudinal risk of inflammation: Moderating influences of social integration and social isolation in the Coronary Artery Risk Development in Young Adults (CARDIA) study. Brain Behav. Immun..

[66] Roddick C.M., Christie C.D., Madden K.M., Chen F.S. (2021). Social integration after moving to a new city predicts lower systolic blood pressure. Psychophysiology.

[67] McMahon G., Griffin S.M., Borinca I., Bradshaw D., Ryan M., Muldoon O.T. (2024). Social integration: Implications for the association between childhood trauma and stress responsivity. Psychol. Trauma.

[68] Koh-Bell A., Chan J., Mann A.K., Kapp D.S. (2021). Social isolation, inflammation, and cancer mortality from the National Health and Nutrition Examination Survey—A study of 3,360 women. BMC Public Health.

[69] Molesworth T., Sheu L.K., Cohen S., Gianaros P.J., Verstynen T.D. (2015). Social network diversity and white matter microstructural integrity in humans. Soc. Cogn. Affect. Neurosci..

[70] Djekic D., Fagman E., Angerås O., Lappas G., Torén K., Bergström G., Rosengren A. (2020). Social Support and Subclinical Coronary Artery Disease in Middle-Aged Men and Women: Findings from the Pilot of Swedish CArdioPulmonary bioImage Study. Int. J. Environ. Res. Public Health.

[71] Ding L., Qian J., Dai R., Zhang H., Miao J., Wang J., Yu M., Tan X., Li Y. (2025). The hidden impact: Social isolation and inflammation’s role in pancreatic cancer risk among those with diabetes. BMC Cancer.

[72] Goldsmith D.R., Yuan Q.E., Addington J., Bearden C.E., Cadenhead K.S., Cannon T.D., Carrión R.E., Keshavan M., Mathalon D.H., Perkins D.O. (2025). The moderating role of lifetime social engagement on the relationship between C-reactive protein and negative symptoms among young adults at clinical high risk for psychosis. Brain Behav. Immun..

[73] Powell V.D., Kumar N., Galecki A.T., Kabeto M., Clauw D.J., Williams D.A., Hassett A., Silveira M.J. (2022). Bad company: Loneliness longitudinally predicts the symptom cluster of pain, fatigue, and depression in older adults. J. Am. Geriatr. Soc..

[74] Loeffler A., Steptoe A. (2021). Bidirectional longitudinal associations between loneliness and pain, and the role of inflammation. Pain.

[75] Theeke L., Carpenter R.D., Mallow J., Theeke E. (2019). Gender differences in loneliness, anger, depression, self-management ability and biomarkers of chronic illness in chronically ill mid-life adults in Appalachia. Appl. Nurs. Res..

[76] Vasan S., Lim M.H., Eikelis N., Lambert E. (2024). Investigating the relationship between early cardiovascular disease markers and loneliness in young adults. Sci. Rep..

[77] Akçay E., Çöp E., Dinç G.S., Göker Z., Parlakay A.Ö., Demirel B.D., Mutlu M., Kırmızı B. (2022). Loneliness, internalizing symptoms, and inflammatory markers in adolescent COVID-19 survivors. Child. Care Health Dev..

[78] Meneguzzo P., Terlizzi S., Maggi L., Todisco P. (2024). The loneliness factor in eating disorders: Implications for psychopathology and biological signatures. Compr. Psychiatry.

[79] Freilich C.D., Markon K.E., Mann F.D., Cole S.W., Krueger R.F. (2024). Associations Between Loneliness, Epigenetic Aging, and Multimorbidity Through Older Adulthood. J. Gerontol. Ser. B.

[80] Gong J., Preminger Z., Steptoe A., Fancourt D. (2025). Protein signatures associated with loneliness and social isolation: Plasma proteome analyses in the English Longitudinal Study of Ageing, with causal evidence from Mendelian randomization. Brain Behav. Immun..

[81] Simons R.L., Ong M.L., Lei M.K., Beach S.R.H., Zhang Y., Philibert R., Mielke M.M. (2023). Changes in Loneliness, BDNF, and Biological Aging Predict Trajectories in a Blood-Based Epigenetic Measure of Cortical Aging: A Study of Older Black Americans. Genes.

[82] Beach S.R.H., Klopack E.T., Carter S.E., Philibert R.A., Simons R.L., Gibbons F.X., Ong M.L., Gerrard M., Lei M.-K. (2022). Do Loneliness and Per Capita Income Combine to Increase the Pace of Biological Aging for Black Adults across Late Middle Age?. Int. J. Environ. Res. Public Health.

