# Supplementary material for: Relationship Between Social Isolation, Loneliness, and Systemic Inflammation: A Scoping Review of Biological Signatures and Clinical Outcomes
Source: Diseases. 2026 Oct 21;14(10):388. doi: 10.3390/diseases14100388 (PMC13703274; doi:10.3390/diseases14100388)
Supplement: Supplementary file 1 [file diseases-14-00388-s001.zip › diseases-4579269-supplementary.pdf]

Supplement

Summary of contents

Supplementary Data. Study characteristics, data extraction and notes

Supplementary Table S1. Database-specific search strings

Supplementary Table S2. Detailed study-level data extraction matrix for key included studies

Supplementary Table S3. Study characteristics by population subgroup

Supplementary Table S4. Biomarker panel and analytic methods across studies (including proteomics and genomics)

Supplementary Table S5. Preferred Reporting Items for Systematic reviews and Meta-Analyses extension for Scoping Reviews (PRISMA-ScR) Checklist

Supplementary Data. Study characteristics and data extraction

TITLE	AUTHORS/ DATE/ COUNTRY	TYPE OF RESEARCH	METHODOLOGY	POPULATION	MAIN FINDINGS	MEANINGS FOR CLINICAL PRACTICE	IMPACT ON POPULATION'S HEALTH	IMPLICATIONS FOR FEATURE RESEARCH
How social isolation accelerates biological aging: Exploring underlying behavioral and psychological mechanisms	Kwon KY, Kim J. Dec. 2025, USA.	Longitudinal analysis.	This study investigates how social isolation contributes to accelerated biological aging over time, focusing on potential behavioral and psychological mechanisms. Data were drawn from the Health and Retirement Study (HRS), involving 4036 U.S. older adults followed from 2006 to 2016. Accelerated biological aging at T3 (2016) was measured using an expanded phenotypic age derived from 22 clinical biomarkers. Social isolation was assessed at T1 (2006/2008), and behavioral (smoking, drinking, physical activity, poor sleep quality) and psychological	N = 4.036 adults aged 50 and older. The sample consisted of approximately 57 % females. In 2016 (T3), the mean expanded biological age was 73.37 years (SD = 10.90), which closely approximated the mean chronological age of 73.54 years (SD = 8.39).	Social isolation was longitudinally associated with accelerated biological aging after adjusting for sociodemographic, health, and psychosocial confounders. Among behavioral pathways, physical activity (20.3 %), smoking (8.5 %) and poor sleep quality (7.1 %) each accounted for a meaningful portion of the total effect. Depressive symptoms explained about 7.7 % of the association, whereas drinking did not demonstrate a significant mediating effect.	Those findings suggest that both health behaviors and psychological factors significantly explain the effects of social isolation on accelerated biological aging.	Those findings carry implications for public health and aging policy. Addressing social isolation requires both structural reforms and targeted interventions to prevent the cumulative disadvantages that contribute to physiological decline in later life. Age-friendly community initiatives may help promote meaningful social interaction among older adults.	Future research, applying inverse probability weighting that accounts for attrition over time or using more advanced methods to better estimate the effects of social isolation on biological aging in broader and more diverse samples.

			(depressive symptoms) factors were measured at T2 (2010/2012). Formal mediation analyses estimated indirect effects linking social isolation to accelerated aging.					
Lack of association between loneliness, social isolation and inflammation in people living with HIV aged ≥50 years: Results from the sub-study "No One Alone-Gesida Study"	Blanco JR et al. Dec 2025, USA.	Cross-sectional study.	This study was conducted by the AIDS Study Group of the Spanish Society of Infectious Diseases and Clinical Microbiology across 22 Spanish hospitals. Participants were classified based on whether they experienced loneliness (UCLA 3-item scale ≥6) and/or social isolation (Lubben Social Network Scale-6 ≤ 20). Blood samples were stored at −80°C, and cytokine levels were measured using the Olink Target 48 Cytokine panel. Multivariable median regression was used to assess differences in	A total of 199 PLWH (People living with HIV) were included in this analysis, aged 50 or older.55 of them were female and 144 were male. Mean age of the sample was 59.6 years old.	Among 199 PLWH, 33.2% reported loneliness/social isolation. Those without loneliness/social isolation were more likely to be male ($p = 0.023$), employed or students ($p < 0.001$) and had fewer prior AIDS events ($p = 0.045$) and comorbidities. CSF1 levels were elevated in unadjusted analysis (median difference 11.33; 95% CI 3.50–19.15, $p = 0.005$) but not in adjusted models.	These findings suggest that loneliness and social isolation may not directly influence inflammatory markers in older adults living with HIV, indicating that clinicians should consider other biological or psychosocial factors when assessing inflammation risk in this population.	The results highlight that although loneliness is common among older people living with HIV, it may not significantly affect inflammatory processes at the population level, suggesting the need to address multiple determinants of health in this aging population.	Further studies are needed to confirm these findings and to explore other potential factors that may influence these outcomes. Further research should also prioritize longitudinal, multi–time-point designs with expanded biomarker panels and test whether connection-enhancing interventions modulate inflammation.

			inflammatory biomarkers between those experiencing loneliness and/or social isolation and those experiencing neither. Questionnaires and venous blood sampling were conducted as part of the same study. Participants provided serum samples for biomarker analyses and gave written informed consent before any procedures.					
The mediating role of loneliness between psychosocial and physiological well-being in older adults	Ho RTH, et al. 2026, Hong Kong, China.	Cross – sectional study.	The present study recruited 141 older adults experiencing loneliness during the COVID-19 pandemic in Hong Kong. Participants completed a questionnaire that assessed loneliness, social network, perceived social support, and mindfulness, and provided six saliva samples for cortisol and C-reactive protein (CRP) over two days at baseline (T0) and 6-month follow-up (T1). Path analysis	N= 141 older adults experiencing loneliness, mean age = 64.1 years (SD = ± 4.38), 76 % female.	The participants showed small to moderate increases in perceived social support and mindfulness and small to moderate decreases in loneliness and pre-lunch cortisol. Greater improvements in social relationships were significantly associated with changes toward steeper diurnal cortisol slopes, indicating a healthier cortisol pattern. Greater	These findings support longitudinal associations between psychosocial functioning, mindfulness, and physiological markers in older adults in Hong Kong.	These results suggest that interventions targeting loneliness, alongside fostering social connections and mindfulness, may maintain or improve physiological well-being in older adults.	Future studies should examine psychosocial determinants of physiological functioning in post-pandemic contexts and consider objective measures such as actigraphy to monitor adherence to sample collection.

			was conducted to examine the associations of changes in psychosocial and physiological variables via change in loneliness.		improvements in mindfulness and social relationships were indirectly associated with greater reductions in CRP via a decrease in loneliness.			
From social isolation to sarcopenia: heterogeneous social isolation patterns and inflammatory mediation	Li S, et al. Nov. 2025, China.	Cross-sectional study.	Data were derived from the 2011 and 2015 waves of the China Health and Retirement Longitudinal Study, comprising 7.059 community-dwelling participants aged over 45. Sarcopenia was defined according to the Asian Working Group for Sarcopenia 2019 criteria. Social isolation was measured using a four-item scale, incorporating marital status, household structure, frequency of contact with children, and social activity participation. Latent class analysis (LCA) identified distinct social isolation patterns. Logistic regression assessed the effect of social isolation	N = 7.059 participants were included Non-isolated (*n* = 6.371), Isolated (*n* = 688). Female population of the total = 3.793.	During the 4-year follow-up, 490 participants developed sarcopenia. Socially isolated individuals had a significantly higher risk of sarcopenia (odds ratio [OR]: 1.34, 95% confidence interval [95% CI]: 1.01, 1.76) compared to their non-isolated peers. LCA identified three distinct social isolation patterns. Compared to the non-isolated groups, only individuals living alone and unmarried increased the risk of sarcopenia. Mediation analysis revealed systemic inflammation significantly mediated the association	This study investigates the mediating role of systemic inflammation in the association between social isolation and sarcopenia. Those disconnected from their families have a higher risk of sarcopenia. Interventions strengthening family ties may help prevent or delay sarcopenia progression.	Strengthening family connections and co-residence with family members can help maintain health and function in later life. Community-based interventions can provide essential social support, important in East Asian societies experiencing rapid population aging and a growing number of single-person households.	Future research with longer follow-up is needed to confirm these findings. Future studies should also consider more stable and specific biomarkers, such as soluble urokinase plasminogen activator receptor, which are more reliable to reflect chronic inflammatory processes.

			on sarcopenia. Inverse probability weighting was used to control potential selection bias. Mediation analysis examined the role of systemic inflammation.		between social isolation and sarcopenia, accounting for 4.95% of the total effect.			
The moderating role of lifetime social engagement on the relationship between C-reactive protein and negative symptoms among young adults at clinical high risk for psychosis	Goldsmith DR, et al. Oct. 2025, USA.	Case – control study.	Inflammatory markers have been shown to be elevated in Clinical High Risk for Psychosis (CHR-P) individuals and may be associated with negative symptoms. Social engagement in early developmental periods may decrease stress and interact with downstream processes, such as inflammation. Herein, we hypothesized that lifetime social engagement may moderate the association between C-Reactive Protein (CRP), a marker of inflammation, and negative symptoms in CHR-P young adults and healthy controls (HC) such that this association would be significant only among those at CHR-P with lower, but not	48 participants with an average age of 22.92 (SD = 3.37), 16 (33.3 %) were female, 21 (43.8 %) were White non-Hispanic, and 10 (20.8 %) had family income below the poverty line.	Simple slopes for the association between negative symptoms and CRP moderated by social engagement were calculated and stratified by CHR-P groups. CHR-P subjects had significantly greater negative symptoms than HC subjects ($p < 0.001$), though there was no significant difference in CRP values or social engagement. In the generalized linear models of the whole sample, negative symptoms were significantly associated with CRP ($\beta = 1.34$, SE = 1.35, 95 %CI −1.31–4.00, $p = 0.035$) as well as CHR-P ($\beta = 8.16$, SE = 1.71, 95 %CI	These findings suggest that social engagement may play a potential role in mitigating the adverse effects of stress, which may have subsequent downstream effects on inflammation and subsequent negative symptoms.	The data herein suggests that these associations in young individuals at risk for psychosis may be buffered by social engagement, perhaps by limiting stress and its downstream impacts on the brain and behavior.	Future longitudinal studies may be designed to address these intriguing relationships. Furthermore, future work may consider using approaches such as ecological momentary assessments, which have been used to examine social function in patients with psychotic illness. Additionally,future studies should include BMI or other metabolic markers in analyses alongside inflammation. Lastly, future

			greater, social engagement. 48 individuals (30 CHR-P and 18 healthy controls; HC) from the North American Prodromal Longitudinal Study (NAPLS)-2 cohort, were included in this analysis. Negative symptoms were assessed using the Scale of Psychosis-risk Symptoms (SOPS), and social engagement was calculated using the Life Events Stress scale. A generalized linear model with robust estimation was used to test the association of CRP, diagnosis, and social engagement (and their interactions) with negative symptoms, adjusting for age, sex, ethnicity, childhood poverty, and depressive symptoms.		4.80–11.52, $p < 0.001$). There was a significant association between negative symptoms and the interaction of CRP-by-social engagement ($\beta = 0.37$, $SE = 0.56$, 95 %CI −0.74–1.47, $p = 0.008$), but not the interaction of CRP-by-CHR-P or CHR-P-by-social engagement (both $p > 0.25$). There was a significant association between negative symptoms and the three-way interaction of CRP-by-CHR-P-by-social engagement ($\beta = -5.27$, $SE = 1.70$, 95 %CI −8.60 to −1.94, $p = 0.002$). Based on the simple slopes analysis, we observed a significant positive association between negative symptoms and CRP amongst the CHR-P group at low (−1SD; $p = 0.02$) and mean levels of social engagement ($p = 0.04$) but not in the			directions for this work may include testing novel treatment approaches, both pharmacologic and psychotherapeutic, that have the potential to increase social engagement, especially in CHR-P individuals with high inflammation.

					individuals with high social engagement (+1SD; p = 0.34) or in any of the HC social engagement levels (p all > 0.2). In this sample of CHR-P individuals, there was an association between negative symptoms and the interaction between diagnosis, CRP, and social engagement, adjusting for relevant clinical and demographic covariates. Greater engagement in social activities appeared to buffer the relationship between inflammation, as measured by CRP, and negative symptoms.			
Longitudinal Associations of Social Isolation, Systemic Inflammation, and Cognitive Function Among Older Adults with	Kim B, Hu J. Sep. 2025, USA.	Cross-Lagged Examination	Secondary data from the Health and Retirement Study were utilized across 3 waves (2006, 2010, and 2014). To examine the longitudinal relationships between social isolation and	1336 surveyed participants. Mean age = 68.66 years at baseline (SD = 9.28). female were 55%. White/Caucasia	Data from 1336 older adults with diabetes in the United States were analyzed. In the partially adjusted model, reciprocal negative associations between social isolation	The significant contribution of this study resides in elucidating the reciprocal longitudinal associations between social	The finding suggest that social isolation may contribute to systemic of inflammation and cognitive decline in population.	Future studies should consider other inflammatory biomarkers or stress-related physiological and psychological mediating factors

Diabetes: Cross-Lagged Examination			cognitive function, a cross-lagged panel modeling approach was employed, with particular attention to the mediating role of C-reactive protein. A sequential predictor procedure was used; initially, only social isolation and cognitive function were analyzed; subsequently, sociodemographic covariates were controlled for; and finally, health-related covariates were added.	n =76.6% (majority).	and cognitive function were identified. However, these reciprocal negative associations were no longer statistically significant after accounting for health-related covariates. C-reactive protein did not serve as a mediator in the link between social isolation and cognitive function regardless of adjustments for covariates.	isolation and poorer cognitive function among older adults with diabetes, even after controlling for sociodemographic covariates.	Addressing social isolation could therefore represent an important public health strategy to reduce inflammation and help prevent or delay cognitive impairment among older adults with diabetes.	as alternative mechanisms. Thus, is necessary to elucidate the underlying mechanisms between social isolation and cognitive function to interrupt potential vicious cycle leading to cognitive impairment and dementia.
Lonely days: Linking day-to-day loneliness to biological and functional aging	Wilson SJ, Koffer RE. May 2025, USA.	Longitudinal study.	Adults who participated in the National Study of Daily Experiences(NSDE) and Biomarker Project of the Midlife in the United States study (MIDUS) reported their daily loneliness on eight consecutive evenings, provided blood samples assayed for interleukin (IL)-6 and insulin-like growth factor-1 (IGF-1), and completed	N = 1,008 middle-aged and older adults. Study Wave 2 (2005–2009). Participants in this subsample ranged from 34 to 84 years (M = 55.34, SD = 11.66) and 54.6% were women.	Contrary to its traditional treatment as a trait, loneliness varied substantially day to day (intraclass correlation coefficient = 0.57). Controlling for age, gender, comorbidities, body mass index, education, and time between projects, higher daily loneliness was associated with lower IGF-1, weaker grip, slower gait, and	These findings reveal the unique importance of daily loneliness for objective measures of aging, extending beyond the widely studied effects of trait loneliness. Both higher daily loneliness and greater susceptibility to loneliness may	These associations foreshadow the potential promise of intervening on loneliness in daily life, where health risks unfold.	Future research should examine the degree to which daily signatures of loneliness remain stable over time and whether they predict longer-term prospective changes in unhealthy aging.

			assessments of gait speed and grip strength. Self-reports captured difficulties with instrumental activities of daily living, demographics, health conditions, and trait measures of depression and social connection.		more self-reported functional limitations. Those who were more susceptible to daily loneliness also had higher IL-6 and slower gait. Trait measures of social connection did not predict these outcomes, and daily loneliness measures were largely robust to the effects of depression.	share key roles in paths to unhealthy aging.		
The hidden impact: social isolation and inflammation's role in pancreatic cancer risk among those with diabetes	Ding L, et al. Jan. 2025, UK.	Longitudinal study.	UK Biobank participants with baseline diabetes diagnosis were analyzed. Social isolation was assessed through a questionnaire capturing five factors: household size, social activities, friend/family visits, loneliness, and confiding in others. Incident pancreatic cancer was identified using ICD codes. Baseline characteristics, insulin use, and other relevant factors were analyzed. Hazard ratios and	This study included 21,945 individuals with diabetes at baseline. Male participants = 13.707, female participants = 8.240. The mean age of the sample was approximately 60 years old.	Individuals with high social isolation were more likely to be male, smokers, non-drinkers, and have shorter sleep duration. They also had an increased risk of pancreatic cancer (HR = 2.65, 95% CI = 1.12–6.24) compared to those with low social isolation. Mediation analyses highlighted inflammation as a crucial mediator, with the proportion mediated by inflammation being	This study characterizes diabetes control status comprehensively and identifies social isolation as a significant psycho-social factor. The mediation effect of inflammation provides a mechanistic link, enriching our understanding of the complex trajectory of	The results address low-grade inflammation through lifestyle modifications and anti-inflammatory treatments may also be beneficial. These integrated approaches could improve outcomes in this high-risk population.	Future research should employ more extensive psycho-social assessments and biomarker measurements to further elucidate the intricate connections identified in our study.

			mediation analyses were conducted to determine the relationship between social isolation, inflammation, and pancreatic cancer risk.		19.44% for insulin use, 10.34% for smoking, and 8.33% for social isolation.	pancreatic cancer in individuals with diabetes.		
How social factors relate to arthritis risk in Chinese older adults: population-based evidence	Xu L, Fu J, Zhai B, Li J. Jun, 2025, China.	Cross-sectional study.	Arthritis status was assessed through self-reports, and key social factors-social network size, social participation, and social support were measured. Binary logistic regression models were used to analyze associations, adjusting for demographic and health-related variables.	A total of 15,854 individuals aged 60 and above were included. The mean age was 85.48, SD = ± 11.68 years old and 56.40% were female.	A larger social network was associated with a lower risk of arthritis (OR = 0.963, p = 0.015). Higher social participation, however, was associated with a higher risk (OR = 1.027, p = 0.007). Emotional support demonstrated a protective effect (OR = 0.963, p = 0.006), while instrumental support showed no significant impact. A significant interaction between social participation and emotional support (p = 0.008) indicated that emotional support mitigates the elevated risk of arthritis associated with high social participation.	Social factors play a crucial role in arthritis risk among older adults. Broader social networks and emotional support appear beneficial. Increased social participation may contribute to higher risk. These findings highlight the need for tailored interventions to promote healthy aging.	These findings suggest that strengthening the social network and prioritizing emotional support for older adults may be important strategies for reducing arthritis risk and improving overall health.	Future research could combine objective indicators, such as actual social interaction records, to improve the accuracy of the data. Furthermore, future research should further explore these relationships and their potential for intervention.

Social isolation, loneliness and the relationship with serum biomarkers, functional parameters and mortality in older adults	Braig S, et al. May 2025, Germany.	Cohort Study.	SI (Lubben Social Network Scale) from family, friends, and overall as well as loneliness (single direct question) were assessed at baseline in a population-based cohort study. Serum biomarkers and functional parameters measured at baseline and at three-year follow-up included high-sensitivity C reactive protein (hs-CRP), growth differentiation factor-15 (GDF-15), N-terminal pro-brain natriuretic peptide (NT-proBNP), high-sensitivity troponin I (hs-cTnI), high-sensitivity troponin T (hs-cTnT), gait speed, and hand grip strength. We used linear and Cox regression analyses adjusted for age and sex and established confounders.	A sample of 1.459 community-dwelling adults aged 65 + were participating in this study. The mean age was 75,6 years old, 638 (43,7%) were female.	High SI from friends was associated with small but significant adverse associations with some biomarkers (hs-CRP, GDF-15, hs-cTnT) at follow-up. High SI from family associated with NT-proBNP, high SI and moderate to severe loneliness with lower gait speed. Loneliness was linked to hs-CRP at baseline, but SI was a stronger predictor of biomarker levels. High SI overall (Hazard ratio 1.39, 95% CI 1.15, 1.67) was associated with increased 10-year mortality.	Social isolation, to a lesser extend loneliness, is consistently associated with decreased functional parameters such as gain speed and handgrip strength and a high risk for 10-year mortality.	Those findings suggest that social isolation and loneliness are associated with poorer functional health, unfavorable biological markers, and increased mortality risk in older adults, highlighting their importance as public health concerns in aging populations.	Further research should confirm those findings using e.g. a multidimensional assessment of loneliness.
Exome sequencing	Wang YX. et al,	Cohort study.	This cohort was from the UK Biobank, among	336,115 participants of	The study revealed 13 risk genes associated	The identification of gene variants is	The genetic correlation	The study provides a foundation for

identifies protein-coding variants associated with loneliness and social isolation	Apr 2025, U.K.		which whole exome sequencing data are available for participants. The researchers conducted the first exome-wide association analysis on loneliness and social isolation, utilizing participants for loneliness and for social isolation. Sensitivity analyses were performed to validate the genetic findings. They estimated the genetic burden heritability of loneliness and social isolation and provided biological insights into them.	white-British ancestry for loneliness and 346,115 for social isolation. The median age was 58.00 years, and 46.2 % were women for both. In total, 13,754,772 high-quality autosomal genetic variants remained in the exome sequencing data after variant QC (quality control).	with loneliness and 6 with social isolation, from which were identified six novel risk genes (ANKRD12, RIPOR2, PTEN, ARL8B, NF1, and PIMREG) associated with loneliness and two (*EDARADD* and *GIGYF1*) with social isolation through analysis of rare coding variants. Brain-wide association analysis uncovered 47 associations between identified genes and brain structure phenotypes, many of which are critical for social processing and interaction. Phenome-wide association analysis established significant links between these genes and phenotypes across five categories, mostly blood biomarkers and cognitive measures.	associated with loneliness and social isolation indicates that specific psychosocial conditions have also a biological background. Those findings contribute to understand better the risk mechanisms for psychiatric, physical disorders and in the development of mare personalized approaches for prevention and innervation.	between loneliness and social isolation demonstrates the completion of those phenomena with biological and social dimension. The understanding of those genetic factors can strengthen public's health strategies which aim at the reduction of psychosocial vulnerability and related diseases.	future studies aimed at exploring the functional mechanisms of these genes and their potential implications for public health interventions targeting loneliness and social isolation. In the future, it will also be essential to further validate our findings using larger datasets.
Plasma proteomic	Shen C. et al.	Cohort Study.	Data from participants	42,062	Proteins linked to these	This is the first	The exploration of	This research

signatures of social isolation and loneliness associated with morbidity and mortality	Mar. 2025,UK.		was collected and across 2,920 plasma proteins in the UK Biobank, the researchers characterized the proteomic signatures of social isolation and loneliness through proteome-wide association study and protein co-expression network analysis. Participant data include genome-wide genotyping, magnetic resonance imaging, electronic health record linkage, blood and urine biomarkers, as well as various other phenotypic endpoints. All participants in the UK Biobank provided informed consent, and ethical approval was obtained from the National Information Governance Board for Health and Social Care and the North West Multi-Centre Research Ethics Committee.	participants, individuals aged 40–69 years old recruited from 22 centers across the United Kingdom between 2006 and 2010. Mean age of the sample was 56.4 years old. 21.986 were female participants and 20.076 were male.	constructs were implicated in inflammation, antiviral responses and complement systems. More than half of these proteins were prospectively linked to cardiovascular disease, type 2 diabetes, stroke and mortality during a 14-year follow-up. Moreover, Mendelian randomization (MR) analysis suggested causal relationships from loneliness to five proteins, with two proteins (ADM and ASGR1) further supported by colocalization. These MR-identified proteins (GFRA1, ADM, FABP4, TNFRSF10A and ASGR1) exhibited broad associations with other blood biomarkers, as well as volumes in brain regions involved in interoception and emotional and social processes. Finally, the MR-identified proteins	study delineating robust and comprehensive plasma proteomic signatures associated with social isolation and loneliness. Comprehending the biology underlying the impact of social relationships on health, particularly the peripheral changes preceding disease, may provide new opportunities for targeted prevention and for effective intervention.	the peripheral physiology through which social relationships influence morbidity and mortality is timely and has potential implications for public health.	suggest that future validation in an independent dataset is essential.

					partly mediated the relationship between loneliness and cardiovascular diseases, stroke and mortality.			
Association of Social Isolation and Plasma Metabolites With the Risk of Venous Thromboembolism	Xiang H, et al. Feb. 2025, China.	Cohort study.	participants free of VTE (venous thromboembolism) and with complete metabolomics data at baseline from the UK Biobank were included. Social isolation was constructed based on the frequency of friend/family visits, weekly group activities, and living alone. The study outcome was incident VTE, including deep vein thrombosis or pulmonary embolism. Least absolute shrinkage and selection operator regression was applied to create a social isolation–associated metabolic signature. We estimated hazard ratios of study outcomes in relation to social isolation/metabolic	236.026 participants free of VTE and with complete metabolomics data at baseline from the UK Biobank were included. The mean age was 56.5 (SD = 8.1) years, 52.8% were female, and 95.2% of participants were White. Overall, 21 116 (8.9%) of the participants were in the social isolation group.	During a median follow-up of 12.5 years, 6681 VTE cases were recorded. Social isolation was positively associated with VTE risk (versus without social isolation; hazard ratio, 1.17 [95% CI, 1.09–1.27]). A metabolic signature for social isolation was constructed using 28 metabolites. This metabolic signature showed significant association with VTE risk (per SD increment; hazard ratio, 1.16 [95% CI, 1.13–1.19]) and mediated 11.0% (95% CI, 7.0–19.8) of the increased VTE risk associated with social isolation. 11 metabolites in the metabolic signature	No previous studies have investigated the association between social isolation and VTE risk.<br>This study addresses these knowledge gaps in a timely manner by using a prospective design and comprehensive adjustment for a range of potential confounding factors. This study also provides some new insights.	Those findings suggest the importance of managing social isolation and its associated metabolic disorders in the primary prevention of VTE.	More studies are required to further confirm the relationship between these metabolites and VTE risk and examine the underlying mechanisms. Further studies in other ethnic groups and populations are also needed to confirm those findings.

			signature and calculated the proportion of social isolation–VTE association mediated by the metabolic signature.		also significantly mediated the association between social isolation and VTE risk, with mediation proportions ranging from 0.5% to 2.6%. Similar results were found for incident deep vein thrombosis and pulmonary embolism, respectively.			
Probing Connections Between Social Connectedness, Mortality Risk, and Brain Age: A Preregistered Study	Kahhale I, et al. Jan. 2025, USA.	Cross-sectionalstudy.	This preregistered investigation uses the Midlife in the United States (MIDUS) study to examine connections between the quality of social connections, the brain-age gap, and markers of mortality risk to understand the longevity-promoting associations of social connectedness from a novel biological vantage point.	N= 197 participants. All participants were right-handed and were screened to ensure MRI compatibility. Mean age was 51 (SD = ±12)years old. 59% of the sample were female.	While social connectedness was associated with markers of mortality risk (number of chronic conditions and ability to perform activities of daily living), our models did not find significant links between social connectedness and the brain-age gap, or the brain-age gap and mortality risk. Supplemental and sensitivity analyses suggest alternate approaches to investigating these associations and overcoming limitations.	Self-reported positive relationships with others were associated with several operationalizations of mortality risk, including chronic health conditions, ability to achieve ADLs, and mortality.	These findings suggest that promoting social connectedness in the general population may contribute to healthier aging and potentially reduce mortality risk at the population level.	Future studies should continue to employ longitudinal modeling and consider multi-informant measures of these complex constructs.<br>It should also continue to consider whether it impacts neural markers of aging and longevity.

Psycho-social dimensions of cardiovascular risk: exploring the impact of social isolation and loneliness in middle-aged and older adults	Ding L, et al. Aug. 2024, UK.	Cohort study.	We utilized data from the UK Biobank, a large-scale study covering adults recruited from various centers across the UK between 2006 and 2010. We employed Cox proportional hazards models to analyze the relationship between social isolation, loneliness, and incident CVD (cardiovascular disease). CVD diagnoses were confirmed through hospital records and death-register data. Additionally, we conducted mediation analyses to assess the impact of low-grade inflammation.	The study encompassed 427,942 participants, aged 38 to 73, free from CVD, 55.8% of whom are women.	High levels of social isolation and loneliness were linked to a higher risk of CVD (HRs 1.11, 95% CI 1.06-1.16; HRs 1.17, 95% CI 1.11-1.23). Depression also emerged as a predictor of CVD onset (HRs 1.25, 95% CI 1.19-1.31), with each psychosocial factor independently contributing to increased CVD risk. Mediation analyses pinpointed inflammation as a crucial mediator, especially for loneliness (indirect effect proportion: 4.7%).	This study recognizes social isolation and loneliness as substantive psycho-social factors influencing CVD risk among individuals aged 38 and above. The discussion elevates these findings from mere observations to a deeper understanding of the complexities involved.	By embracing the nuanced interplay of sociodemographic, emotional, and biological factors, our research sets the stage for targeted, effective interventions, ultimately contributing to improved cardiovascular outcomes in this high-risk demographic.	Future studies should examine the longitudinal relationships between loneliness, social isolation and CV risk factors and evaluate interactions aimed at improving social connections to reduce CVD risk.
The loneliness factor in eating disorders: Implications for psychopathology and biological signatures	Meneguzzo P, et al. Jul. 2024, Italy.	Cross-Sectional study.	This study examined the interaction between loneliness, eating psychopathology, and biological markers in people with EDs (eating disorders). A group of 97 female patients with various diagnoses of ED was assessed for	The sample was made up of 97 white cisgender women, aged between 15 and 45 years. Mean age = 23,78 (SD = ± 7,74), Mean BMI = 18,97 (SD= ± 7,72).	The results indicated that individuals with ED who reported moderate to severe loneliness also displayed greater general psychopathology (p = 0.001), weight concerns (p = 0.007), and physical neglect	The findings consider notable association between loneliness and increased psychopathology was observed (both general and specific to eating),	Addressing loneliness may contribute to improving overall well-being and potentially support recovery. This consideration encompasses both psychological	Further research should explore diverse samples and longitudinal analyses for a broader generalization. Addressing loneliness is essential to

			loneliness, general and eating psychopathology, traumatic history during childhood, and clinical biomarkers such as C-reactive protein (CRP), erythrocyte sedimentation rate (ESR) and urinary-free cortisol (UFC).		during childhood ($p = 0.006$). Furthermore, people with higher levels of loneliness also had higher inflammatory indexes (ESR $p = 0.001$, CRP $p = 0.027$) and were positively correlated with markers of stress reaction such as UFC ($p < 0.05$).	as well as higher levels of inflammation and childhood physical neglect.	and physical factors that interplay in the clinical presentation of individuals.	improve the well-being and recovery of people with an ED.
The reciprocal associations between social deficits, social engagement, and inflammation: Longitudinal evidence comparing venous blood samples and dried blood spots and mapping the modifying role of phenotypic and genotypic depression	Gao Q, et al. Jul. 2024, USA-UK.	Cross-sectional study.	They used longitudinal nationally representative data from the US (Health and Retirement Study, 3 waves, 2006-16) and England (English Longitudinal Study of Ageing, 4 waves, 2004-18). Loneliness, social isolation, and social engagement were self-reported. CRP was measured using dried blood spots (US) and venous blood samples (England). Cross-lagged panel models were fitted and tested interactions with phenotypic depression (above-threshold depressive	They included 15,066 participants (mean age = 66.1 years, SD = 9.8, 53.8 % women) in the US and 10,290 (66.9 years, SD = 10.5, 53.2 % women) in England.	Reciprocal associations between loneliness and CRP were found, using dried blood spots and venous blood samples. Higher CRP predicted higher subsequent loneliness and higher loneliness predicted elevated CRP. Both phenotypic and genotypic depression modified this reciprocal association. There were also reciprocal associations for social engagement in venous blood samples: higher CRP predicted lower social engagement and greater social	This work highlights the importance of analyses using multiple cohorts from different countries, confirming whether underlying social-inflammatory processes are consistent and identifying individual or societal moderators responsible for any differences.	Loneliness and social engagement could provide modifiable targets for mitigating chronic inflammation, reducing long-term risk of chronic health conditions.	Future research should consider how findings are moderated depending on life transitions that disrupt social networks. Additionally, further research is encouraged to confirm whether measurement or demographic and cultural differences between samples explain differences.

			symptom scores) and genotypic depression (polygenic score for major depressive disorder).		engagement predicted lower subsequent CRP. Associations between social isolation and CRP were inconsistent and unidirectional.			
Older Adults' Social Profiles and Links to Functional and Biological Aging in the United States and Mexico	Wilson SJ, et al. Jun. 2024, USA and Mexico.	Cross – sectional study.	Participants in the Health and Retirement Study (HRS) and Mexican Health and Aging Study (MHAS) reported their loneliness, isolation (i.e., living alone), and relationship quality with spouse, children, and friends. Outcomes included C-reactive protein, functional limitations, self-rated health, comorbidities, gait speed, and grip strength. Models controlled for demographics, health behaviors, and body mass index.	HRS, N = 8316<br><br>MHAS, N = 15,001<br><br>Participants had similar age and gender distributions. Most HRS participants were White and highly educated, whereas MHAS participants did not report race and predominantly did not have an upper secondary education.	In both countries, five classes emerged, a supported group and four with elevated social stress: a) strained, b) isolated, c) spousal ambivalence, and d) unhappily married. Compared with the others, strained participants in both samples had greater functional limitations, poorer self-rated health, and more comorbidities, as well as slower gait in HRS and weaker grip in MHAS. Generally, supported participants fared better than the other groups. In HRS, C-reactive protein levels differed between the strained group and others, but these associations were	This study represents one of the first efforts to comprehensively characterize older adults' broader social landscape and to examine how complex social stress profiles are associated with functional and biological aging	These findings underscore the value of capturing a full view of the social world, both social disconnection and relationship quality, to understand their ties to healthy aging.	Future research should develop new methodological tools to enable robust quantitative comparisons across nonnested latent class models with large sample sizes, perhaps building on or borrowing from the lineage of coordinated analysis.

					explained by health behaviors and body mass index.			
Social integration: Implications for the association between childhood trauma and stress responsivity	McMahon G, et al. Apr. 2024, USA.	Cross – sectional study.	Using secondary data from the Midlife in the United States (MIDUS) Biomarker Project, they examine whether social integration can help explain the association between childhood trauma and lower CVR (cardiovascular reactivity). Participants completed a standardized laboratory stress paradigm which involved completing executive functioning (Stroop) and mental arithmetic (MATH) tasks. Cardiovascular measurements were continuously assessed using electrocardiogram (ECG) and Finometer equipment. The Social Well-Being Scale (Keyes, 1998) and the Childhood Trauma Questionnaire (CTQ; Bernstein et al., 2003) measured social integration and trauma,	N= 1,148, 652 of them were females. Mean age of the sample was 44 years old (SD = ± 12)	Regression analyses demonstrated that childhood trauma was associated with lower systolic (SBP; $\beta = -.14$, $p < 0.001$) and diastolic (DBP, $\beta = -.11$, $p < 0.001$) blood pressure reactivity but not heart rate (HR) reactivity. Mediation analyses, using Hayes PROCESS Model 4, showed that higher levels of trauma were associated with less social integration and in turn linked to lower reactivity across all biological indices. Moreover, sensitivity analyses showed that this indirect effect via social integration was evident for emotional and physical abuse, emotional and physical neglect, but not sexual abuse.	The findings suggest that higher levels of social integration may buffer the physiological stress response associated with childhood trauma. This indicates that social relationships could play a protective role in reducing stress-related biological dysregulation among individuals with early-life adversity.	Because childhood trauma and social isolation are relatively common exposures in the general population, the results highlight the potential importance of strengthening social integration as a public health approach to mitigate long-term stress-related health risks.	Future studies should use longitudinal designs to clarify causal pathways between childhood trauma, social integration, and stress physiology, and should investigate whether interventions aimed at increasing social connectedness can reduce stress reactivity and improve long-term health outcomes.

			respectively.					
How Loneliness Gets Under the Skin: Inflammation Mediates the Relationship Between Loneliness and Gait Speed	MacAulay RK, Feb. – Mar. 2024, USA.	Cross – sectional study.	Data from the Midlife Development in the United States Biomarker Project, was used for this study. The PROCESS macro tested the hypothesis that IL-6 would mediate the relationship between loneliness and gait, after adjusting for demographic and health risk factors.	National adult sample N = 822, age range 26-78 years. Mean age = 52.53 SD =13.46. 58% of the sample participants were women.	Age, sex, body mass index, waist-hip ratio and loneliness but not multimorbidity, depression history, depression symptoms, and number of friends, contributed to the variance in IL-6. Serial mediation analyses supported the chained effect of loneliness on walking time through BMI and IL-6. Results also showed specific indirect effects of BMI and IL-6 on walking time, suggesting more than one pathway by which loneliness influences health.	These results suggest that loneliness may increase the risk of systemic inflammation, leading to slowed gait and adverse health outcomes.	Psychosocial interventions that address loneliness may provide an optimal treatment target for reducing inflammation and preventing declines in health.	Future studies should use longitudinal and intervention designs to clarify mechanisms and examine whether reducing loneliness or inflammation can improve physical functioning in older adults.
Remotely administered resilience and self-compassion intervention targeting loneliness and stress in older adults: a single-case experimental	Patapoff MA, Jan. – Feb. 2024, USA.	Single-case experimental design (SCED).	This study utilized a multiple-phase-change single-case experimental design with three consecutive 6-week phases: control, intervention, follow-up. Assessments and biomarker collection (blood pressure,	N= 43, mean age = 76.9 (SD=7.5). The sample was 77% percent female.	Participants reported a reduction in stress ($p < 0.001$; $\eta p2 = 0.15$), depression ($p = 0.02$; $\eta p2 = 0.08$), and loneliness ($p = 0.003$; $\eta p2 = 0.18$), and an increase in self-compassion ($p = 0.01$; $\eta p2 = 0.13$) from	The present study indicates feasibility and efficacy of a remote resilience and self-compassion intervention for older adults targeting	The findings suggest that remotely delivered resilience and self-compassion interventions may help reduce loneliness and stress among older adults. If	Further work is needed to improve the generalizability of the intervention and to understand the potential physiological effects or mechanisms by which resilience

design			inflammation, sleep actigraphy) were conducted at each phase. Participants completed a 6-week remotely-administeredresilience and self-compassion intervention using techniques from cognitive behavioral therapy and resilience training. Repeated measures ANOVAs were conducted over the 12-week period from control (week 0) to intervention completion (week 12) and over the 18-week period from control (week 0) to follow-up (week 18) in supplemental analyses.		control to intervention completion (weeks 0-12). Post-hoc tests revealed that stress reduced significantly during the intervention phase (weeks 6-12) and loneliness reduced significantly during the control phase (weeks 0-6). Some improvements in blood pressure, inflammation, and sleep quality were noted in a subsample of participants.	loneliness and chronic stress. It also provides exploratory biomarker results on sleep and inflammatory correlates of loneliness and stress.	effective at a larger scale, such remote interventions could represent an accessible and scalable approach to improving mental well-being in aging populations, particularly for socially isolated individuals.	and self-compassion interventions work in older participants.
Investigating the relationship between early cardiovascular disease markers and loneliness in young adults	Vasan S, et al. Jan. 2024, Australia.	Cross-sectional study.	In the present study, we examined the relationship between loneliness and several early cardiovascular disease markers in young adults, after controlling for depression and social anxiety. Sixty-six young adults	N = 66 young adults, 18-35 years old, Mean age=22.7 females75.8%.	Results revealed higher loneliness was significantly associated with shorter pulse transit time ($\beta = -0.70$, $p = 0.002$; shorter pulse transit time is a subclinical marker for arterial stiffness). Additionally, results	We demonstrated higher loneliness is associated with poorer CVH, in particular impaired vascular function (i.e., a well-established risk factor for several CVDs) in	We also highlight the different pathways through which loneliness and depression may affect CVH in young adults, namely arterial stiffness and endothelial	Future researchers should adopt a longitudinal study design to potentially replicate and extend the findings from the current study and establish causality.

			completed psychological questionnaires and took part in several physiological tests assessing cardiovascular health (e.g., vascular function).		show that while loneliness and depression were both related to vascular dysfunction in young adults, the underlining physiological mechanisms through which they affect vascular function may be different. Specifically, higher loneliness was associated with increased arterial stiffness, whereas depression was associated with increased endothelial dysfunction ($\beta = -0.43$, $p = 0.04$).	young adults, after controlling for the influence of known confounding mental health issues.	function respectively.	For instance, future research could focus on investigating the influence of other common covariates related to CVDs.
Impact of social distancing from the COVID-19 pandemic on the immuno-inflammatory response of older adults	Nery GB et al. Jan. 2024, Brazil.	Cohort study.	Participants were followed at three different time points: before (S1) and after 6 (S2) and 20 months (S3) of the beginning of the COVID-19 pandemic. Unstimulated saliva samples were obtained to assess the levels of antibodies (secretory IgA, IgG and IgM) by	A sample of 11 older adults (mean age 66.8 ± 6.1), including 10 older women and 1 older man was included in this study.	None volunteer presented periodontal disease or caries. All volunteers received at least two doses of the COVID-19 vaccines after S2 and before S3. A tendency to increase salivary levels of SIgA and IgM at S2 and of IgG at S3 were observed compared to	This study provided an evaluation of the salivary levels of pro- and anti-inflammatory cytokines, as well as specific antibodies (sIgA, IgM, and IgG) for SARS-CoV-2 antigens in a	This study can improve our comprehension of the COVID-19 pandemic's impact on this population.	Future research should examine the long-term effects of social distancing on immune and inflammatory responses in older adults and evaluate whether interventions that reduce social

			ELISA (Enzyme-Linked Immunosorbent Assay) and cytokines (IL-2, IL-5, IL-6, IL-8 and IL-10, TSLP, IFN-γ, TNF-α) by multiplex analysis. Significant differences were evaluated using the Kruskal–Wallis test with Dunn's post-test.		the values found at S1 and S2. Significantly decreased levels of IL-2 and IL-5 were found at S2 and S3 ($p < 0.001$) time points. Lower levels of IFN-γ were found at S2 as compared to the values observed at S1 ($p < 0.01$). A significant decrease in the IFN-γ/IL-10 ratio was found at S2 ($p < 0.01$). When assessing the Th1/Th2 ratios, a significant decrease was found in the IFN-γ/TSLP ratio at S2 ($p < 0.001$) and S3 ($p < 0.001$) when compared to the values at S1. In addition, a significant increase was observed in the TNF-α/IL-5 ratio at S2 ($p < 0.001$) and S3 ($p < 0.001$) in comparison to the values at S1. In a similar way, an increase in the TNF-α/IL-6 ratio (Fig. 5E) was observed at S3 ($p < 0.001$) when compared to the values	group of older adults, both before and during the COVID-19 pandemic.		isolation can migrate adverse immunological outcomes.

					at S1.			
Associations Between Longitudinal Loneliness, DNA Methylation Age Acceleration and Cognitive Functioning	Lynch M, et al. Dec. 2023, USA.	Longitudinal study.	In the present study, they tested whether six common DNAm (DNA methylation) age acceleration measures mediated the effects of baseline loneliness and five different longitudinal loneliness trajectories on general cognitive ability, immediate memory recall, delayed memory recall, and processing speed in 1,814 older adults in the Health and Retirement Study (HRS).	The analytic sample consisted of 1,814 individuals. More than half (59.10%) of the sample was female. The sample was 77.29% White, 15.55% Black, and 7.17% “Other” race. The average age of the sample was 69.76 (*SD* = 9.77).	The researchers found that baseline loneliness and individuals who belong to the highest loneliness trajectories had poorer general cognitive ability and memory scores. Only DNAm age acceleration measures that index physiological comorbidities, unhealthy lifestyle factors (e.g., smoking), and mortality risk-mediated effects of baseline loneliness on general cognitive ability and memory functioning but not processing speed. These same DNAm measures mediated effects of the moderate-but-declining loneliness trajectory on cognitive functioning. Additionally, immediate and delayed memory scores were mediated by GrimAge Accel in	Current findings suggest that DNAm biomarkers of aging, particularly GrimAge Accel, have promise for explaining the prospective association between loneliness and cognitive functioning outcomes.	Given the high prevalence of loneliness among older adults, these result highlight loneliness as potential population – level risk factor for cognitive decline and unhealthy aging.	Future studies should investigate the biological mechanisms linking loneliness with epigenetic aging - cognitive decline and examine whether interventions that reduce loneliness can slow biological aging and protect cognitive function.

					the lowest and two highest loneliness trajectory groups. Total and mediated effects of loneliness on cognitive functioning outcomes were mainly accounted for by demographic, social, psychological, and physiological covariates, most notably self-rated health, depressive symptomatology, objective social isolation, and body mass index.			
Childhood Social Isolation as a Predictor of Retinal Neuronal Thickness in Middle Age: A Lifecourse Birth Cohort Study	Barrett-Young A, et al. Apr. 2023, New Zealand.	Cohort study.	Participants were members of the Dunedin Multidisciplinary Health and Development Study, a longitudinal population-based birth cohort from Aotearoa New Zealand, born 1972–1973 and followed until age 45, with 94% of the living cohort still participating. Social isolation was recorded prospectively at ages 5, 7, 9, and 11, from teacher and parent report. Retinal nerve fiber	N = 1037, 51.6% were male participants, born between April 1972 and March 1973 in Ōtepoti/Dunedin, New Zealand.	Childhood social isolation was associated with thinner average RNFL (B = −0.739, p = 0.02), nasal RNFL (B = −1.118, p = 0.005) and inferior RNFL (B = −1.524, p = 0.007), although only nasal RNFL remained significant after adjustment. These associations were not fully explained by other psychosocial or physical health risk	These findings provide some support for the hypothesis that RNFL thickness in midlife is influenced by childhood social isolation independent of co-occurring risk factors from across the life course.	These findings suggest that childhood social isolation, a form of psychosocial adversity, may exert long-term effects on the retina into adulthood.	Future studies should investigate the biological mechanism linking childhood social isolation with long – term neural changes and examine whether early social interventions can reduce these long – term health effects.

			layer (RNFL) and ganglion cell-inner plexiform layer (GC-IPL) thicknesses were measured via optical coherence tomography (OCT) at age 45.		factors in childhood or adulthood, nor were they mediated by adult loneliness or social support.			
The relationship of adverse childhood experiences, hair cortisol, C-reactive protein, and polygenic susceptibility with older adults' psychological distress during the COVID-19 pandemic	Taylor KS, et al. Dec. 2022, UK.	Longitudinal study.	The researchers tested the associations of ACEs(adverse childhood experiences), hair cortisol, C-reactive protein (CRP), and polygenic scores (PGS) with depression, anxiety, and loneliness among older adults during the COVID-19 pandemic, accounting for the potential interplay of ACEs with biological and genetic risk markers. Data were drawn from the English Longitudinal Study of Ageing, a prospective cohort study of older adults living in England. Retrospective information on ACEs were collected in 2006/7, while CRP and hair cortisol were measured	*N* = 2 050, mean age = 75.15 (SD = ±6.50), with 34% male and 66% female. Most participants (51.7%) had experienced no ACEs, whilst 22.3% and 26.0% experienced 1 and 2+ ACEs, respectively. Participants had mean cortisol (log) levels of ~2.13 (±1.50; *range* = −0.73–8.79) and 24.7% had high CRP.	The results showed that ACEs were associated with higher levels of depression (OR = 2.55[95%CI:1.81,3.59]) anxiety (OR = 1.84[95%CI:1.13,3.01]), and loneliness (b = 0.28[95%CI:0.14,0.42]) during the pandemic. Hair cortisol was related to an increased risk of depression (OR = 1.15[95%CI:1.04,1.26]), and CRP was associated with greater loneliness scores (b = 0.16[95%CI:0.03,0.30]). The relationship between cortisol and psychological distress was larger among participants with ACEs	This study suggests that individuals with ACEs are at greater risk of psychological distress during the COVID-19 pandemic. ACEs appear to be a more reliable marker of vulnerability compared to cortisol and CRP. However, dysregulated biomarkers of HPA-axis activity and inflammation pre-pandemic were also related to greater pandemic psychological	Given the recognized role of the HPA-axis in the development of mental health conditions, the stress response system may characterize one of the main psychobiological mechanisms underlying the association of early adversity with poor adulthood mental health.	Future longitudinal research that considers covariates including age, type and timing of adversity, different inflammatory markers (e.g., IL-6), and cortisol measurement, are necessary to understand the nuances of the biological pathways linking ACEs to psychopathology.

			at wave 6 (2012/13). Psychological distress was assessed before the pandemic (2018–19) and at two COVID-19 assessments in 2020 (June-July and November-December). Associations were tested on 2050 participants using linear/logistic regression models adjusted for pre-pandemic outcome measures and mixed-effect models to assess changes before and during the pandemic.		(e.g., ORdepression = 1.07[95%CI:1.00,1.14]). Further, individuals with high CRP experienced greater increases in feelings of loneliness from before to during the pandemic, compared to those with lower CRP levels (interaction effect=0.23; 95%CI:0.1-0.37). Individuals with 2+ ACEs experienced greater increases in depressive symptoms compared to those with none (interaction effect=2.09; 95%CI:1.1-3.98). Higher levels of hair cortisol were also related to worse changes in depressive symptoms across timepoints (interaction effect=1.84;95%CI:1.41 -2.41).	distress.		
Loneliness, internalizing symptoms, and inflammatory markers in	Akçay E, et al. Nov. 2022, Turkey.	Cross – sectional.	Adolescents and their parents were included in the study. This study assessed internalizing symptoms via Revised	74 adolescents, 41 girls, 55.4%, mean age 14.88, age range from 11 to	The most common disorder was Major Depressive Disorder (MDD) (25.7%), and 33.8% of the	This study indicates that anxiety, loneliness, and parental anxiety	Screening parental psychopathology and loneliness in COVID-19	In future studies, determining the psychosocial factors affecting the long-term

adolescent COVID-19 survivors			Children's Anxiety and Depression Scale (RCADS) and feelings of loneliness using the UCLA-loneliness scale. Baseline inflammatory markers at COVID-19 diagnosis were collected. Logistic regression analysis was used to determine predictors for depression in adolescents.	17 years) and parents (55 mothers, 74.3%) were included in the study.	adolescents were in the clinical range in at least one internalizing domain. Baseline C-Reactive Protein (CRP) levels correlated weakly with MDD scores. Loneliness scores correlated with all internalizing symptoms, strong association with MDD scores. Loneliness, anxiety, and parental anxiety were associated with an increased likelihood of MDD. Baseline CRP positivity did not predict MDD in adolescent COVID-19 survivors.	play an important role in adolescents' experience of depressive symptoms after COVID-19 infection	survivors seems to be preventive for adolescent mental health problems.	mental health of adolescents with COVID-19 seems to be necessary.
Bad company: Loneliness longitudinally predicts the symptom cluster of pain, fatigue, and depression in older adults	Powell VD,et al., Apr. 2022, USA.	Cohort study.	Data from the Health and Retirement Study were used. We included self-respondents ≥50 - year-old who had at least two measurements of loneliness and the symptom cluster from 2006–2016. Time-varying loneliness was used to predict pain,	N = 5974, age ≥50 years, 60.1% of them were female.	Loneliness increased the odds of subsequently reporting pain (aOR 1.22, 95% CI 1.08, 1.37), fatigue (aOR 1.47, 95% CI 1.32, 1.65), depression (aOR 2.33, 95% CI 2.02, 2.68), as well as the symptom cluster (aOR 2.15, 95% CI	Loneliness was found to predict a cluster of symptoms including pain, fatigue, and depression, suggesting that social isolation may contribute to the development	Given the high prevalence of loneliness among older adults, these findings indicate that loneliness may represent an important population-level risk factor for symptom burden	Future research should explore the biological and psychosocial mechanism linking loneliness with pain, fatigue, depression and examine interventions that reduce loneliness

			fatigue, depression, and the symptom cluster in the subsequent wave(s) using generalized estimating equations (GEE) and adjusting for sociodemographic covariates, living arrangement, and the presence of the symptom(s) at baseline.		1.74, 2.67). The median time between the baseline and final follow-up measurement was 7.6 years (IQR 4.1, 8.2).	or worsening of multiple interrelated health symptoms in older adults.	and reduced quality of life in aging populations.	to improve symptom outcomes in older adults.
Loneliness and isolated living status in middle-aged and older adults in Taiwan: exploration on stress-related biomarkers, depressive symptoms, and disability	Tsai TY et al. Mar. 2022, Taiwan.	Longitudinal study.	Participants separated into four groups: "Lonely and Isolated," "Not Lonely, but Isolated," "Lonely, but Not Isolated," and "Neither Lonely, nor Isolated," were retrieved from the Social Environment and Biomarkers of Aging Study conducted in 2000. Follow-up health indicators in 2006 included three stress-related biomarkers, depressive symptoms, and two physical disability indicators. A hierarchical regression	N= 629, aged 66.0 (SD=7.3), male = 58.8%, married =77.9% , 5.7% lived alone, and 14.5% were experiencing loneliness.	Firstly, compared to the "Neither Lonely nor Isolated" group, only the "Lonely, but Not Isolated" participants at baseline retained positive associations with the stress-related biomarkers levels 6 years later and the serum high sensitivity C-reactive protein (hsCRP) level (B=0.40, 95% CI=0.17-0.62)). However, such associations were not observed in the "Lonely and Isolated" participants. Secondly,	The findings suggest that loneliness and living alone have different effects on biological stress markers and mental health.	Loneliness was associated with higher levels of stress-related biomarkers (e.g., cortisol, IL-6, CRP) and greater depressive symptoms, indicating that loneliness may be an important population-level risk factor for poorer mental and physiological health in aging	The researchers suggest that both loneliness and isolated living status be included in future studies to explore broader pathophysiological indicators for both physical and mental health.

			was performed for the analysis.		only "Lonely and Isolated" participants at baseline were positively associated with depressive symptoms 6 years later (B=1.70, 95% CI=0.11-3.30). Finally, the associations between combinations of loneliness and isolated living status and physical disability were eliminated after adjusting the covariables.		populations.	
Perceived social support-giving moderates the association between social relationships and interleukin-6 levels in blood	Jiang T et al. Feb. 2022, USA.	Longitudinal study	This study examines whether perceived social support-giving (i.e., the belief that one can be available to give social support to others, henceforward referred to as perceived support-giving) moderates associations between social relationships and inflammation using data from the longitudinal follow-up of the National Survey of Midlife Development in the U.S.	N = 1054, healthy middle-aged adults who reside in the U.S. Ages ranging from 34 to 84 years, M = 55.26, SD = 11.78, 54.7% were female, 45.3% were male.	The results showed that perceived support-giving moderated the associations between IL-6 and indicators of positive social relationships, including social integration, perceived support-availability, and positive relations with others. Indicators of positive social relationships were associated with lower IL-6 among individuals higher, but	The current study showed that perceived support-giving moderates the associations between social relationships and IL-6. These findings provide a more nuanced understanding of the link between social relationships and systemic	Preliminary evidence indicated that the moderating effects of perceived support-giving might be further qualified by gender, being significant only in women.	Future work could identify the central and autonomic nervous systempathways by which positive social relationships lead to lower IL-6 for those who give more support.

			(MIDUS II). The participants completed self-report questionnaires on social integration, perceived support-availability from others, positive relations with others, perceived support-giving, socio-demographic information, and health-related information and provided blood samples for measurement of interleukin-6 (IL-6) as a marker of systemic inflammation.		not lower, in perceived support-giving. The moderating effects of perceived support-giving held after adjusting for socio-demographic and health-related covariates. Therefore, positive social relationships are associated with lower IL-6 only for individuals who believe they can give more support in those relationships. In addition, preliminary evidence indicated that the moderating effects of perceived support-giving might be further qualified by gender, being significant only in women.	inflammation.		
Stress-Related Inflammation and Social Withdrawal in Mothers of a Child With Cancer: A 1-Year Follow-Up Study	Lindsay EK, et al. Feb 2022, USA.	Cohort study.	Cancer-related stress (Impact of Events Scale), social network (social roles and contacts from the Social Network Inventory), and systemic inflammation (circulating interleukin [IL]-6) were assessed in 120 mothers three times after their	Participants were 120 female primary caregivers of a child (aged 0–17 years) newly diagnosed with cancer (mean age = 36 [SD = 8] years,	Consistent with predictions, greater cancer-related stress after diagnosis (T1) was associated with higher IL-6 after diagnosis (T1; b = 0.014, standard error [SE] = 0.01, p = 0.008). In turn, higher IL-6 after	This study provides a first indication that chronic stress-related systemic inflammation may predict changes in social behavior that associate with stress	These findings provide new insight into a complex adaptation process after childhood cancer diagnosis, whereby mothers' stress-induced	Future studies should examine long term interaction between stress, inflammation and social behavior in caregivers and evaluate interventions that

			child's cancer diagnosis: after diagnosis (T1), 6-month follow-up (T2), and 12-month follow-up (T3).	recruited from the Division of Hematology and Oncology, Children's Hospital of Pittsburgh.	diagnosis (T1) was associated with a decrease in social roles over time (T1 ➔ T3; B = -0.030, SE = 0.01, p = 0.041). Finally, dropping social roles over time (T1 ➔ T3) was associated with decreases in cancer-related stress (B = 25.44, SE = 12.31, p = 0.039) and slower increases in IL-6 (B = 1.06, SE = 0.52, p = 0.040) over time.	recovery and slower increases in inflammation in the year after a major life stressor.	inflammation may influence social network changes that are adaptive for their psychological and physical health over time.	reduce stress related inflammation and improve social support.
Social integration after moving to a new city predicts lower systolic blood pressure	Roddick CM, et al. Dec. 2021,USA.	Cohort study.	To examine whether moving to a new city is associated with increased CVD(cardiovascular disease) risk and whether the extent to which movers rebuild their social network after relocating predicts improved CVD risk and psychosocial well-being, recent movers, and age- and sex-matched nonmovers were followed over 3 months.	The first sample consisted of individuals who had moved to the city of Vancouver, Canada in the previous 6 weeks ("movers"n = 26, 13 women, Mean age = 30.62 years, SD = 7.35). The second sample consisted of	Multiple regression indicated higher systolic blood pressure (SBP) for movers (M = 107.42, SD = 11.39), compared with nonmovers (M = 102.37, SD = 10.03) at intake, though this trend was not statistically significant. As predicted, increases in movers' social network size over 3 months predicted decreases in SBP,	This study provides preliminary evidence for increased blood pressure, a marker of cardiovascular disease risk, among recent movers and suggests that this increased risk is mitigated by the extent to which movers	The current findings underscore the importance of building social connections to health and well-being, especially for new arrivals to the community. Public programs that help movers integrate more successfully into their host communities or	Future research should examine the long-term CV effects of social integration after relocation and explore interventions that help individuals build social networks in new environments.

			Blood pressure, C-reactive protein/albumin ratio (CRP/ALB), social network size, and psychosocial well-being were measured at intake (within 6 weeks of residential relocation for movers) and 3 months later.	individuals who had lived in Vancouver continuously for at least the past 5 years (“nonmovers” n= 20, 13 women, Mean age=31.4 years, SD = 7.10).	even after controlling for age, sex, and waist-to-hip ratio, b = -2.04 mmHg, 95% CI [-3.35, -.73]. Associations between increases in movers' social ties and decreases in depressive symptoms and stress were in the predicted direction but did not meet the traditional cutoff for statistical significance. Residential relocation and movers' social network size were not associated with CRP/ALB in this healthy sample. This study provides preliminary evidence for increased SBP among recent movers; furthermore, it suggests that this elevation in CVD risk may decrease as individuals successfully rebuild their social network.	successfully expand their social network in the months following their relocation.	that facilitate movers' access to affordable healthcare could act as a preventative measure against the development of more serious illness and longer-term burdens on the healthcare system.	
Getting under the skin: Social	Cudjoe TKM. et al. Oct. 2021,	Cross-sectional	Utilizing Round 7, conducted in 2017, data	N= 4.648, mean age was 76	After adjusting for age, gender, race/ethnicity,	These findings are important	In public health level those	Future studies should examine

isolation and biological markers in the National Health and Aging Trends Study	USA.	study.	from the National Health and Aging Trends Study (NHATS), they examine the relationship between social isolation and two biological markers: IL-6 and high-sensitivity CRP. This study included a nationally representative sample of Medicare beneficiaries 65 years and older who provided samples using dried blood spot (DBS) techniques. The defined social isolation utilizing a multi-domained typology that considers living arrangement, core discussion network, religious attendance, and social participation. IL-6 and CRP were obtained via DBS that were collected in Round 7 of the NHATS. They performed linear regression to examine the association between social isolation and biological markers IL-6 and CRP.	years old. Female= 55.4 % and male = 44.6. 81.4% were non-Hispanic white. Most of them were people with high income, at least one or more chronic conditions and had a robust phenotype.	income, tobacco use, body mass index, and chronic conditions, they found that severe social isolation and social isolation were significantly associated with higher levels of IL-6 and CRP values among older adults. However, no statistically significant difference was found between social connection groups due to race/ethnicity or BMI.	because clinical and social interventions to address social isolation among older adults may influence these biological processes and their potentially negative effects. Furthermore, biomarkers may serve as an important outcome measure for social isolation interventions.	findings indicate social isolation as social determine for older people’s health. The integration of social support strategies and community participation could contribute at the reduce of inflammation and the improvement of older population health.	the longitudinal mechanisms linking social isolation with biological dysregulation and investigate whether interventions that enhance social connections can improve biological health outcomes in older adults.

A Cross-Sectional Exploration of Cytokine-Symptom Networks in Breast Cancer Survivors Using Network Analysis	Henneghan A, et al. Sep. 2021, USA.	Cross – Sectional study.	Data collected from breast cancer survivors who were on average three years post chemotherapy completion. Perceived stress, fatigue, loneliness, perceived cognitive impairment, daytime sleepiness, and sleep quality were measured with self-report instruments, and a panel of 13 cytokines was measured from serum using multiplex assays. Symptoms and cytokines were simultaneously evaluated with correlations, network analysis, and community analysis.	Sample N= 66 women. Mean age=48.44 years old (SD= ±8.73), white and non-Hispanic.	Network analysis identified IL-2, granulocyte–macrophage colony-stimulating factor, IL-13, and perceived cognitive impairment as the most central nodes. Two communities were observed (symptoms and cytokines), connected by links including stress, fatigue, depression, and IL-2. Significant negative associations were found between IL-2 and fatigue, loneliness, stress, and perceived cognitive impairment. A significant negative relationship between perceived cognitive impairment and granulocyte-macrophage colony-stimulating factor.	This study provides evidence that IL-2 plays a mechanistic role in the co-occurrence of stress, loneliness, perceived cognitive impairment, and fatigue in BCS.	These findings add to a growing body of literature that is shifting to study symptoms as they co-occur, or cluster, rather than individual symptoms.	Future studies should include larger cytokine panels and prospective designs to better understand the strength and evolution of associations among cognitive impairment, stress, loneliness, fatigue and cytokines (particularly IL-2) in BCS and how these associations change over time.
Social connectedness as a mediator for horticultural therapy's	Ng TKS, et al. Sep. 2021, Asia.	Secondary analyses of a randomized controlled trial	The present study is a secondary analysis of a randomized controlled trial investigating the bio-psycho-social effects of	59 participants aged 60–85 years were recruited from a neighbourhood	At baseline, social connectedness was significantly correlated with IL-6 levels (β = −0.12, 95% CI = −0.21	This study demonstrated the efficacy of a primary preventive trial	When administering HT, interventionalists should include social	Future studies could further examine linkages with social epigenetics,

biological effect on community-dwelling older adults: Secondary analyses of a randomized controlled trial			HT (Horticultural therapy). Social connectedness was operationalized as positive relationships with others (PRWO), a sub-scale of the Ryff's scale of psychological well-being. IL-6 was quantified using a commercial ELISA kit. Outcomes were assessed at baseline, 3-month and 6-month post-intervention. Mediation analyses with bootstrapping were run to investigate our primary hypothesis. All analyses were controlled for covariates. All participants were screened for MoCA minimum score of 22 and were able to function independently.	and randomly allocated into the HT intervention arm (*n* = 29, mean age (*SD*) = 67.21 (4.52) 6 were male and 23 were female participants) or waitlist control arm (*n* = 30, mean age (*SD*) = 67.00 (4.18), 7 of the participants were male and 23 were female).	to −0.03, p = 0.008). Furthermore, social connectedness at 3-month significantly mediated the effects of HT on IL-6 levels at 6-month (β = 0.32, 95% CI = 0.09 to 0.54, p = 0.005, β = −0.25, 95% CI = −0.45 to −0.05, p = 0.016). Although seven participants and six participants were lost to follow-up in the HT and control groups, respectively, all the participants recruited were included in the analyses, as per intention-to-treat protocol.	among healthy older adults to prevent age-associated declines by ameliorating modifiable factors. It advances our understanding of cultural differences in the relationship between social connectedness and inflammatory markers with an Asian sample.	connectedness as a modifiable factor, which may ameliorate increased inflammation in older adults. Primary interventions in later life may improve health by regulating physiological markers that are implicated in multiple chronic conditions.	intersectionality, and health inequities. Furthermore, future studies should replicate these findings in larger samples.
Social isolation, inflammation, and cancer mortality from the National Health and Nutrition Examination Survey - a study of	Koh-Bell A, et al. Jul. 2021, USA.	Cross-sectional study.	Data were abstracted from the U.S. National Health and Nutrition Examination Survey from 1988 to 1994. The Social Network Index was used to assess participants' degree of social isolation.	N = 3360 women, mean age: 54 years, range: 40–89, 81.4% were White (majority). The most isolated, very	The most isolated participants were more likely to have low income (56.8% vs 12.2%, $p < 0.001$), have fewer years of education (40.8% vs 12.3%; $p < 0.001$), have	Social isolation was associated with higher inflammatory markers but not directly with cancer mortality, suggesting a	Given the high prevalence of social isolation, these findings indicate a potential public health burden through	Longitudinal studies are needed to clarify causal links and explore additional biological and behavioral pathways.

3,360 women			C-reactive protein and fibrinogen levels were included as markers of inflammation. We used the National Death Index to identify causes and dates of mortality. Chi-square and multivariable Cox regressions were employed for statistical analyses.	isolated, somewhat isolated, and not isolated comprised 14.5, 30.2, 37.1, and 18.2% of the sample, respectively.	low physical activity (27.3% vs 14.7%; $p < 0.003$), be obese (32.5% vs 24.4%; $p = 0.02$), and be current smokers (34.2% vs 10.3%; $p < 0.001$) compared to the not isolated ones. Mean fibrinogen levels increased with degree of social isolation ($p = 0.003$), but C-reactive protein showed no association ($p = 0.52$). Kaplan-Meier estimates indicated higher cancer mortality rates among participants with elevated fibrinogen levels, though not with statistical significance ($p = 0.08$). Furthermore, there was no association between social isolation and cancer mortality ($p = 0.54$). On multivariate analysis, obesity (HR = 1.56; 95% CI: 1.11–2.18), higher education (HR = 1.36; 95% CI:	potential biological pathway through inflammation.	inflammation-related disease risk.	

					1.01–1.83), and smoking (HR = 4.42, 95% CI: 2.84–6.88) were independent predictors for cancer mortality, while high physical activity predicted for lower mortality from cancer (HR = 0.67, 95% CI: 0.51–0.87). However, social isolation was not a predictor.			
Endocrine and immunomodulatory effects of social isolation and loneliness across adulthood	Zilioli S. Et al. Jun. 2021, USA.	Cross sectional study.	Participants from Midlife in the United States (MIDUS) Refresher study subsample completed both the biomarker project and salivary cortisol assessment component of the daily diary project. They tested the associations between two indicators of social isolation (living status and frequency of social contacts) and loneliness and daily cortisol secretion and two markers of systemic inflammation (C-reactive protein [CRP] and interleukin-6 [IL-6]) in a	Data for this study were drawn from a subsample (*N* = 314) of the Midlife in the United States (MIDUS) Refresher study. They used adults aged 25–75 years living in the US. the sample mean age was 47.70 years, with 53.5% of the sample being female, and 84.7% of the	Findings showed that, above and beyond loneliness, living alone was associated with a flattened diurnal cortisol slope (i.e., reduced changes in cortisol levels during waking hours that are indicative of a dysregulated HPA (hypothalamic-pituitary-adrenal) axis) and higher CRP levels. On the other hand, higher loneliness was associated with higher IL-6 levels, above and beyond our measures of social isolation.	This study reveals loneliness as a biological risk factor connected with inflammation and factors linked to cardiovascular and metabolic disease. Therefor evaluating loneliness seems to have an important clinical meaning at the risk assessment.	The link between loneliness and biological markers of inflammation makes social isolation more of a crucial matter of public health. Intervention such as building stronger social connection is more likely to reduce the cases of chronic disease on the39uality39io.	Future work should continue assess loneliness using multiple-item scales<br><br>Furthermore, future studies with larger sample sizes are required to corroborate our findings.

			sample of adults. Participants who completed the phone interview and self-administered questionnaire were eligible to participate in the biomarker project and the daily diary project.<br><br>Data for the biomarker project were collected during participants' 24-h stay at three General Clinical Research Centers: University of Wisconsin, Madison; University of California, Los Angeles, and Georgetown University, Washington, DC. The daily diary project included salivary cortisol assessment over 4 days and daily phone interviews over 8 days.	sample being White.	Loneliness did not mediate any of the effects of social isolation on either cortisol or CRP, and age did not moderate any of the relationships reported above. Those findings support the idea that social isolation and loneliness have unique and independent endocrine and immune effects despite being linked to each other.			
Interplay between social isolation and loneliness and chronic systemic inflammation during the COVID-19 pandemic in	Koyama Y et al. May 2021, Japan.	Descriptive study.	The data from the Utsunomiya COVID-19 seROprevalence Neighborhood Association (U-CORONA) study, which targeted randomly	624 participants were analyzed, aged 18–92 years, mean age 51.4 years old. 293 of them were male and	Isolated-Lonely condition (i.e., being both socially isolated and feeling lonely) was associated with higher NLR among men (B = 0.141, 95%CI = −0.01	Those findings emphasized the importance of assessment of both subjective and objective social	Those results indicated that not only socially isolated people but also those who were socially connected with	Future studies to identify the populations at risk and the complex interplay between subjective and objective social

Japan: Results from U-CORONA study			sampled households in Utsunomiya city, Japan, were analyzed. Social isolation was assessed as a structural social network by asking the number of social roles they have daily. Loneliness was measured with the UCLA loneliness scale. As chronic inflammation biomarkers, neutrophil-to-lymphocyte ratio (NLR) and the concentration of high-sensitivity C-reactive protein (CRP) were measured. Generalized estimating equations method was employed to take into account the correlations within households.	331 were female.	to 0.29). Interestingly, Nonisolated-Lonely condition (i.e., not socially isolated but feeling lonely) was associated with lower CRP among women (B = −0.462, 95%CI = −0.82 to −0.10) and among the working-age population (B = −0.495, 95%CI = −0.76 to −0.23). In conclusion, being both socially isolated and feeling lonely was associated with chronic inflammation.	relationships not only for future research, but also for the intervention perspective especially under the COVID-19 pandemic.	loneliness may need help.	relationships are warranted.
Social Isolation Is Linked to Inflammation in Aging People With HIV and Uninfected Individuals	Ellis RJ, Apr. 2021, USA.	Cross-sectional study.	People with and without HIV infection were enrolled in a community-based, single center study. Primary predictors were the Medical Outcomes Study (MOS) Social Support Survey	Participants were 69 PWH (people with HIV) and 87 HIV−, 31.4% were female, mean age = 44.9, SD=14.5.	PWH had worse Positive Social Support (p = 0.0138) and Affectionate Support (p = 0.0078) than did HIV− individuals. A factor analysis was used to group the	These results suggest that enhancing social support might be an intervention to reduce inflammation and its associated	The finding suggest that social isolation may contribute to increase inflammation among aging individuals with	These results provide a springboard for future research on the mechanisms linking social support and

			and outcomes were a panel of inflammatory biomarkers (ICAM-1, MCP-1, IL-6, IL-8, IP-10, CRP, D-Dimer, VEGF, sCD14 and uPAR) in blood plasma and cerebrospinal fluid (CSF).	Non-Hispanic white =51.9%, Hispanic =27.6%, Black =14.1% and other ethnicities= 6.4%. Among PWH, 55 (80.9%) were virally suppressed.	biomarkers into related categories separately for each fluid. Levels of three of the four Plasma Factors were significantly higher in PWH than HIV− (ps = 0.007, 0.001 and 0.0005, respectively). Levels of one of the three CSF Factors also were significantly higher in PWH than HIV− p = 0.0194). In the combined PWH and HIV− cohort, poorer social support was associated with higher levels of a factor in plasma loading on MCP-1, IL-8 and VEGF (p = 0.020), and with a CSF factor loading on MCP-1 and IL-6 (p = 0.006).	adverse outcomes among PWH.	and without HIV, highlighting the importance of social support as a potential public health factor that influence health outcomes in older population.	inflammation.
Influence of social isolation caused by coronavirus disease 2019 (COVID-19) on the	Ma J et al. Nov. 2020, USA.	Case – control study.	This study compared the differences in common inflammatory indexes and psychological characteristics between	N=30 patients with schizophrenia were recruited from Wuhan	The Chinese Perceived Stress Scale (CPSS) score, Hamilton Depression Scale (HAMD) score and	The results of this study indicate that inpatients suffered from social isolation due to	The increased psychological burden on hospitalized schizophrenic	Future research is needed to examine the long – term psychological

psychological characteristics of hospitalized schizophrenia patients: a case-control study			isolated patients in the epicenter of the epidemic and unquarantined patients. Furthermore, it analyzed the differences in the above indicators among the isolation group over time (i.e., before and after isolation).<br><br>These patients were medically isolated from 10 January 2020 to 30 April 2020.	Mental Health Center. In addition, N= 30 ordinary schizophrenic patients were matched with the isolation group and were recruited from another branch of Wuhan Mental Health Center as controls.<br><br>At the isolation group 18 of the participants were female and 12 were male. The mean age of this group was 43.17 years old. In the control group 15 female and 15 male participants were recruited with mean age to be 45 years old.	Hamilton Anxiety Scale (HAMA) score of the isolation group were significantly higher than those of the control group (p = 0.00, 0.00, 0.00, respectively). The C-reactive protein (CRP) level, CPSS score, HAMA score and Pittsburgh sleep quality index (PSQI) score of the isolation group were significantly higher after isolation (p = 0.01, 0.00, 0.00, 0.00, 0.00, respectively). Inpatients of schizophrenia suffered from social isolation due to COVID-19 have a severe psychological burden. Social isolation caused patients to develop a weak inflammatory state and led to worse anxiety and sleep quality	COVID-19 have higher levels of psychological stress and more severe anxiety and depression than those in hospitals that are not suffered from. After social isolation, inpatients with schizophrenia showed higher levels of CRP and psychological stress, more severe anxiety, and worse sleep quality.	patients caused by COVID-19-induced social isolation led to a weak state, increased anxiety and decreased sleep quality.	effects of social isolation in patients with schizophrenia and evaluate interventions aimed at improving social support and mental health outcomes during and after pubic health crises.
Social integration	Ahmadian AJ.	Cross-	This study examined	Our sample	Social isolation, but not	In the present	These contrasting	Further research

and inflammation in individuals with and without posttraumatic stress disorder	Et al. Oct. 2020, USA.	sectional study.	relationships among social isolation, perceived social support, and inflammation in Veterans Affairs (VA) patients with and without PTSD. Social isolation was assessed with the Berkman Syme Social Network Index, perceived social support with the Multidimensional Scale of Perceived Social Support, and PTSD with the Clinician Administered PTSD Scale. Inflammation was indexed by high sensitivity C-reactive protein, white blood cell count, and fibrinogen. Hierarchical linear regression was used to examine associations between social measures and inflammation. PROCESS was used to examine the interactive effects of social relationships and PTSD on inflammation.	included 735 (35% PTSD+) participants from the Mind Your Heart Study (mean age = 58 ± 11, 94% male).	low perceived social support, trended towards an association with elevated inflammation in the full sample. However, considering groups with and without PTSD separately, social isolation was significantly associated with all inflammatory markers among individuals without PTSD, but not among those with PTSD.	study, they found that individuals with PTSD had elevated inflammation regardless of level of social integration, whereas social integration was associated with reduced levels of inflammatory markers among individuals without PTSD.	findings for objective and perceived social support may have implications for the design of public health and clinical socially supportive interventions.	could help determine if interventions to improve the nature and quality of social support would be effective in reducing inflammation in those with or without PTSD.
Bidirectional	Loeffler A, et al.	Longitudinal	The role of inflammation	A sample of	We found that baseline	Some distinctions	Both pain and	Future studies

longitudinal associations between loneliness and pain, and the role of inflammation	Sep. 2020, UK.	study.	in these links was also investigated. Pain was defined by reports of being often troubled by pain at a moderate or severe intensity, whereas loneliness was measured using the shortened UCLA scale. Age, sex, ethnicity, educational attainment, wealth as a marker of socioeconomic resources, marital status, physical activity, and depressive symptoms were included as covariates.	4906 (2158 men and 2748 women, mean age = 65.1 SD= ± 8.72 years) who were participants in the English Longitudinal Study of Ageing were recruited.	loneliness was associated with pain 4 years later after adjusting for baseline pain and other covariates (odds ratio [OR] = 1.25, 95% confidence interval [CI] 1.06-1.47, *P* = 0.007). Similarly, baseline pain independently predicted loneliness 4 years later (OR = 1.34, 95% CI 1.14-1.58, *P* = 0.001). Associations remained significant after additional adjustment for baseline mobility impairment. Likelihood of pain on follow-up was heightened when baseline loneliness was accompanied by elevated C-reactive protein concentration (OR = 1.50, 95% CI 1.13-2.00, *P* = 0.006), whereas inflammation did not predict future loneliness or contribute to the association between baseline pain and future loneliness.	were found between the mechanisms involved in these relationships, with systemic inflammation playing a more prominent role in the association between loneliness and subsequent pain than in the link between pain and later loneliness.	loneliness are distressing experiences that impact well-being and quality of life. Greater understanding of these may help identify methods for breaking these links.	should further investigate the biological mechanisms linking loneliness inflammation and pain using longitudinal designs with repeated inflammatory measurements and explore whether intervention targeting loneliness can reduce inflammation and pain symptoms.

					Both pain and loneliness are distressing experiences that impact well-being and quality of life.			
Social Regulation of Inflammation Related Gene Expression in the Multi-Ethnic Study of Atherosclerosis	Brown KM, et al. Jul.2020, USA.	Cross-sectional study.	Social factor and gene expression data were collected from the Multi-Ethnic Study of Atherosclerosis (MESA). Inflammation-related genes were identified from the Gene Ontology database. The associations between social factors and gene expression were first assessed using the Global Analysis of Covariance (Global ANCOVA) gene set enrichment test. When the global test was significant, linear regression and elastic net penalized regression tests were employed to identify the individual gene transcripts within each gene set associated with the	1,264 individuals were recruited. The mean age was 69.6 years and 51% of the sample was female	Loneliness (p=0.003), chronic burden (p=0.002), and major or lifetime discrimination (p=0.045) were significantly associated with global expression of the chronic inflammatory gene set. Of the 20 transcripts that comprise this gene set, elastic net selected 12 transcripts for loneliness, 8 for chronic burden, and 3 for major or lifetime discrimination. Major or lifetime discrimination was also associated with the inflammatory response (p=0.029), regulation of the inflammatory response (p=0.041), and immune response (p=0.025) gene sets in global analyses, and 53, 136,	This study highlights gene expression as a biological mechanism through which social factors may affect inflammation.	Better understanding of the quality through which social factors act biologically will enable the development of more precise prevention and treatment approaches.	To identify the most salient social factors, future research should replicate these tests in diverse study samples and assess how the characteristics of social factors affect association with differential gene expression patterns.

			social factor.		and 26 transcripts were selected via elastic net for these gene sets respectively. There were no significant associations in linear regression analyses after adjustment for multiple testing.			
Social Support and Subclinical Coronary Artery Disease in Middle-Aged Men and Women: Findings from the Pilot of Swedish Cardiopulmonary bioimage Study	Djekic D, et al. Jan. 2020, Sweden.	Cross-sectional study.	This study investigated the relationship between social support and subclinical CAD among 1067 healthy middle-aged men and women. Social support was assessed with validated social integration and emotional attachment measures. Subclinical CAD was assessed as a coronary artery calcium score (CACS) using computed tomography.	N= 1067 men and women aged 50–64 years. The mean age of the sample was approximately 58 years old.	There was no association between social support and CACS in men. In women, low social support was strongly linked to cardiovascular risk factors, high levels of inflammatory markers, and CACS > 0. In a logistic regression model, after adjustment for 12 cardiovascular risk factors, the odds ratio (95% confidence intervals) for CACS > 0 in women with the lowest social integration, emotional attachment, and social support groups (reference: highest	This study showed an association between lack of social support and subclinical CAD in middle-aged women, but not in men. Lack of social support may affect the atherosclerotic process and identify individuals vulnerable to CAD events.	Lack of social support may help to identify individuals vulnerable to CAD events, and these subjects should be screened for traditional CVD risk factors.	Future longitudinal studies are needed to clarify casual relationships and determine whether improving social support can reduce cardiovascular risk.

					corresponding group) were 2.47 (1.23-5.12), 1.87 (0.93-3.59), and 4.28 (1.52-12.28), respectively. Using a machine learning approach (random forest), social integration was the fourth (out of 12) most important risk factor for CACS > 0 in women. Women with lower compared to higher or moderate social integration levels were about 14 years older in “vascular age”.			
Social engagement and loneliness are differentially associated with neuro-immune markers in older age: Time-varying associations from the English Longitudinal Study of Ageing	Walker E et al. Nov. 2019, England.	Cross-sectional study.	The researchers analyzed blood samples from adults from the English Longitudinal Study of Ageing across three waves of data collection: 2004/5, 2008/9 and 2012/2013. They used fixed effects modelling to estimate the relationship between loneliness, social isolation, living alone and levels of fibrinogen,	Of the 8780 participants, 55% were female, age range was 52–99.	Higher levels of social engagement and living with somebody were associated with lower levels of CRP, fibrinogen and WBC, while lower levels of loneliness were associated with higher levels of IGF-1. These associations were found to be independent of time-invariant factors such	This study was the first to simultaneously examine social engagement, living with somebody, loneliness and biomarkers in longitudinal data and showed differential associations with CRP, fibrinogen,	Aspects of social engagement were associated with lower levels of inflammation whilst loneliness was inversely related to inflammation. This suggests there could be different biological pathways involved in objective and	Future studies could explore whether social interventions for older adults could help in the prevention of biological changes associated with the onset of mental and physical poor health. Additionally,

			insulin like growth factor-1 (IGF-1), white blood cell (WBC) count and C-reactive protein (CRP), whilst accounting for all time-invariant and identified time-varying confounders.		as gender, medical history, previous patterns of social behaviors, unobserved aspects of social class, and genetics, and time-varying factors such as income, physical health, health behaviors, and depression.	WBC count and IGF-1.	subjective aspects of social connections.	whether the pattern of findings is corroborated when considering a broader panel of immune biomarkers.
Longitudinal analysis of loneliness and inflammation at older ages: English longitudinal study of ageing	Vingeliene S et al. Aug. 2019, England.	Longitudinal analysis	This study uses data from English Longitudinal Study of Ageing (ELSA), which began in 2002 with over 11,000 respondents aged 50 years or older, living in private households in England (Steptoe et al., 2013). The initial sample was selected from the participants in the Health Survey for England in 1998, 1999, and 2001, using a multistage random sampling strategy. Two-yearly follow-up consisted of personal interviews and self-completion questionnaires. Every	Participants in the English Longitudinal Study of Ageing (n = 3239) aged 50 years or older with an average age of 64 years, provided data in waves 4 (2008/2009) and 6 (2012/2013). Men were 1.462 and women were 1777.	Linear regression conditional change models had three outcomes: C reactive protein (CRP) measured in mg/L (log transformed), fibrinogen in g/L and ferritin in g/dL. In men, the onset of loneliness indicated by answering ‘no’ at wave 4 and ‘yes’ at wave 6 to question “Much of the time during the past week, you felt lonely?” was associated with a statistically significant increase in levels of CRP (β = 0.36, 95% confidence interval	These findings may help to shed light on the mechanisms underlying thequality of loneliness with disease and mortality risk.	Combined social and targeted medical interventions may help to reduce health risks associated with loneliness.	Future studies should investigate the casual mechanism linking loneliness with inflammation processes and explore whether interventions aimed at reducing loneliness can improve inflammation profile and health outcomes in older adults.

			four years, in addition to the main interview, all study participants were asked if they would like to take part in data collection conducted by a registered nurse during a home visit. The nurse visit included a personal face-to-face computer assisted interview, collection of biological samples and anthropometric measurements such as height and weight. A blood sample was collected only once during the scheduled visit. Participants provided signed informed consent for the main interview, nurse visits, and blood sampling. Ethical approval was obtained from the London Multi-Centre Research Ethics Committee (MREC/01/2/91). We used data from waves 4 (2008/09) and 6 (2012/13).		(0.09 to 0.62)), plasma fibrinogen (0.18 (0.04 to 0.31)) and ferritin (41.04 (6.58 to 75.50)), after full adjustment. A statistically significant increase in CRP in men was also observed for onset of loneliness assessed with the question “How often do you feel lonely?” (0.20 (0.03 to 0.38)). These associations were not mediated by depressive symptoms. Persistent loneliness (loneliness experienced at both baseline and follow-up) assessed using the University of California Los Angeles (UCLA) loneliness scale was associated with an increase in CRP (0.11 (0.004 to 0.22)) among men. Associations of the two latter loneliness measures with fibrinogen and ferritin were mainly null. Among women, the only statistically significant association			

					was for persistent loneliness (loneliness at both waves) identified by question "Much of the time during the past week, you felt lonely?" with a reduction in levels of ferritin (-20.62 (-39.78 to -1.46)).			
Social Integration and Quality of Social Relationships as Protective Factors for Inflammation in a Nationally Representative Sample of Black Women	Ford J, et al. Mar. 2019, USA.	Cross-sectional study.	They investigate the association between social relationships and inflammation—a known CVD (cardiovascular disease) risk factor—in Black women, a population with higher rates of CVD and CVD mortality. Social integration was a ***z***-score standardized measure of four items (marital/cohabitation status, church attendance, volunteerism, close friendships). Data on the quality of three relationship types was available: perceived happiness with a	Secondary data from wave 4 of the National Longitudinal Study of Adolescent to Adult Health (Add Health) were analyzed for this study. The sample was comprised of 1829 Black women aged 24–34 years.	No significant associations were found between level of social integration and hs-CRP levels. With respect to relationship quality, women who reported they were very happy with their romantic relationship were less likely than those who were only fairly happy or unhappy to have hs-CRP levels in the moderate- (AOR = 0.36, 95% CI = 0.17, 0.75), high (AOR = 0.20, 95% CI = 0.08, 0.49), or very high CVD–risk category (AOR = 0.36, 95% CI = 0.16, 0.80).	This study highlights the importance of social relationships to young adult Black women's immune health.	As the evidence is strong on the benefit of building social support to enhance the effectiveness of behavior change interventions, our findings reinforce the need to consider these approaches for promoting Black women's cardiovascular health.	Further research with the longitudinal social relationship and inflammatory measures are needed to better understand how changes in social relationships may influence CVD risk over the life course. Furthermore, future research may want to consider using continuous response options, as cultural differences in the definition of social

			romantic relationship and perceived closeness to mother and father figure. Inflammation was measured via high-sensitivity C-reactive protein (hs-CRP) in which levels were categorized based on clinical cut-points for risk of CVD (< 1 mg/L = low risk—reference, 1–3 mg/L = moderate risk, > 3–10 mg/L = high risk, > 10 mg/L = very high risk). Multivariable logistic regression was conducted accounting for the complex survey design and wave 4 control measures (e.g., body mass index, smoking, medications, acute illness, overall health, sociodemographic factors).		Women who reported they were somewhat/quite/very close to their mother figure (AOR = 0.48, 95% CI = 0.25, 0.92) and those who reported having no mother figure (AOR = 0.25, 95% CI = 0.08, 0.77) were less likely than women reporting being not very close/not close at all with their mother figure to have hs-CRP levels in the moderate- vs. low-risk category. No statistically significant associations were found between father-figure relationship and hs-CRP CVD risk category.			integration may exist.
Gender differences in loneliness, anger, depression, self-management	Theeke L, et al. Feb 2019, USA.	Cross-sectional study.	Instruments measured loneliness, anger, depression, and SMA (Self-Management Ability). Measures were	This study recruited 90 patients from a primary care center.	They were moderately lonely and men were lonelier than women (*p* < .01). Men and women did not differ on	This study presents new information that loneliness is prevalent in mid-	Including loneliness in behavioral health assessments in clinical practice	Future studies should examine longitudinal relationship between

ability and biomarkers of chronic illness in chronically ill mid-life adults in Appalachia			taken for BMI, blood pressure, and glucose. Descriptive and hierarchical multiple regression was used to test hypotheses.	Participants were majority female (68%), white (89%), married (52%), employed (66%), and impoverished (70%). Mean age 55.86, SD 5.5, range 45–64 years	anger, depressive symptoms, or SMA. Anger (5.64, SD 7.32) and depressive symptoms (M = 5.89, SD 5.54) were low. Loneliness positively correlated with anger ($r = 0.415$, $p < .01$) and depression ($r = 0.558$, $p < .01$), and inversely related to subscales of SMA ($p < .01$) [taking initiative (TI), investment behavior (IB), variety (V), multifunctionality (MF), self-efficacy (SE), and positive frame of mind (PM)]. After controlling for age, anger, and depression, loneliness was explanatory for poorer SMA (R squared change = 0.32, F change (1, 67) = 47.67, $p < .001$).	life adults in Appalachia and that loneliness is predictive of diminished self-management ability, even while controlling for the possible effects of age, anger, and depression.	and developing and studying the impact of interventions designed to target loneliness as a health problem will be imperative to continued understanding of loneliness and its impact on health.	psychosocial factors and biological markers in chronically ill adults and evaluate interventions that improve self-management as well it reduce loneliness and psychological distress.
Loneliness and biological responses to acute stress in people with Type	Hackett RA et al. Jun. 2019, UK.	Cross – sectional study.	The researchers assessed interleukin-6 (IL-6), interleukin-1 receptor antagonist (IL-1RA), monocyte	N=135, mean age =63.81 SD=± 6.93 years old, range 50–75 years.	Lonelier individuals had raised MCP-1 concentrations 75 min post stress independent of	Loneliness was associated with heightened inflammatory and stress responses	These findings suggest that social isolation may contribute to poorer	Future research is required to understand if such changes increase the risk of poorer

2 diabetes			chemoattractant protein-1 (MCP-1), and cortisol responses to acute stress in people with Type 2 diabetes. Loneliness was measured used the Revised UCLA Loneliness Scale. Loneliness was inversely associated with cortisol output post stress (B = -4.429, p = 0.019) independent of age, sex, education, marital status, body mass index, and smoking.	Male = 61.5% (majority)	covariates (B = 0.713, p = 0.022). No associations between loneliness and IL-6 or IL-1RA concentrations were detected. These results suggest that loneliness is associated with disturbances in stress responsivity in people with diabetes, and the impact of loneliness on health in people with diabetes may be mediated in part through dysregulation of inflammatory and neuroendocrine systems.	to acute stress, suggesting that social isolation may exacerbate physiological stress reactivity in individuals with type 2 diabetes.	physiological stress regulation and increased risk of diabetes-related complications at the population level.	outcomes in this population.
Additive effects of obesity and loneliness on C-reactive protein	Pavela G, et al. Nov. 2018, USA.	Cross – sectional study.	Obesity and loneliness are associated with C-reactive protein (CRP), a predictor of cardiovascular disease. It is unknown whether the co-presence of obesity and loneliness is associated with additional risk for clinically elevated CRP. The present study	N= 10.912 Men = 4.484, Women = 6.428. A large proportion of men and women participants were obese (41.6% and 42.6%, respectively).	Our primary outcome is high sensitivity CRP (hsCRP). Survey-weighted logistic regression models were used to test whether loneliness and BMI category are independent predictors of CRP after adjusting for demographics and other inflammatory-	The study found that obesity and loneliness have an additive association with higher CRP levels, suggesting that both metabolic and psychosocial factors may jointly contribute to	Given the high prevalence of both obesity and loneliness in the general population, their combined influence on inflammatory markers may represent an important	Future longitudinal studies are needed to determine causal pathways and to investigate whether interventions targeting weight management and social connectedness

			examines their independent and combined effects on elevated CRP in older adults. Data come from respondents who completed the 2008 and 2010 waves of the Health and Retirement Study. Loneliness was measured using an 11-item Loneliness Scale and body mass index was calculated from technician measured height and weight.		related covariates. In the fully adjusted model for men, obesity (OR = 2.36, p < 0.0001) was associated with increased odds of hsCRP >3.0. Among females, being overweight (OR = 1.75, p < 0.0001) or obese (OR = 4.01, p < 0.0001) were associated increased odds of hsCRP>3.0. Among both men and women, results from fully adjusted models indicated that loneliness was not associated with clinically elevated hsCRP (OR = 1.34, p = 0.0535; OR = 0.97, p = 0.6776, respectively).	systemic inflammation and increased cardiometabolic risk.	population-level contributor to chronic disease risk.	can reduce systemic inflammation.
Loneliness in middle age and biomarkers of systemic inflammation: Findings from Midlife in the United States	Nersesian PV, et al. Jul. 2018, USA.	Cross – Sectional Study.	This study used data from the Midlife in the US (MIDUS) survey Biomarker Project, which collected data on psychological, social, and physiological measures from a sample	This sample included the 927 participants who were 35-64 years at Biomarker Project data collection. The	29% of the sample reported feeling lonely most or some of the time. There was a positive significant relationship between loneliness and the three systemic	This study contributes to the body of research on loneliness among an understudied group: middle-aged adults,	Expanding understanding of the loneliness-inflammation relationship in middle age may inform policy on community-level	Further research might explore whether existing interventions influence loneliness' relationship with systemic

			of middle-aged adults. MIDUS collected baseline data from 1995-1996 and a follow-up survey was conducted from 2004-2006. The baseline Milwaukee sample of African Americans was collected in 2005-2006 and the biomarker database was collected in 2004-2009. Biomarkers were obtained from a fasting blood sample. Self-reported loneliness was categorized as feeling lonely or not feeling lonely. Hierarchical regressions examined the association between biomarkers of systemic inflammation (interleukin-6, fibrinogen, C-reactive protein) and feeling lonely, adjusted for covariates.	mean age for this sample was 52 years (*SD* =7.47). Female participants were 533 (57.50%).	inflammation biomarkers after controlling for covariates: interleukin-6 (n = 873) (b [se] = 0.07 [0.03], p = 0.014); fibrinogen (n = 867) (b [se] = 18.24 [7.12], p = 0.011); and C-reactive protein (n = 867) (b [se] = 0.08 [0.04], p = 0.035).	particularly those in the earlier part of middle age. These results also contribute to knowledge of the relationship between loneliness and a precursor of cardiovascular disease: systemic inflammation.	loneliness interventions and enhance individual care for lonely people in clinical settings. Reducing loneliness has the potential to improve quality of life and physical and mental health outcomes in middle-aged adults.	inflammation.
Social Relationships and Inflammatory Markers in the MIDUS Cohort:	Elliot AJ, et al. Jul. 2018, USA.	Cross-Sectional Study.	The researchers examined interactions of age and gender with structural and functional social network measures	Sample : middle-aged and older adults (N = 963) from the Midlife	Significant interactions involving age and gender showed that social support was associated with lower	These findings suggest that the quality and structure of social relationships may	Interventions aimed at strengthening social networks and social	Further work is needed to elucidate the reasons for the age and gender

The Role of Age and Gender Differences			in predicting interleukin-6 (IL-6) and C-reactive protein (CRP).	Development in the United States (MIDUS) biomarker project, English-speaking adults, ages 35 to 76 (mean age = 57,7 years old). 525 of the participants were female and 438 were male.	IL-6 in older women, whereas perceived positive relationships and social integration were related to lower IL-6 in both men and women of advanced age. Functional measures were associated with higher CRP in both men and women after adjustment for health conditions and behaviors, with some further variation by age.	influence inflammatory processes related to chronic disease risk, highlighting social support as a potential protective factor in the clinical management of inflammation and age-related health conditions.	support, particularly among older adults, could potentially contribute to reduce chronic inflammation and improve health outcomes at population level. Additionally, social integration and supportive relationships may have important implications for population health.	differences observed here and to understand implications for interventions seeking to leverage social relationships.
Social Relationships, Inflammation, and Cancer Survival	Boen CE, et al. May 2018, USA.	Cross-sectional study.	Social factor and gene expression data were collected from individuals in the Multi-Ethnic Study of Atherosclerosis (MESA). Inflammation-related genes were identified from the Gene Ontology database. The associations between social factors and gene expression were first assessed using the Global Analysis of Covariance (Global	N = 1,264, they used a random sample of individuals from four of the MESA study sites in Exam 5. From this sample: Non-Hispanic Black (n=272), Hispanics (n=402), Non-Hispanic White (n=590). The	Loneliness (p=0.003), chronic burden (p=0.002), and major or lifetime discrimination (p=0.045) were significantly associated with global expression of the chronic inflammatory gene set. Of the 20 transcripts that comprise this gene set, elastic net selected 12 transcripts for loneliness, 8 for chronic burden, and 3 for major	This study highlights gene expression as a biological mechanism through which social factors may affect inflammation.	These finding suggest that social environment can contribute to biological pathways linked to chronic disease. Addressing social determinants of health could therefore have important implications for reducing	Future studies should explore casual mechanism linking social relationship with gene expression and inflammation. They should also examine how social interventions may influence biological pathways and cancer outcomes.

			ANCOVA) gene set enrichment test. When the global test was significant, linear regression and elastic net penalized regression tests were employed to identify the individual gene transcripts within each gene set associated with the social factor.	mean age of the sample was 69.6 years old and 51% were women.	or lifetime discrimination. Major or lifetime discrimination was also associated with the inflammatory response (p=0.029), regulation of the inflammatory response (p=0.041), and immune response (p=0.025) gene sets in global analyses, and 53, 136, and 26 transcripts were selected via elastic net for these gene sets respectively. There were no significant associations in linear regression analyses after adjustment for multiple testing.		inflammation-related disease risk at the population level.	
Prospective associations of social isolation and loneliness with poor sleep quality in older adults	Yu B, et al. Mar. 2018, Taiwan.	Longitudinal analysis. Observational study.	Poisson regression models were conducted to examine the association of social isolation and/or loneliness with sleep quality at follow-up after adjusting for multiple confounding variables.	Participants came from the Social Environment and Biomarkers of Aging Study in Taiwan collected in 2000 and 2006, involving a cohort of 639	Univariate analysis showed that sleep quality was inversely associated with both social isolation and loneliness. After demographic, health, cognitive factors, and depressive symptoms were controlled in multivariable analysis,	These findings suggest that social isolation independently contributes to the poor sleep quality in later life, while loneliness does not appear to confound the effect of isolation.	Social isolation and loneliness seem to have distinct pathways in affecting the sleep quality of older adults. It is possible therefore that older adults' sleep might benefit from social	Future studies that incorporate both subjective and objective instruments (e.g., actigraphy, polysomnography) are desirable.

				participants (mean age = 66.14, SD= 7.26). Of the cohort 56.5% was male, and 77.1% was married.	social isolation at the baseline still predicted poor sleep quality 6 years later (incident rate ratio, IRR 1.14; 95% CI 1.04-1.24; p < 0.01), while the association between loneliness and sleep quality was no longer significant (IRR 1.08; 95% CI 0.94-1.23; p = 0.27). The results were unchanged when participants who had poor sleep quality at the baseline were excluded from the analysis.		interventions. Programs that aim to foster social connections may have effects not only on wellbeing but also on older adults' sleep quality.	
Objectively Measured Social Integration Is Associated with an Immune Risk Phenotype Following Marital Separation	Hasselmo K, Feb. 2018, USA.	Longitudinal Study.	The researchers collected four measures of immune functioning- interleukin-6, C-reactive protein, and antibody titers to latent cytomegalovirus and Epstein-Barr virus-that were combined to yield a viral-Immune Risk Profile. To assess how variability in social integration is associated with immunological	This study involved 49 adults n=15 men, mean age =44.08, SD= 11.16 who reported a recent marital separation (mean months since separation = 3.67 months, SD= 2.61)	They found that objectively measured social behaviors are associated with concurrent viral-Immune Risk Profile scores over and above the effects of psychological distress and that psychological distress may be linked to biomarkers of immune health through social integration.	This research expands current knowledge of biomarkers of immune health after divorce and separation and includes a new methodology for objective measures of social engagement.	These results illustrate a pathway linking psychological stress associated with divorce may to immune health and suggest future research on whether targeting these social behaviors can improve health outcomes after	Further study using a larger sample and examining preregistered hypotheses may provide increased precision and confidence in the current study's results.

			correlates following the end of a marriage, they incorporated observational ecological momentary assessment data using a novel methodology (the Electronically Activated Recorder).				marital separation.	
Blood biomarkers of Hikikomori, a severe social withdrawal syndrome	Hayakawa K. et al. Feb. 2018, Japan.	Case – Control Study	Study 1: Kyushu University campus was selected as a place for recruiting volunteers. Posters and flyers were used, targeting students and staff as healthy volunteers. After obtaining informed consent, their socio-demographic information was collected. A doctor asked all participants regarding their physical condition. All participants completed a series of self-rated questionnaires, played a trust game, and provided a non-fasting venous blood sample. Measured blood biomarkers: serum HDL-C, LDL-C, serum total bilirubin, UA, hsCRP,	In Study 1, a total of 101 young non-clinical volunteers were recruited. 46 participants were male and 55 were female.<br><br>In study 2, a total of 133 people were included. 63 of the participants were male and 70 were female. 55 of the total were with hikikomori and 78 were in healthy control.	Study 1 showed significant relationships between avoidant personality traits, blood biomarkers, and behavioral characteristics assessed by the trust game, and psychological features (loneliness, social anxiety, trust, and Internet addiction) in healthy volunteers. Candidate blood biomarkers of avoidant personality traits were identified as follows: HDL-C and UA in men, and FDP and hsCRP in women. The SEM (structural equation modeling) analysis suggests that avoidant personality traits	Hikikomori is connected with specific biological changes, metabolic and inflammatory biomarkers, indicating that severe social withdrawal has a systematic biological impact apart from psychological dimension.	The constant increase of hikikomori is not only consider a social or a psychological problem. It also seems to have an impact on physical health and morbidity as well as a biological impact that effects public health.	Further studies are needed to validate the findings and elucidate the role of biomarkers in avoidant personality and hikikomori.

			and plasma.<br><br>Study 2: Sample conducted in Fukuoka (Japan). Study recruitment occurred in six psychiatric hospitals and clinics affiliated with the Kyushu University, and two community mental health centers. They included all individuals who met criteria for currently having hikikomori based on a structured diagnostic interview designed for this project. All participants completed a series of self-rated questionnaires same as conducted in Study 1, underwent a structured interview for hikikomori with a study psychiatrist or psychologist, and provided a non-fasting venous blood sample.		exacerbating psychological isolation in both sexes were indirectly associated with behavioural uncooperativeness in **men and** directly induced distrust of others in women. Study 2 revealed that individuals with hikikomori had higher avoidant personality scores in both sexes, lower serum UA levels in men, and lower serum HDL-C levels in women.			
Loneliness 5 years ante-mortem is	Canli T, et al.	Longitudinal	Researchers analyzed loneliness data collected	N=181, age at death in years	Results are based on a pre-ranked Gene Set	Loneliness, associated with	Given the high prevalence of	These gene sets constitute

associated with disease-related differential gene expression in postmortem dorsolateral prefrontal cortex	Jan. 2018, USA.	study.	on average 5 years ante-mortem and RNA gene expression at death in postmortem dorsolateral prefrontal cortex (DLPFC) from participants in the Rush Memory and Aging Project (MAP), a longitudinal, prospective cohort study of common chronic conditions of aging. Our analytic protocol controlled for biographical variables (age, sex, education), psychological and health variables (depressive symptoms, interval between assessment and autopsy, slope of cognitive decline, AD pathology, presence of infarcts) and RNA integrity.	mean, SD = 89.5, 6.2. Women = 119 (65.8%).	Enrichment Analysis (GSEA) at FDR-corrected q-values <0.05, using these collections from the Molecular Signatures Database: Hallmarks, Canonical, Gene Ontology (GO), Chemical and Genetic Perturbations, Immunologic Signatures, Oncogenic Signatures and Cancer Modules. We now report on 337 up-regulated and 43 down-regulated gene sets, among which the most significant ones were associated with Alzheimer's disease, psychiatric illness, immune dysfunction, and cancer.	disease-related changes in gene expression in the dorsolateral prefrontal cortex, indicating neurobiological pathways through which loneliness influence brain health and vulnerability to neuropsychiatric disorders.	loneliness in older populations, these findings highlight loneliness as a potential population-level risk factor for biological changes in the brain that may contribute to cognitive decline and mental health disorders.	attractive targets for future studies into the molecular mechanisms by which loneliness exacerbates a wide range of neurodegenerative, psychiatric, and somatic illnesses.
Potential biological pathways linking Type-D personality and poor health: A cross-sectional investigation	Jandackova VK, et al. Apr. 2017, Germany.	cross-sectional study.	Data from the respondents were available for analysis. Persons with Type-D (negative affect and social isolation score ≥10) were contrasted with those without Type-	A total of 646 respondents (Mean age 41.6±11.5, 12,2% were women) were available for analysis. The	Persons with Type-D had higher HbA1c, FPG, and fibrinogen, and lower nighttime HRV than those without Type-D, suggesting worse glycemic control, systemic inflammation	Findings provide some of the first evidence for multiple possible biological and behavioral pathways between Type-D	The findings suggest that Type-D personality and social isolation may be linked to unfavorable cardiometabolic	Future longitudinal studies should investigate causal pathways between Type-D personality, social isolation, and biological markers,

			D. Measures of plasma fibrinogen levels, white blood cell count, high sensitivity C-reactive protein, fasting plasma glucose (FPG), cholesterol, high-density and low-density lipoprotein, glycated hemoglobin (HbA1c), creatinine, triglycerides, and albumin were derived from fasting blood samples. Urine norepinephrine and free cortisol were determined by high-performance liquid chromatography. Time-domain heart rate variability (HRV) measures were calculated for the 24hr recording period and for nighttime separately.	study population comprised apparently healthy employees of an airplane manufacturing plant in Southern Germany.	and poorer autonomic nervous system modulation in Type-D persons. In addition, those with Type-D reported less social support and greater sleep difficulties while no group differences were observed for alcohol and cigarette consumption, physical activity and body mass index.	personality and increased morbidity and mortality.	and inflammatory biomarkers, highlighting their potential role as risk factors for poorer population health.	and examine whether psychosocial interventions can improve health outcomes.
Positive Affect, Social Connectedness, and Healthy Biomarkers in Japan and the U.S.	Yoo J et al. Dec. 2016, Japan and USA.	Cross-sectional study.	The present study investigated the interplay of positive affect, social connectedness, and health using large probability samples of Japanese and U.S. adults. Health was measured objectively	The present analyses include 1.043 participants for whom physical health data were available (472 males, 571 females, Mean	Lower levels of both biomarkers (i.e., less healthy biomarker profile) were found among those in Japan who reported high positive affect in combination with low social connectedness.	Findings underscored the interactive influence of both factors in Japan, via a pattern showing worse biological health among those with	The findings highlight cultural variations in the health implications of how positive affect and social connectedness come together.	Future studies should examine the mechanisms linking positive affect and social connectedness with biological health and explore cultural differences

			with biomarkers that represent restorative functioning: HDL (high-density lipid) and DHEA-S (dehydroepiandrosterone-sulfate).	age = 55.24 years). For MIDJA (Midlife in Japan), N = 382, 168 males, 214 females, M = 54.24 years.	In the U.S, the general pattern was that those with greater positive affect showed healthier HDL levels regardless of social connectedness.	low social connectedness combined with high positive affect. Such interplay was not present in the U.S., where only positive affect was shown to predict better HDL cholesterol.		in this relationship across populations.
Daily social interactions, close relationships, and systemic inflammation in two samples: Healthy middle-aged and older adults	Bajaj A. et al. Nov. 2016, USA	Observational study.	Global social support and integration were measured using the Interpersonal Support Evaluation List (ISEL) and the Social Network Index (SNI), respectively, in older adults from the Pittsburgh Healthy Heart Project (PHHP), and in middle-aged adults from the Adult Health and Behavior Project–II (AHAB-II). Using time-sampled EMA (Ecological Momentary Assessment), they assessed the proportion of the day spent in positive and negative social interactions.	Participants included 306 men and women enrolled in the Pittsburgh Healthy Heart Project (PHHP), a prospective study of healthy, community-dwelling adults aged 50–70 years, and 419 men and women enrolled in the Adult Health and Behavior Project – Phase 2 (AHAB-II), aged 30–54.	Global measures of support and integration did not associate with inflammation in either sample. In older adults, relative frequency of total positive interactions, those with close others (i.e. spouse, friends, family), and those with coworkers predicted lower concentrations of IL-6 in fully adjusted models, accounting for age, sex, race, education, BMI, smoking and alcohol. In middle-aged adults, relative frequency of positive interactions	Positive every day interaction is related to lower inflammation levels.	The quality of everyday social relationship effects inflammation levels and increases the risk of chronic diseases.	Future research may consider exploring additional mechanistic processes that may account for the association between social constructs and systemic inflammation. These processes may include regulation of daily affect, frequency of engagement in health behaviors, and modulation of biological stress responses, all of

			Systemic markers of inflammation were interleukin (IL)-6 and C-reactive protein (CRP).		with close others was also inversely associated with IL-6 level and relative frequency of negative marital interactions was unexpectedly inversely associated with CRP level.			which are implicated in chronic, systemic inflammation.
Social connectedness is associated with fibrinogen level in a human social network	Kim DA et al. Aug. 2016, USA.	Cross-Sectional study.	The Framingham Offspring Study (FOS) began in 1971 with the recruitment men and women who were offspring of the original FHS (Framingham Heart Study) participants, or these offspring's spouses. In this study data used from Wave 5 of the FOS, which ran from 23 January 1991 to 29 June 1995, and which included fibrinogen values for the greatest number of individuals. They first characterize fibrinogen as a function of basic demographic and health variables: age, sex, body mass index (BMI), smoking status, income and educational attainment.	*N* = 3568 individuals had fibrinogen measurements taken during the study period.	In this study findings show that greater indegree, a sociocentric measure of friendship and familial ties identified by a subject's social connections rather than by the subject, predicts significantly lower concentrations of fibrinogen (a biomarker of inflammation and cardiac risk), after adjusting for demographics, education, medical history and known predictors of cardiac risk. The association between fibrinogen and social isolation, as measured by low indegree, is comparable to the	Low social connectedness was associated with higher fibrinogen levels, independent of demographic characteristic and established cardiovascular risk factors, suggesting activation of an inflammatory pathway. The magnitude of the association was comparable to that of smoking, highlighting social isolation as a clinically significant and independent risk factor for	Given the high prevalence of social isolation in the general population, the potential attributable burden of disease is substantial. These findings support social integration as a potential target for public health innervations aimed at reducing inflammation and cardiovascular risk at a population level.	Future work will benefit from longitudinal measurements of both network position and inflammatory biomarkers across the life course.

			Then examine the bivariate relationships between fibrinogen levels and indegree, and fibrinogen and outdegree.		effect of smoking, and greater than that of low education, a conventional measure of socioeconomic disadvantage. By contrast, outdegree, which reflects an individual's perceived connectedness, displays a significantly weaker association with fibrinogen concentrations.	cardiovascular disease.		
Loneliness, Depression, and Inflammation: Evidence from the Multi-Ethnic Study of Atherosclerosis	Mezuk B, et al. Jul. 2016, USA.	Cross – sectional study.	Data come from a subset of the Multi-Ethnic Study of Atherosclerosis. Loneliness was measured by three items derived from the UCLA Loneliness Scale. The association between loneliness and C-reactive protein (CRP) and fibrinogen was assessed using multivariable linear regression analyses. Models were adjusted for demographic and health characteristics.	N = 441, mean age = 63.4, SD = ± 8.9. Female participants were 237 (53.7%).	Approximately 50% of participants reported that they hardly ever felt lonely and 17.2% felt highly lonely. Individuals who were unmarried/unpartnered or with higher depressive symptoms were more likely to report being highly lonely. There was no relationship between perceived loneliness and ln(CRP) (β = -0.051, p = 0.239) adjusting for demographic and health characteristics.	These findings add to the growing body of literature aimed at understanding the mechanisms by which factors such as social isolation and perceived loneliness may influence health in later life.	Emerging research indicates that low levels of loneliness, much like high levels of social support, may buffer the effect of stressors on health.	Future studies should continue to explore both the direct and indirect pathways that may explain the observed associations between loneliness and health in later life.

					Loneliness was inversely associated with ln(fibrinogen) (β = -0.091, p = 0.040), although the absolute magnitude of this relationship was small. These results indicate that loneliness is not positively associated with fibrinogen or CRP among relatively healthy middle-aged adults.			
Social relationships and physiological determinants of longevity across the human life span	Yang YC, et al. Jan. 2016, USA.	Cross – sectional study	Drawing on data from four nationally representative longitudinal samples of the US population, they implemented an innovative life course design to assess the prospective association of both structural and functional dimensions of social relationships (social integration, social support, and social strain) with objectively measured biomarkers of physical health (C-reactive protein, systolic	-N = 7.889 (Add health - he National Longitudinal Study of Adolescent to Adult Health), mean age =28.2 years old, range 24-32, female participants= 48.4% of the sample.<br><br>-N = 863 (MINDUS - National Survey of Midlife	They found that a higher degree of social integration was associated with lower risk of physiological dysregulation in a dose-response manner in both early and later life. Conversely, lack of social connections was associated with vastly elevated risk in specific life stages. For example, social isolation increased the risk of inflammation by the same magnitude as physical inactivity in	Those findings suggest the early emergence and continuity of the physiological impacts of social relationships across the life course. They also suggest physiological vulnerabilities to social stress that may be specific to life course stages and relationship stressors. Disrupting various	In adolescence, social integration was more pertinent to physical health than social support as perceived by individuals. This life course pattern of variation in the relative importance of social integration and support to physiological functioning was previously	Future research suggests examining the mechanisms through which social relationships influence biological systems, to conduct life-course research exploring how social relationships at different stages of life affect health, to integrate social and biological data including biomarkers, and to

			and diastolic blood pressure, waist circumference, and body mass index) within each life stage, including adolescence and young, middle, and late adulthood, and compare such associations across life stages.	Development in the United States), mean age =44 years old range =25-64, female participants =55.5% of the sample.<br><br>~N = 4.323 (HRS - Health and Retirement Study) mean age =67,3 years old, range =50-98, female participants =54.9% of the sample.<br><br>~N = 1.571 (NSHAP - National Social Life, Health, and Aging Project), mean age =67.3 years old range =57-85, female participants =53.3% of the sample.	adolescence, and the effect of social isolation on hypertension exceeded that of clinical risk factors such as diabetes in old age. Analyses of multiple dimensions of social relationships within multiple samples across the life course produced consistent and robust associations with health. Physiological impacts of structural and functional dimensions of social relationships emerge uniquely in adolescence and midlife and persist into old age.	social relation deficits and physiological risk connections could directly arrest early progression toward chronic diseases and delay disease onset or lessen the disease burden in late life. The findings, therefore, provide a strong scientific basis for effective prevention and intervention that will lead to further improvement in life expectancy.	unknown and can have new practical import for policy intervention.	improve understanding of the timing and duration of the effects of social relationships on health.
Trait sensitivity to social	Moieni M et al. Dec. 2015, USA	Randomized controlled trail	The impact of sensitivity to social disconnection	N = 155, healthy adults, 69	Those more sensitive to social disconnection	People high sensitivity to	Social disconnection is a	Present findings generally support

disconnection enhances pro-inflammatory responses to a randomized controlled trial of endotoxin		(double-blind, placebo - controlled design).	was investigated (a composite score comprised of loneliness, anxious attachment, fear of negative evaluation, and rejection sensitivity) on pro-inflammatory cytokines and gene expression in response to endotoxin, an inflammatory challenge, versus a placebo. 90 min. after arrival each participant was randomly assigned to receive either low-dose endotoxin or placebo, administered by the nurse as an intravenous bolus. 7 blood draws and vital signs were collected, first one before the injection and then approximately every hour for the next six hours. TFN- a (Trochanteric Fixation Nail-Advanced) and IL-6 was measured to investigate the pro-inflammatory response.	females, mean age = 24.2, SD = ± 6.6	showed increased pro-inflammatory responses (i.e., increased levels of tumor necrosis factor-alpha and interleukin-6) to endotoxin, as well as up-regulation of multiple genes related to inflammation. Bioinformatics analyses revealed that those in the endotoxin group who are more sensitive to social disconnection exhibited a Conserved Transcriptional Response to Adversity (CTRA) regulatory profile, involving up-regulation of beta-adrenergic and pro-inflammatory transcription control pathways and down-regulation of antiviral transcription factors in response to endotoxin. Those more sensitive to threats to their social relationships may be primed to exhibit heightened inflammatory	social disconnection showed higher numbers of pro-inflammatory cytokines (TNF-α, IL-6) and more intense inflammatory response from those with lower sensitivity. This way it demonstrates social disconnection's sensitivity as a biological risk factor for inflammation.	threat to public health. It contributes to the overall inflammatory burden of the population and increased morbidity. At the same time social disconnection's prevention is public health's main goal, in order to prevent chronic inflammatory responses.	the notion that there is a relationship between social disconnection and inflammation that warrants future research.

					responses to immunological insults, which may ultimately lead to chronic inflammatory-related illnesses.			
Social network diversity and white matter microstructural integrity in humans	Molesworth T, et al. Sep. 2015, USA.	Cross-sectional study.	In healthy adults, the researchers tested the links between social integration and measures of white matter microstructure using diffusion tensor imaging. Across the brain, there was a predominantly positive association between a measure of white matter integrity, fractional anisotropy (FA), and social network diversity. This association was particularly strong in a region near the anterior corpus callosum and driven by a negative association with the radial component of the diffusion signal. This callosal region contained projections between bilateral prefrontal cortices, as well as	Participants were 155 community-dwelling adults (78 men) who were recruited via mass mailings to residents of Allegheny County, Pennsylvania (USA).	FA within this region was weakly associated with circulating levels of the inflammatory cytokine interleukin-6 (IL-6), but IL-6 did not mediate the social network and FA relationship. Finally, variation in FA indirectly mediated the relationship between social network diversity and intrinsic functional connectivity of medial corticostriatal pathways.	The current findings suggest that, like in rodents, reduced social network structure in humans is linked to the health of myelin in the brain.	The findings suggest that greater diversity in social networks may be linked to better brain structural integrity, highlighting the potential importance of social engagement for maintaining brain health in the general population.	Future longitudinal studies are needed to determine causal relationships and to explore the mechanisms linking social network diversity with brain structure and cognitive health.

			cingulum and corticostriatal pathways.					
Sleep Disturbance and Longitudinal Risk of Inflammation: Moderating Influences of Social Integration and Social Isolation in the Coronary Artery Risk Development in Young Adults (CARDIA) Study	Cho HJ, et al. May 2015, USA.	Longitudinal study.	This study examined the longitudinal association between sleep disturbance and systemic inflammation, and the moderating effects of social isolation on this association. The CARDIA (Coronary Artery Risk Development in Young Adults) study is a population-based longitudinal study conducted in four US cities. Sleep disturbance – i.e., insomnia complaints and short sleep duration – was assessed (2000-2001). Circulating C-reactive protein (CRP) was measured at baseline and follow-up (2005-2006). Interleukin-6 (IL-6) and subjective and objective social isolation (i.e., feelings of social isolation and social network size) were measured at follow-up.	N = 2.962 participants African American and white adults. Age (33-45 years) mean age = 40.3 (SD = ± 3.6). 43.9% of the sample were male.	Sleep disturbance was a significant predictor of inflammation five years later after full adjustment for covariates (adjusted betas: 0.048, P=0.012 for CRP; 0.047, P=0.017 for IL-6). Further adjustment for baseline CRP revealed that sleep disturbance also impacted the longitudinal change in CRP levels over five years (adjusted beta: 0.044, P=0.013). Subjective social isolation was a significant moderator of this association between sleep disturbance and CRP (adjusted beta 0.131, P=0.002). Sleep disturbance was associated with heightened systemic inflammation in a general population over a five-year follow-up,	The current study provides evidence on the longitudinal association between sleep disturbance and heightened systemic inflammation and also on the moderating influences of subjective and objective social isolation on this association, extending the findings of prior cross-sectional studies.	The current data suggest that addressing sleep disturbances, which may be amenable to clinical interventions, might be a potential avenue for reducing inflammation; and this may be particularly important in those who perceive themselves as being socially isolated.	Future studies should explore longitudinal mechanisms linking sleep, stress physiology, and social relationships with inflammation and also investigate whether interventions targeting sleep quality and social connectedness can reduce inflammatory burden and cardiovascular risk of inflammation.

					and this association was significantly stronger in those who reported feelings of social isolation. Clinical interventions targeting sleep disturbances may be a potential avenue for reducing inflammation, particularly in individuals who feel socially isolated.			
The Association Between Loneliness and Inflammation: Findings From an Older Adult Sample	Van Bogart K, et al. 2022, USA.	Longitudinal observational (trait and ecological momentary assessment).	222 community-dwelling older adults completed trait loneliness questionnaires and ecological momentary assessment (EMA) of loneliness, with blood sampling for CRP and a cytokine panel.	N = 222 older adults.	Both trait loneliness (β=0.16, p=0.02) and momentary EMA loneliness (β=0.15, p=0.03) were associated with higher CRP; no associations were observed for the broader cytokine panel.	CRP may be a sensitive marker of day-to-day loneliness fluctuations in older adults.	Supports feasibility of tracking inflammatory responses to loneliness in daily life.	Replication in larger, diverse samples with repeated CRP and cytokine measurement.
Social isolation, loneliness, and inflammation: A multi-cohort investigation in early and mid-adulthood	Matthews T, et al. 2024, Denmark/UK/USA .	Longitudinal multi-cohort analysis.	Three longitudinal cohorts (TRIAGE, Dunedin, E-Risk) with measures of living alone, childhood social isolation, and loneliness, and inflammatory markers (suPAR, CRP, IL-6).	N = 6,144 + 881 + 1,448 across three cohorts.	Living alone was associated with higher suPAR but not CRP or IL-6; childhood social isolation predicted higher adult suPAR after covariate adjustment; loneliness at ages 38/45 predicted suPAR at age 45; age-18 loneliness was not associated (E-Risk).	suPAR may capture chronic inflammatory correlates of isolation more sensitively than CRP or IL-6.	Life-course social isolation is embedded in chronic inflammation measurable in mid-adulthood.	Test suPAR as a monitoring biomarker in social-prescribing and intervention studies.

Loneliness, epigenetic age acceleration, and chronic health conditions	Freilich CD, et al. 2024, USA.	Longitudinal observational (MIDUS).	1,310 MIDUS participants (longitudinal subsample n=445) with loneliness measures and DNA methylation-based epigenetic age estimators (Horvath, DunedinPACE, GrimAge).	N = 1,310 (longitudinal subsample n = 445).	Loneliness was weakly associated with epigenetic age acceleration (β≈0.08–0.11); a loneliness × DunedinPACE interaction predicted chronic-condition burden (β=0.09, p=0.009).	Epigenetic ageing may link loneliness to accumulating chronic disease.	Adds molecular evidence that loneliness relates to biological ageing in midlife and older adults.	Larger longitudinal samples with repeated epigenetic measures.
Associations Between Loneliness, Epigenetic Aging, and Multimorbidity Through Older Adulthood	Freilich CD, et al. 2024, USA.	Longitudinal cohort (Health and Retirement Study).	4,018 HRS participants with loneliness measures and GrimAge epigenetic ageing; mediation of the loneliness–multimorbidity association was tested.	N = 4,018 older adults.	Loneliness predicted GrimAge acceleration; epigenetic ageing partly mediated the loneliness–multimorbidity association (p=0.003).	Epigenetic ageing is a plausible pathway from loneliness to multimorbidity.	Identifies a measurable molecular mediator relevant to geriatric multimorbidity.	Interventional tests of whether reducing loneliness slows epigenetic ageing.
Protein signatures associated with loneliness and social isolation: Plasma proteome analyses in the English Longitudinal Study of Ageing, with causal evidence from Mendelian randomization	Gong J, et al. 2025, UK.	Prospective cohort with proteomics and Mendelian randomization (ELSA).	3,262 ELSA participants (mean age 63.4 years, 55% women) with wave-4 plasma proteomics (276 proteins); two-sample MR tested causal effects of social contact on protein levels.	N = 3,262 adults aged 50+.	11 proteins were associated with social isolation after minimal adjustment and 3 (TNFRSF10A, TNFRSF11A, HAOX1) after full adjustment; MR indicated lower in-person contact causally increased TNFRSF10A/TRAIL-R2, TNFRSF11A, and KIM1 and decreased NEFL.	Proteomic signatures point to specific molecular pathways (e.g., TNF receptor family) linking isolation to disease.	Replicates and extends UK Biobank proteomic findings in an independent cohort.	Validate protein signatures as intervention targets or monitoring markers.
Changes in Loneliness, BDNF, and Biological Aging Predict Trajectories in a Blood-Based Epigenetic Measure of Cortical Aging: A	Simons RL, et al. 2023, USA.	Longitudinal cohort.	694 older Black Americans with repeated loneliness measures, BDNF methylation, and a blood-based epigenetic measure of cortical ageing (CC-Bd); robustness to	N = 694 older Black Americans.	Increases in loneliness and BDNF methylation predicted accelerated cortical ageing, robust to adjustment for DunedinPACE and Pace of Aging.	Links loneliness to brain-relevant biological ageing in an underrepresented population.	Extends epigenetic-ageing findings to older Black Americans.	Examine whether loneliness interventions affect cortical-ageing biomarkers.

Study of Older Black Americans			DunedinPACE and Pace of Aging was tested.					
Do Loneliness and Per Capita Income Combine to Increase the Pace of Biological Aging for Black Adults across Late Middle Age?	Beach SRH, et al. 2022, USA.	Longitudinal cohort (11-year follow-up).	685 late-middle-aged Black adults with loneliness, per-capita income, and DunedinPACE pace-of-ageing measures; models adjusted for cell-type composition, smoking, and alcohol use.	N = 685 late-middle-aged Black adults.	Loneliness combined with lower income predicted increased DunedinPACE over 11 years, robust to cell-type composition, smoking, and alcohol use.	Socioeconomic context moderates the biological embedding of loneliness.	Highlights interaction of social and economic adversity in accelerated ageing.	Test combined social and economic interventions on pace of ageing.

Supplementary Table S1. Database-specific search strings

PubMed / Medline

(

"Social Isolation"[Mesh] OR "Loneliness"[Mesh]

OR social isolation[tiab] OR loneliness[tiab]

OR "social engagement"[tiab] OR "social participation"[tiab]

OR "social connectedness"[tiab] OR "social integration"[tiab]

)

AND

(

"Inflammation"[Mesh] OR inflammation[tiab]

OR "Biomarkers"[Mesh] OR biomarker*[tiab]

OR "Inflammation Mediators"[Mesh] OR "inflammatory marker*"[tiab]

OR cytokine*[tiab] OR "C-Reactive Protein"[Mesh] OR CRP[tiab]

OR interleukin*[tiab] OR IL-6[tiab] OR TNF[tiab] OR fibrinogen[tiab]

)

AND (humans[Mesh])

AND english[lang]

AND ("2015/01/01"[dp] : "2025/12/31"[dp])

Scopus

TITLE-ABS-KEY(

("social isolation" OR loneliness OR "social engagement" OR "social participation"

OR "social connectedness" OR "social integration")

AND

(inflammation OR "inflammatory marker*" OR biomarker* OR cytokine*

OR "c-reactive protein" OR CRP OR interleukin* OR "IL-6" OR TNF OR fibrinogen)

)

AND PUBYEAR > 2014 AND PUBYEAR < 2026

AND (LIMIT-TO(LANGUAGE, "English"))

ScienceDirect

("social isolation" OR "social engagement" OR “loneliness”)AND(inflammation OR "inflammatory marker" OR "c-reactive protein" OR CRP)

Targeted supplementary search (executed in Europe PMC, October 2026 and PsychInfo, October 2026)

Europe PMC

(TITLE_ABS:"social isolation" OR TITLE_ABS:"loneliness" OR TITLE_ABS:"social connectedness" OR TITLE_ABS:"social integration" OR TITLE_ABS:"social engagement" OR TITLE_ABS:"social participation") AND (TITLE_ABS:"epigenetic" OR TITLE_ABS:"DNA methylation" OR TITLE_ABS:"methylation" OR TITLE_ABS:"proteomic" OR TITLE_ABS:"proteomics" OR TITLE_ABS:"metabolomic" OR TITLE_ABS:"metabolomics" OR TITLE_ABS:"suPAR" OR TITLE_ABS:"urokinase plasminogen activator receptor") AND FIRST_PDATE:[2015-01-01 TO 2025-12-31] AND LANG:"eng"

Web of Science equivalent string (for reproduction):

TS=("social isolation" OR loneliness OR "social connectedness" OR "social integration" OR "social engagement" OR "social participation") AND TS=(epigenetic OR "DNA methylation" OR methylation OR proteomic* OR metabolomic* OR suPAR OR "urokinase plasminogen activator receptor"), 2015–2025, English, articles.

PsychINFO (via Ovid)

(

(social isolation or loneliness or social connectedness or social integration or social engagement or social participation).ti.

and

(epigenetic or DNA methylation or methylation or proteomic* or metabolomic* or suPAR or urokinase plasminogen activator receptor).ti,ab.

)

or

(

(social isolation or loneliness or social connectedness or social integration or social engagement or social participation).ti,ab.

and

(epigenetic or DNA methylation or methylation or proteomic* or metabolomic* or suPAR or urokinase plasminogen activator receptor).ti.

Supplementary Table S2. Detailed study-level data extraction matrix for key included studies

Axis	Study (author/year)	Country	Design	Setting / recruitment	Age range / mean	Sample size	Key inclusion/exclusion criteria	Psychosocial exposure(s) and measures	Biomarker(s) and medium	Outcome domain(s)	Analysis approach	Main quantitative findings (compact)
1	Koh-Bell et al., 2021	USA	Cross-sectional with mortality follow-up (NHANES)	NHANES 1988–1994 women; nationally representative	40–89 years; mean 54	3,360	Women with complete SNI and biomarker data; excludes missing exposure/outcome data	Social isolation via Social Network Index (most, very, somewhat, not isolated); categories based on marital status, contacts, group participation	Fibrinogen and CRP (blood)	Cancer mortality; inflammatory profile	Chi-square tests; multivariable Cox regression	Fibrinogen ↑ with greater isolation (p=0.003); CRP–isolation null (p=0.52); obesity HR=1.56 (95% CI 1.11–2.18), smoking HR=4.42 (95% CI 2.84–6.88), high physical activity HR=0.67 (95% CI 0.51–0.87) for cancer mortality; isolation not independently associated with cancer mortality (p=0.54) (Koh-Bell et al., 2021)
1	Zilioli et al., 2021	USA	Cross-sectional cohort	MIDUS Refresher biomarker and daily diary subsample	25–75 years; mean 47.7	314	Adults completing phone interview, questionnaires, biomarker protocol and daily diary; exclusions not detailed	Living status (alone vs with others); frequency of social contacts; loneliness scale	CRP, IL-6 (blood); salivary cortisol (multiple samples over a day)	Endocrine (diurnal cortisol pattern); systemic inflammation	Regression models examining isolation and loneliness in relation to cortisol and inflammatory	Living alone associated with flatter diurnal cortisol slope and higher CRP; loneliness associated with higher IL-6

											markers	independent of isolation; loneliness did not mediate isolation–cortisol/CRP effects; age did not moderate relationships (Zilioli et al., 2021)
2	MacAulay et al., 2024	USA	Cross-sectional	MIDUS Biomarker Project; national adult sample	26–78 years; mean 52.53	822	Adults with biomarker and psychosocial data; exclusions for incomplete measures	Loneliness (standard scale); BMI; number of friends; multimorbidity; depression history and symptoms	IL-6 (blood)	Gait speed (walking time); systemic inflammation	Regression and serial mediation using PROCESS macro	Age, sex, BMI, waist-hip ratio and loneliness (but not multimorbidity, depression, number of friends) contributed to IL-6 variance; serial mediation supported chained effect of loneliness on walking time via BMI and IL-6; specific indirect paths via BMI and IL-6 significant (MacAulay et al., 2024)
2	Vasan et al., 2024	Australia	Cross-sectional	Young adults recruited for cardiovascular testing	18–35 years	66	Young adults without known CVD; completed psychological questionnaires	Loneliness; depressive symptoms; social anxiety (questionnaire	Pulse transit time (arterial stiffness); endothelial	Early cardiovascular disease markers	Linear regression models adjusting for psychosocial	Higher loneliness associated with shorter pulse transit time (β≈−0.70,

							and physiological tests	s)	function indices (vascular tests)		and physiological covariates	p=0.002), indicating increased arterial stiffness; depression associated with increased endothelial dysfunction (β≈0.43, p=0.04); loneliness and depression may affect vascular function through different mechanisms (Vasan et al., 2024)
2	Ho et al., 2026	Hong Kong, China	Prospective observational (T0, T1)	Older adults experiencing loneliness during COVID-19 pandemic in Hong Kong	Mean 64.1 years; SD 4.38	141	Older adults reporting loneliness; completed questionnaires and saliva sampling at baseline and 6-month follow-up	Loneliness; social network; perceived social support; mindfulness (validated scales)	Salivary cortisol (six samples over two days at T0 and T1); CRP (saliva)	Psychosocial and physiological well-being (loneliness, social relationships, mindfulness, cortisol, CRP)	Path analysis of changes over time (T0–T1)	Small–moderate increases in perceived social support and mindfulness; decreases in loneliness and pre-lunch cortisol; greater improvements in social relationships associated with steeper cortisol slopes; improvements in mindfulness and

												social relationships indirectly linked to greater CRP reductions via decreased loneliness (Ho et al., 2026)
3	Xiang et al., 2025	China / UK	Prospective cohort (UK Biobank)	UK Biobank participants with metabolomics and isolation data	Middle-aged adults; exact mean NR	236,026	Free of VTE at baseline and with complete metabolomics data; social isolation constructed from living alone, friend/family visits, group activities	Social isolation composite (frequency of social contacts, group activities, living alone)	Plasma metabolomic signature (blood metabolites)	Incident VTE (deep vein thrombosis, pulmonary embolism)	Least absolute shrinkage and selection operator (LASSO) to derive isolation-related metabolomic signature; Cox models; mediation analysis	Isolation-associated metabolomic signature predicted incident VTE; hazard ratios for signature estimated (exact values NR); proportion of isolation–VTE association mediated by metabolomic signature quantified (Xiang et al., 2025)
3–4	Shen et al., 2025	UK	Prospective cohort with proteomics and MR (UK Biobank)	Adults aged 40–69 years from 22 centres across UK	40–69 years; mean 56.4	42,062	Participants with plasma proteomics data and social isolation/loneliness measures; genome-wide genotyping and imaging available	Social isolation and loneliness constructs (questionnaire-based)	2,920 plasma proteins (blood proteome); MR-identified proteins (GFRA1,	CVD, T2D, stroke, all-cause mortality	Proteome-wide association study; protein co-expression network analysis; Cox regression; Mendelian randomization	Proteins linked to isolation/loneliness implicated in inflammation, antiviral and complement systems; >50% prospectively associated with

									ADM, FABP4, TNFRSF10A, ASGR1)		and colocalization	CVD, T2D, stroke, mortality over 14 years; MR suggests causal links from loneliness to five proteins; ADM and ASGR1 supported by colocalization; MR-identified proteins partly mediated loneliness–CVD/stroke/mortality relationships (Shen et al., 2025)
4–5	Bajaj et al., 2016	USA	Observational EMA cohorts	PHHP (older adults) and AHAB-II (middle-aged adults)	PHHP: 50–70 years; AHAB-II: 30–54 years	306 (PHHP); 419 (AHAB-II)	Community-dwelling adults enrolled in cohort studies; EMA data and biomarker sampling	Global social support (ISEL); social integration (SNI); proportion of day spent in positive/negative interactions; interactions with close others vs others	IL-6, CRP (blood)	Systemic inflammation	Regression models adjusted for sociodemographic and lifestyle factors	Global support/integration not consistently associated with IL-6 or CRP; in older adults, higher relative frequency of positive interactions (especially with close others) associated with lower IL-6 in fully adjusted models (Bajaj et al., 2016)

4–5	Wang et al., 2025	UK	Exome-wide association study	UK Biobank, white-British ancestry	Median age 58.0 years; 46.2% women	336,115 (loneliness); 346,115 (isolation)	Participants with high-quality autosomal exome sequencing data; variant QC performed	Loneliness and social isolation (questionnaire-based scores)	Protein-coding variants in risk genes; brain structure phenotypes; blood biomarkers	Psychiatric and physical risk phenotypes; brain structures; biomarker profiles	Gene-based and variant-level exome-wide association; brain-wide association; phenome-wide association	Identified 13 risk genes for loneliness and 6 for isolation; brain-wide analysis revealed 47 associations between these genes and brain structure phenotypes critical for social processing; phenome-wide associations mainly with blood biomarkers and cognitive measures; study provides biological insights into loneliness and isolation (Wang et al., 2025)
5	Patapoff et al., 2024	USA	Single-case experimental design (multiple phase changes)	Older adults in remote resilience/self-compassion program	Mean 76.9 years; SD 7.5	43	Older adults willing to participate in remote intervention; repeated assessments across control, intervention,	Loneliness; perceived stress; depression; self-compassion (validated scales)	Blood pressure; inflammatory markers; sleep actigraphy (objective sleep measures)	Stress, depression, loneliness, self-compassion; exploratory physiological outcomes	Multiple-phase-change SCED (control, intervention, follow-up); repeated-measures ANOVA over 12–18 weeks	From control to intervention completion (weeks 0–12): stress $p<0.001$ ($\eta^2=0.15$), depression $p=0.02$ ($\eta^2=0.08$), loneliness

							follow-up phases					p=0.003 (η²=0.18), self-compassion p=0.01 (η²=0.13); post-hoc tests indicate stress ↓ during intervention and loneliness ↓ during control; some improvements in BP, inflammation and sleep in subsample (Patapoff et al., 2024)

Abbreviations: CRP, C-reactive protein; IL-6, interleukin-6; CVD, cardiovascular disease; T2D, type 2 diabetes; VTE, venous thromboembolism; DVT, deep vein thrombosis; MR, Mendelian randomisation; BP, blood pressure; NHANES, National Health and Nutrition Examination Survey; MIDUS, Midlife in the United States study; PHHP, Pittsburgh Healthy Heart Project; AHAB-II, Adult Health and Behavior Project–Phase II; EMA, ecological momentary assessment; ISEL, Interpersonal Support Evaluation List; SNI, Social Network Index; QC, quality control; SCED, single-case experimental design; η², partial eta-squared.

Supplementary Table S3. Study characteristics by population subgroup

Population subgroup	Study (author/year)	Country	Age range / mean	Clinical / contextual features	Social exposure(s)	Biomarker(s)	Primary outcome(s)	Key notes
Older adults – community sample	Koh-Bell et al., 2021	USA	40–89 years; mean 54	Community-dwelling women from NHANES, multiple sociodemographic risk factors	Social isolation via SNI categories	Fibrinogen, CRP	Cancer mortality; inflammatory profile	Highly isolated women more likely to have low income, fewer years of education, low physical activity, obesity and smoking; fibrinogen ↑ with

								isolation; CRP not associated; cancer mortality driven largely by lifestyle and socioeconomic factors (Koh-Bell et al., 2021)
Older adults – mixed gender cohort	Bajaj et al., 2016 (PHHP portion)	USA	50–70 years	Healthy, community-dwelling older adults in PHHP	Global social support and integration; daily positive/negative interactions, especially with close others	IL-6, CRP	Systemic inflammation	Global support/integration not consistently related to inflammation; higher relative frequency of total positive interactions and those with close others linked to lower IL-6, suggesting functional importance of daily social contact quality (Bajaj et al., 2016)
Older adults – lonely during COVID-19	Ho et al., 2026	Hong Kong, China	Mean 64.1 years	Older adults experiencing loneliness during COVID-19 pandemic; follow-up at 6 months	Loneliness; social network; perceived social support; mindfulness	Salivary cortisol (diurnal profiles); CRP	Changes in psychosocial well-being, stress physiology and inflammation	Pandemic-related loneliness context; improvements in social relationships and mindfulness associated with reduced loneliness, healthier cortisol slopes and lower CRP via loneliness; highlights modifiability of physiological stress markers in older adults (Ho et al., 2026)
Older adults – intervention sample	Patapoff et al., 2024	USA	Mean 76.9 years	Older adults receiving remote resilience/self-compassion intervention	Loneliness; chronic stress; depression; self-compassion	Blood pressure; inflammatory markers; sleep actigraphy	Loneliness, stress, depression, self-compassion; exploratory physiological outcomes	Single-case design shows feasibility and preliminary efficacy of remote psychosocial intervention; notable effect sizes for reductions in stress, depression and loneliness; suggests potential physiological benefits for

								aging, socially isolated individuals (Patapoff et al., 2024)
Middle-aged adults – general population	Shen et al., 2025	UK	40–69 years; mean 56.4	Large UK Biobank cohort; broad cardiometabolic and imaging phenotyping	Social isolation and loneliness constructs	2,920 plasma proteins; MR-identified proteins	CVD, T2D, stroke, mortality; brain volumes; other biomarkers	Middle-aged adults provide strong evidence linking loneliness/isolation to proteomic signatures, cardiometabolic events and mortality; MR supports causal pathways; proteins involved in inflammation, antiviral responses and complement systems (Shen et al., 2025)
Middle-aged adults – general population	Zilioli et al., 2021	USA	25–75 years; mean 47.7	MIDUS Refresher biomarker and diary participants	Living alone; social contact frequency; loneliness	CRP, IL-6; salivary cortisol	Diurnal cortisol pattern; systemic inflammation	Demonstrates unique endocrine and immune effects of living alone vs loneliness; living alone associated with dysregulated cortisol and higher CRP; loneliness specifically associated with IL-6 (Zilioli et al., 2021)
Young adults – general population	Vasan et al., 2024	Australia	18–35 years	Young adults undergoing detailed vascular testing; no known CVD	Loneliness; depression; social anxiety	Pulse transit time; endothelial function indices	Early cardiovascular disease markers (arterial stiffness, endothelial function)	Shows that loneliness and depression already relate to subclinical vascular dysfunction in young adults, with loneliness more strongly linked to arterial stiffness and depression to endothelial dysfunction (Vasan et al., 2024)
Adults – mixed, national sample	MacAulay et al., 2024	USA	26–78 years; mean 52.5	MIDUS adults with biomarker data	Loneliness; BMI; number of friends; multimorbidity; depression	IL-6	Gait speed (walking time); systemic inflammation	Illustrates how loneliness in midlife/adulthood relates to inflammation and functional ageing via BMI and IL-6;

					history/symptoms			highlights importance of psychosocial factors alongside traditional risk factors (MacAulay et al., 2024)
Adults – large biobank genetic and proteomic samples	Wang et al., 2025; Xiang et al., 2025	UK / China	Median age 58.0 years (Wang et al.); middle-aged adults (Xiang et al.)	UK Biobank participants with exome sequencing (Wang et al.) or metabolomics (Xiang et al.)	Loneliness; social isolation composites	Protein-coding variants and brain phenotypes (Wang et al.); metabolomic signature (Xiang et al.)	VTE; psychiatric and physical phenotypes; brain structures; blood biomarkers	Provide genetic and metabolomic evidence that loneliness and social isolation have biological underpinnings, influencing thrombotic risk and broader psychiatric and physical phenotypes (Wang et al., 2025; Xiang et al., 2025)

Abbreviations: CRP, C-reactive protein; IL-6, interleukin-6; CVD, cardiovascular disease; T2D, type 2 diabetes; BP, blood pressure; VTE, venous thromboembolism.

Supplementary Table S4. Biomarker panel and analytic methods across studies (including proteomics and genomics)

Biomarker domain	Specific markers	Biological medium	Study (author/year)	Analytic / assay approach (as described)	Notes on interpretation
Classic inflammatory proteins	CRP (including hs-CRP), IL-6, fibrinogen	Blood (plasma/serum)	Koh-Bell et al., 2021; Zilioli et al., 2021; MacAulay et al., 2024; Bajaj et al., 2016; Ho et al., 2026	Standard clinical/laboratory assays (hs-CRP mentioned explicitly; IL-6 and fibrinogen measured as systemic markers)	CRP and IL-6 reflect acute-phase inflammation; fibrinogen mediates coagulation/inflammatory responses; hs-CRP used to capture low-grade chronic inflammation in some cohorts (e.g. older adults)
Stress-axis biomarkers	Salivary cortisol (diurnal slope)	Saliva	Zilioli et al., 2021; Ho et al., 2026	Multiple saliva samples collected over waking hours (diary projects) and across two days (T0, T1); diurnal slope derived from repeated measures	Flatter cortisol slope indicates dysregulated HPA-axis function and chronic stress; improvements in social relationships and mindfulness associated with steeper, healthier slopes (Ho et al., 2026)
Functional biomarkers and vascular indices	Pulse transit time; endothelial function indices; blood pressure	Vascular measures; BP from standard measurements	Vasan et al., 2024; Patapoff et al., 2024	Physiological tests assessing arterial stiffness (pulse transit time) and endothelial function; BP monitored across phases in intervention study	Shorter pulse transit time reflects increased arterial stiffness; endothelial indices capture early vascular dysfunction; BP improvements suggest potential cardiovascular benefits of psychosocial interventions (Vasan et al., 2024; Patapoff et al., 2024)
Multi-protein plasma proteomics	Broad proteomic panel (2,920 plasma proteins)	Blood (plasma)	Shen et al., 2025	Proteome-wide association study and protein co-expression network analysis in UK Biobank; MR and colocalization applied to subset of proteins	Proteins associated with social isolation and loneliness cluster in inflammatory, antiviral and complement pathways; >50% prospectively linked to CVD, T2D, stroke and mortality; MR-identified proteins (GFRA1, ADM, FABP4, TNFRSF10A, ASGR1) show causal roles and mediate cardiometabolic outcomes (Shen et al., 2025)
Plasma metabolomics	Social isolation-associated metabolic signature	Blood (plasma metabolites)	Xiang et al., 2025	High-dimensional metabolomics; LASSO regression used to derive isolation-related metabolic	Metabolomic signature partly mediates the association between social isolation and incident VTE; reflects metabolic pathways through which social disconnection may

				signature; Cox models estimate VTE risk and mediation	influence thrombosis risk (Xiang et al., 2025)
Genomic markers	Protein-coding variants in risk genes (e.g. ANKRD12, RIPOR2, PTEN, ARL8B, NF1, PIMREG, EDARADD, GIGYF1)	DNA (whole exome sequencing)	Wang et al., 2025	Whole exome sequencing with extensive QC; exome-wide association, gene-based tests; brain-wide and phenome-wide association analyses	Identified risk genes for loneliness (13) and social isolation (6); genes associated with brain structures relevant to social processing and with blood biomarkers and cognitive measures; findings support a biological and genetic basis for social isolation and loneliness (Wang et al., 2025)
Composite functional and inflammatory panels	hs-CRP; GDF-15; hs-cTnT; NT-proBNP; gait speed; handgrip strength	Blood; physical performance testing	ActiFE Ulm study (Activity and Function in the Elderly in Ulm), Germany — Braig et al., 2025	Biomarkers assessed at baseline and 3-year follow-up; functional parameters (habitual gait speed, JAMAR dynamometer handgrip strength) measured with standard tests; associations with social isolation (Lubben Social Network Scale) and loneliness (single-item scale) examined via linear and Cox regression, adjusted for age/sex (Model 1) and established confounders (Model 2)	High SI from friends associated with small but significant adverse associations with hs-CRP, GDF-15, and hs-cTnT at follow-up (Model 1); high SI from family associated with NT-proBNP (Model 2); high SI and moderate-to-severe loneliness associated with lower gait speed; high SI overall linked to increased 10-year mortality (HR 1.39, 95% CI 1.15–1.67, Model 2); loneliness showed weaker but related patterns, with SI overall a stronger predictor than loneliness of both biomarker levels and mortality
Sleep and activity biomarkers	Sleep actigraphy (movement-based sleep quality metrics)	Actigraphy device data	Patapoff et al., 2024	Wearable actigraphy over phases; repeated-measures ANOVA to assess sleep quality changes	Some improvements in sleep quality observed in subsample during/after resilience and self-compassion intervention; provide exploratory evidence linking psychosocial changes to sleep parameters (Patapoff et al., 2024)

Abbreviations: CRP, C-reactive protein; hs-CRP, high-sensitivity C-reactive protein; IL-6, interleukin-6; HPA, hypothalamic–pituitary–adrenal; CVD, cardiovascular disease; T2D, type 2 diabetes; VTE, venous thromboembolism; BP, blood pressure; MR, Mendelian randomisation; QC, quality control.

Supplementary Table S5. Preferred Reporting Items for Systematic reviews and Meta-Analyses extension for Scoping Reviews (PRISMA-ScR) Checklist

SECTION	ITEM	PRISMA-ScR CHECKLIST ITEM	REPORTED ON
**TITLE**			
Title	1	Identify the report as a scoping review.	Title page — "A Scoping Review…"
**ABSTRACT**			
Structured summary	2	Provide a structured summary that includes(as applicable): background, objectives, eligibility criteria, sources of evidence, charting methods, results, and conclusions that relate to the review questions and objectives.	Abstract
**INTRODUCTION**			
Rationale	3	Describe the rationale for the review in the context of what is already known. Explain why the review questions/objectives lend themselves to a scoping review approach.	Section 1, Introduction, paras 1–4
Objectives	4	Provide an explicit statement of the questions and objectives being addressed with reference to their key elements (e.g., population or participants, concepts, and context) or other relevant key elements used to conceptualize the review questions and/or objectives.	Section 1, Introduction, final paragraph
**METHODS**			
Protocol and registration	5	Indicate whether a review protocol exists; state if and where it can be accessed (e.g., a Web address); and if available, provide registration information, including the registration number.	No protocol was registered; stated in Section 2.1 and addressed in the Limitations (Section 4).
Eligibility criteria	6	Specify characteristics of the sources of evidence used as eligibility criteria (e.g., years considered, language, and publication status), and provide a rationale.	Section 2.2
Information sources*	7	Describe all information sourcesin the search (e.g., databases with dates of coverage and contact with authors to identify additional sources), as well as the date the most recent search was executed.	Section 2.3; primary databases covered 2015–2025; targeted supplementary search executed October 2026 (Europe PMC)
Search	8	Present the full electronic search strategy for at least 1 database, including any limits used, such that it could be repeated.	Section 2.3; verbatim strings in Supplementary Table S1
Selection of sources of evidence†	9	State the process for selecting sources of evidence (i.e., screening and eligibility) included in the scoping review.	Section 2.4
Data charting process‡	10	Describe the methods of charting data from the included sources of evidence (e.g.,	Section 2.5

SECTION	ITEM	PRISMA-ScR CHECKLIST ITEM	REPORTED ON
		calibrated forms or forms that have been tested by the team before their use, and whether data charting was done independently or in duplicate) and any processes for obtaining and confirming data from investigators.	
Data items	11	List and define all variables for which data were sought and any assumptions and simplifications made.	Section 2.5
Critical appraisal of individual sources of evidence§	12	If done, provide a rationale for conducting a critical appraisal of included sources of evidence; describe the methods used and how this information was used in any data synthesis (if appropriate).	Not performed, consistent with PRISMA-ScR (optional for scoping reviews); see Limitations, Section 4.
Synthesis of results	13	Describe the methods of handling and summarizing the data that were charted.	Section 2.6
**RESULTS**			
Selection of sources of evidence	14	Give numbers of sources of evidence screened, assessed for eligibility, and included in the review, with reasons for exclusions at each stage, ideally using a flow diagram.	Section 3.1; Figure 1 (PRISMA flow diagram)
Characteristics of sources of evidence	15	For each source of evidence, present characteristics for which data were charted and provide the citations.	Section 3.2; Table 1; Supplementary Data (all 73 studies)
Critical appraisal within sources of evidence	16	If done, present data on critical appraisal of included sources of evidence (see item 12).	NR — not performed
Results of individual sources of evidence	17	For each included source of evidence, present the relevant data that were charted that relate to the review questions and objectives.	Sections 3.3–3.8; Tables 1–3
Synthesis of results	18	Summarize and/or present the charting results as they relate to the review questions and objectives.	Sections 3.3–3.8; Figure 3
**DISCUSSION**			
Summary of evidence	19	Summarize the main results (including an overview of concepts, themes, and types of evidence available), link to the review questions and objectives, and consider the relevance to key groups.	Section 4, Discussion, paras 1–4
Limitations	20	Discuss the limitations of the scoping review process.	Section 4, Limitations paragraph
Conclusions	21	Provide a general interpretation of the results with respect to the review questions and objectives, as well as potential implications and/or next steps.	Section 5, Conclusions
**FUNDING**			
Funding	22	Describe sources of funding for the included sources of evidence, as well as sources of funding for the scoping review. Describe the role of the funders of the scoping review.	Funding statement ("did not receive any specific grant...")

JBI = Joanna Briggs Institute; PRISMA-ScR = Preferred Reporting Items for Systematic reviews and Meta-Analyses extension for Scoping Reviews.

* Where *sources of evidence* (see second footnote) are compiled from, such as bibliographic databases, social media platforms, and Web sites.
† A more inclusive/heterogeneous term used to account for the different types of evidence or data sources (e.g., quantitative and/or qualitative research, expert opinion, and policy documents) that may be eligible in a scoping review as opposed to only studies. This is not to be confused with *information sources* (see first footnote).
‡ The frameworks by Arksey and O'Malley (6) and Levac and colleagues (7) and the JBI guidance (4, 5) refer to the process of data extraction in a scoping review as data charting.
§The process of systematically examining research evidence to assess its validity, results, and relevance before using it to inform a decision. This term is used for items 12 and 19 instead of "risk of bias" (which is more applicable to systematic reviews of interventions) to include and acknowledge the various sources of evidence that may be used in a scoping review (e.g., quantitative and/or qualitative research, expert opinion, and policy document).

*From:*Tricco AC, Lillie E, Zarin W, O'Brien KK, Colquhoun H, Levac D, et al. PRISMA Extension for Scoping Reviews (PRISMAScR): Checklist and Explanation. Ann Intern Med. 2018;169:467–473.doi: 10.7326/M18-0850.
